# Integrative multi-omics reveals CUL4B as a modulator of macrophage pyroptosis and innate defense against *Staphylococcus aureus*

**DOI:** 10.1126/sciadv.aeh4023

**Published:** 2026-10-14

**Authors:** Fatemeh Askarian, Daniel Sun, Jasmine Tat, James T. Sorrentino, Armin Kousha, Consuelo Sauceda, Siri Yendluri, Yu Yan, Ka Pui Sharon Yau, Helen Masson, Jacob Wozniak, Kristin Pettersen, Natalie Chavarria, Ella McLaren, Hunter Gage, Alexandria Hoffman, Stanley Davis, Chih-Ming Tsai, Jonathan Monk, Angela Meier, George Sakoulas, Tom Eirik Mollnes, Gustav Vaaje-Kolstad, Ingrid Cornax, Pengbo Zhou, Mona Johannessen, David J. Gonzalez, Nathan E. Lewis, Victor Nizet

**Affiliations:** ^1^ Department of Pediatrics, University of California San Diego, School of Medicine, La Jolla, CA, USA.; ^2^ Department of Bioengineering, University of California San Diego, School of Medicine, La Jolla, CA, USA.; ^3^ Skaggs School of Pharmacy and Pharmaceutical Sciences, UC San Diego, La Jolla, CA, USA.; ^4^ Department of Pathology and Laboratory Medicine, Weill Medical College and Graduate School of Medical Sciences of Cornell University, New York, NY, USA.; ^5^ Department of Pharmacology, University of California, San Diego, La Jolla, CA, USA.; ^6^ Research Laboratory, Nordland Hospital, Bodø, Norway.; ^7^ Paul G. Allen School for Global Health, Washington State University, Pullman, WA 99164.; ^8^ Department of Anesthesiology, Division of Critical Care, University of California San Diego, La Jolla, CA, USA.; ^9^ Department of Immunology, Oslo University, Oslo, Norway.; ^10^ Department of Immunology, Oslo University Hospital, Oslo, Norway.; ^11^ Faculty of Chemistry, Biotechnology and Food Science, Norwegian University of Life Sciences, Ås, Norway.; ^12^ Department of Medical Biology, Faculty of Health Sciences, UiT–The Arctic University of Norway, Tromsø, Norway.; ^13^ Center for Molecular Medicine, Complex Carbohydrate Research Center, and Department of Biochemistry and Molecular Biology, University of Georgia, Athens, GA, USA.

## Abstract

*Staphylococcus aureus* (SA) causes a broad range of invasive human diseases, increasingly complicated by antibiotic resistance. Macrophages (MΦ) play a central role in innate immunity, yet key aspects of their interaction with SA remain incompletely understood because of the pathogen’s diverse immune evasion strategies. Using multi-omics and systems-level analytics, we identified the E3 ubiquitin ligase cullin 4B (CUL4B) as a host factor that modulates MΦ responses to SA infection. CRISPR-mediated deletion of CUL4B in human THP-1 MΦ enhanced intracellular bacterial killing, reduced cell death, decreased caspase-1 activation, and lowered interleukin-1β production, consistent with altered inflammasome signaling. In a murine methicillin-resistant SA (MRSA) pneumonia model, CUL4B deficiency improved pulmonary bacterial clearance, reduced bacteremia, and decreased inflammatory tissue injury. Whole-lung and serum proteomics further revealed increased antimicrobial protein abundance and reduced markers of inflammatory damage. Together, these findings identify CUL4B as a regulator of MΦ inflammatory responses during MRSA infection and suggest a potential host-directed pathway that could be leveraged to improve outcomes against this major pathogen.

## INTRODUCTION

*Staphylococcus aureus* (SA) causes a broad range of invasive diseases, ranging from skin and soft tissue infections to pneumonia, endocarditis, sepsis, and death (*1*). It is the leading cause of hospital-acquired infections, and community-acquired SA infections represent an expanding public health threat (*2*). The increasing prevalence of SA infections is compounded by the rapid emergence of antibiotic-resistant strains, such as methicillin-resistant SA (MRSA), which severely limit treatment options (*2*). While innate immunity serves as the first line of defense, SA uses a broad array of immune evasion strategies, expressing virulence factors that facilitate cytotoxicity, adherence, invasion, inflammation, molecular mimicry, and resistance to immune clearance (*1*, *3*–*6*).

Professional phagocytes play a central role in host defense by recognizing, engulfing, and eliminating bacterial pathogens (*7*). Macrophages (MΦ), a key professional phagocyte population, are equipped with antimicrobial effector mechanisms essential for orchestrating the immune response (*4*, *7*). In addition to pathogen clearance, MΦ remove apoptotic or necrotic cells, including infected neutrophils, through efferocytosis (*8*). However, SA has evolved mechanisms to evade MΦ antimicrobial defenses, enabling intracellular persistence even within mature phagolysosomes (*4*, *5*, *9*–*15*). This intracellular survival shields SA from immune clearance, promoting persistence (*10*, *16*) and systemic dissemination (*17*, *18*).

A key component of SA pathogenesis is its ability to manipulate host cell death pathways to establish and spread infection (*19*). SA triggers NLRP3 inflammasome activation and pyroptosis (*20*–*24*), a caspase-1–mediated inflammatory cell death pathway, in MΦ (*25*–*27*), human and mouse monocytes (*20*), and nonphagocytic cells such as keratinocytes (*28*). Pyroptotic MΦ contribute to host defense through Gasdermin D–mediated cell lysis and the release of proinflammatory cytokines [interleukin-1β (IL-1β)/IL-18], which are regulated by caspases-1, -4, -5, and -11 (*29**–**33*). However, when inflammasome activation becomes excessive, it can impair effective host defense. SA disrupts MΦ-mediated killing by modulating mitochondrial trafficking through NLRP3 (*27*), and inhibition of NLRP3 enhances SA clearance by primary human monocytes and murine MΦ (*27*). In addition, deletion or inhibition of *Nlrp3* in vivo protects mice from lethal or severe SA pneumonia (*27*, *34*).

Compared with neutrophils, MΦ interactions with SA remain less well characterized despite evidence that MΦ depletion increases host susceptibility to SA infection in vivo (*35**–**37*). Advances in global analyses of translational, posttranslational, and transcriptional immune responses have expanded our understanding of host-pathogen interactions. In this study, we used integrative multi-omics approaches combined with systems-level analytics to identify regulators that modulate MΦ responses to MRSA infection. We further assessed whether these host-derived signatures enhance MΦ antimicrobial activity and MRSA clearance both in vitro and in vivo. Through these analyses, we identified E3 ubiquitin ligase cullin 4B (CUL4B) as a candidate regulator of MΦ innate immune responses.

CUL4B serves as a scaffold for CUL4B-RING E3 ligase (CRL4B) complexes that mediate ubiquitination of diverse substrates and regulate multiple cellular processes, including immune signaling (*38*, *39*). CUL4B has been implicated in extraembryonic tissue development (*40*, *41*), obesity (*42*), X-linked intellectual disabilities (*43*, *44*), sperm development and male fertility (*45*, *46*), intestinal homeostasis (*47*), carcinogenesis (*48**–**50*), viral replication (*51*, *52*), differentiation and function of immune cells (*39*, *49*, *53**–**58*), and diabetic kidney disease (*55*). In addition, several E3 ubiquitin ligases regulate NLRP3 inflammasome activity. For example, TRIM31 (*59*), Cbl-b (*60*), RNF125 (*60*), MARCH7 (*61*), SCFFBXL2 (*62*), ARIH2 (*63*), β-TrCP1 (*64*), cullin1 (*65*), and Pellino (*66*) function as negative or positive regulators of NLRP3 signaling. Here, we applied a multi-omics platform combined with targeted mutagenesis to identify a role for CUL4B in regulating MΦ inflammatory responses during MRSA infection.

## RESULTS

### Multi-omics analysis identifies key regulators of MΦ responses to intracellular MRSA infection

To define molecular programs governing MΦ responses to MRSA infection, we performed integrated transcriptomic and proteomic profiling of THP-1–derived MΦ across a temporal infection course (Supplementary Results and figs. S1 to S4). Cells were either left untreated or infected with MRSA (strain TCH1516) for 1, 2, 4, 7, and 24 hours postinfection (hpi).

Principal component analysis demonstrated strong coherence among biological replicates and revealed distinct temporal responses in the proteome (Fig. 1A) and transcriptome (Fig. 1B) of MRSA-infected MΦ compared with mock-infected controls. The IPA regulator analysis identified seven shared upstream regulators (dataset S7, network bias–corrected *P* values ≤0.05, *z* score ≥2) across all examined time points, including Rho-associated coiled-coil kinase, Ras-related C3 botulinum toxin substrate, ataxia-telangiectasia–mutated, protein kinase G, MYC proto-oncogene, amphiregulin, and CUL4B (Fig. 1C and fig. S1G). Among these, CUL4B uniquely displayed sustained predicted activation throughout the course of infection, highlighting it as a candidate regulator of MΦ responses to MRSA infection (Fig. 1C).

**Fig. 1.**
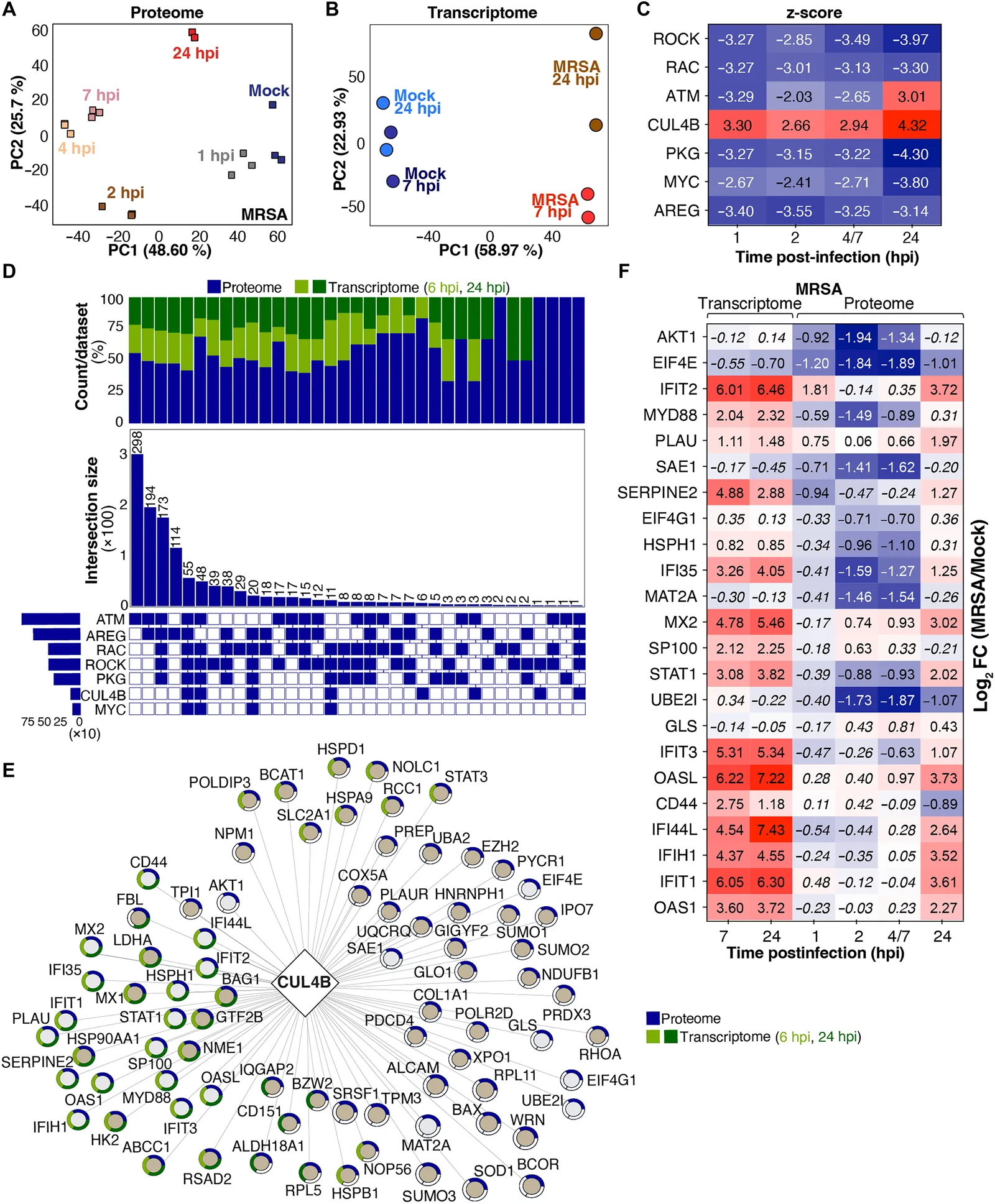
Integrative multi-omics and IPA analyses identify CUL4B activation during intracellular MRSA infection of MΦ. (**A** and **B**) Principal components analysis (PCA) of global (A) proteomic and (B) transcriptomic datasets from MΦ following MRSA infection. MΦ were infected with MRSA (1 hour; MOI = 5), washed, and incubated in medium containing gentamicin and lysostaphin [antibiotics (ABX)]. Proteins and transcripts from biological replicates were projected into two-dimensional principal component space to visualize clustering by infection status and hpi. (**C**) Heatmap of IPA upstream regulator predictions shared across postinfection time points. Regulators with significant predicted activation or inhibition (*z* score ≥ 2 or ≤ −2; bias-corrected *P* ≤ 0.05; fig. S1G) are shown (dataset S7). IPA was performed using significantly regulated proteins (dataset S2 and fig. S6). (**D**) UpSet plot illustrating overlap among significantly regulated genes (FC ≥ 1.5 or FC ≤ −1.5; adjusted *P* ≤ 0.05) and proteins (FC ≥ 1.5 or FC ≤ −1.5; adjusted all *P* ≤ 0.05) associated with the predicted upstream regulators (C). Horizontal bars indicate total targets per regulator; connected squares (dark blue) show shared intersections (dataset S8). (**E**) Network of significantly regulated genes and proteins associated with CUL4B detected across time points [described in (F) and fig. S7B]. Node borders indicate the dataset source (green, transcriptome; blue, proteome). Light gray nodes represent shared targets (F) among regulators in (C), whereas dark gray nodes represent CUL4B-specific targets (fig. S7B) (datasets S9 and S10). (**F**) Heatmap showing mean log_2_ fold changes of significantly regulated genes and proteins shared among predicted regulators. Nondifferentially expressed targets at individual time points are italicized (dataset S9).

Cytoscape network analysis revealed a high degree of interconnectivity among these regulators and their association with a subset of significantly altered proteins in the proteome dataset (fig. S6). The UpSet plot further illustrated the overlap between regulated proteins and genes linked to these regulators in MRSA- versus mock-infected MΦ (Fig. 1D and dataset S8). Notably, CUL4B activation was strongly linked to 79 significantly regulated proteins and genes across one or more time points (Supplementary Results, Fig. 1E, and fig. S7B), including 23 targets shared across all identified regulatory networks (Fig. 1, E and F; nodes marked in light gray). Functional enrichment analysis of shared genes and proteins (Fig. 1F; nondifferentially expressed genes and proteins in italics) indicated association with interferon (IFN) signaling pathways (fig. S7A), consistent with coordinated innate immune activation during MRSA infection (*67**–**70*). These findings were further supported by independent validation experiments confirming activation of IFN-I–associated signaling pathways during intracellular MRSA infection (Supplementary Results and fig. S5). Together, these results reveal complex regulatory networks shaping MΦ responses to MRSA infection and identify CUL4B as a prominent modulator of the host response, prompting further mechanistic investigation.

### MRSA modulates MΦ CUL4B expression

To further investigate the role of CUL4B in MΦ immune modulation during MRSA infection and to explore its potential mechanism of action, we used CRISPR-Cas9 genome editing to generate a targeted deletion of *CUL4B* in the human MΦ cell line THP-1. A guide RNA was designed to target exon five of *CUL4B* (fig. S8, A and B). Loss of CUL4B expression following clonal selection was confirmed by sequencing (fig. S8C), immunoblotting (fig. S8D, top), and reverse transcription polymerase chain reaction (RT-PCR) (fig. S8D, bottom). Clone 7 was selected for subsequent analyses (fig. S8D). High-resolution tandem mass tag (TMT)–based quantitative proteomics was used to assess CUL4B expression in wild-type (WT) and ΔCUL4B MΦ following MRSA internalization [multiplicity of infection (MOI) = 10, 4 hpi]. CUL4B protein levels were significantly increased in MRSA-infected MΦ compared with mock-infected controls (Fig. 2A, left, and dataset S14) (*P* < 0.05). Notably, *CUL4B* deletion did not affect expression of its paralog CUL4A (Fig. 2A, right), which shares ∼82% sequence identity (*71*). Moreover, deletion of CUL4B did not alter expression of other cullin-RING ubiquitin ligase family members (e.g., CUL1, CUL2, CUL3, and CUL5) in either mock- or MRSA-infected WT and ΔCUL4B MΦ (fig. S9A and dataset S14).

**Fig. 2.**
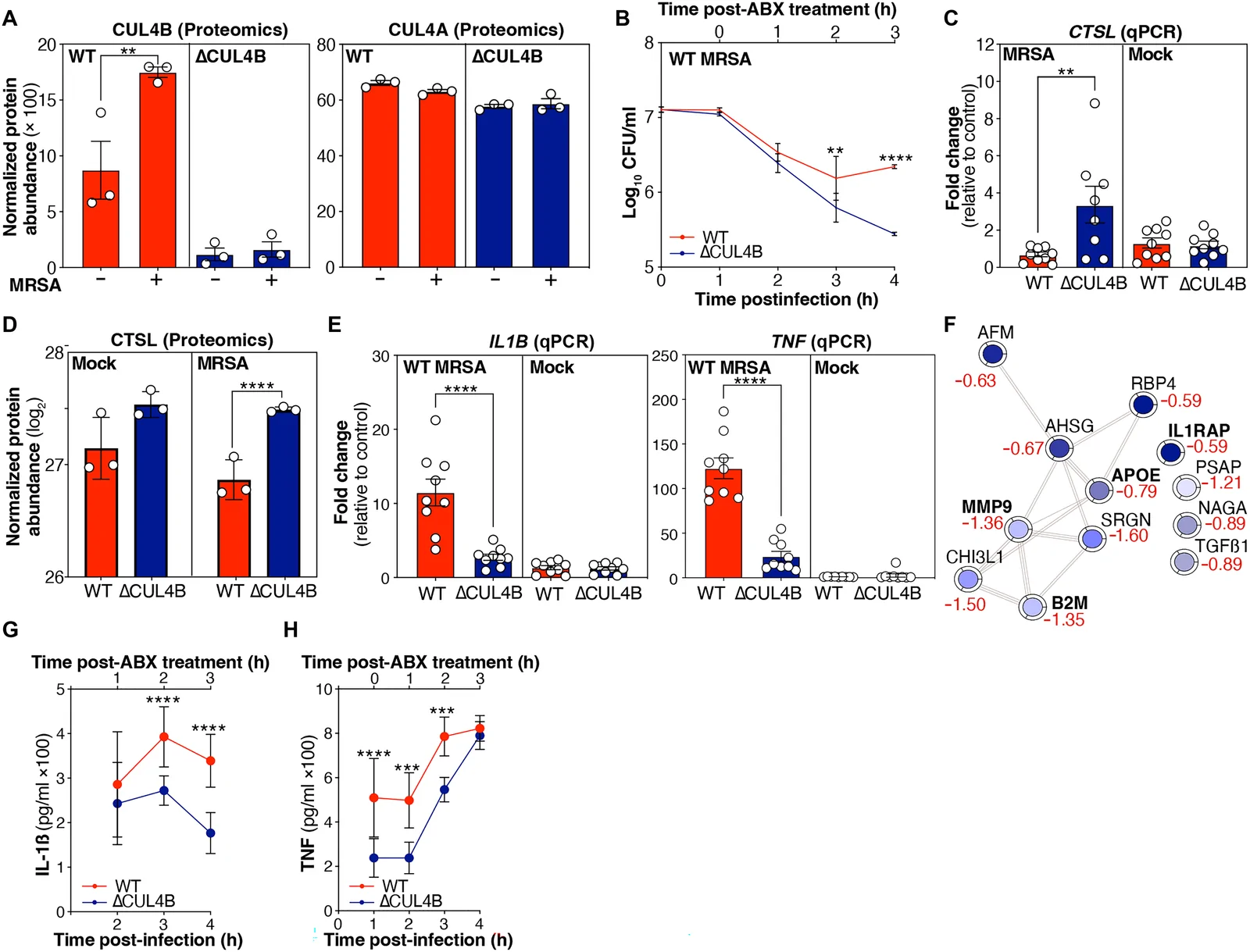
Deletion of CUL4B promotes MΦ resilience to MRSA infection. (**A**) Protein abundance of CUL4B and CUL4A in infected MΦ. Cells were infected [1 hour (h)], treated with ABX, and lysed at 4 hpi for liquid chromatography–tandem mass spectrometry–based quantitative proteomics. Data represent means ± SEM from three biological replicates. CUL4B: *P =* 0.0047. (**B**) Intracellular MRSA killing by MΦ. Bacteria were quantified by cell lysis and enumeration. Data represent means ± SD from three experiments. 3 hpi: *P =* 0.0053, 4 hpi: *P* < 0.0001. (**C**) Relative *CTSL* mRNA expression (2^−ΔΔCq^) at 3 hpi (MOI = 10). Data represent means ± SEM from three duplicate/triplicate experiments and were normalized to glyceraldehyde-3-phosphate dehydrogenase (*GAPDH*). MRSA: *P* = 0.0033. (**D**) CTSL protein abundance (log_2_) in ΔCUL4B versus WT MΦ at 3 hpi. Data represent means ± SEM from three biological replicates (datasets S15 and S16). MRSA: adjusted *P* < 0.0001. (**E**) Relative *TNF* and *Il1B* mRNA expression (2^−ΔΔCq^) at 1 hpi. Data represent means ± SEM from three triplicate experiments and were normalized to *GAPDH*. *TNF*: *P* < 0.0001; *Il1B*: *P* < 0.0001. (**F**) STRING analysis of significantly down-regulated secretome proteins (dataset S13) in ΔCUL4B- versus WT-infected MΦ. IL-1 signaling proteins are bolded. Node color represents log_2_ fold change. (**G** and **H**) IL-1β and TNF levels in infected MΦ supernatants were quantified by enzyme-linked immunosorbent assay. Data represent means ± SD from three (H) and six (G) duplicate/triplicate experiments. IL-1β: 3 hpi: *P* < 0.0001, 4 hpi: *P* < 0.0001; TNF: 1 hpi: *P* < 0.0001, 2 hpi: *P* = 0.0002, 3 hpi: *P* = 0.0005. Data were analyzed by two-way analysis of variance (ANOVA) with Tukey’s multiple comparisons test (A, C, and E) and Šídák’s multiple comparisons test (B, G, and H). Significance: **P ≤ 0.01; ***P ≤ 0.001; ****P ≤ 0.0001.

### Deletion of CUL4B enhances MΦ bactericidal function against MRSA

To assess the impact of CUL4B deletion on MΦ bactericidal function, WT and *CUL4B*-deficient MΦ were challenged with MRSA for 1 hour, followed by antibiotics treatment to eliminate extracellular bacteria and prevent their replication. This approach ensured that subsequent colony-forming unit (CFU) measurements reflected intracellular bacterial burden.

WT and ΔCUL4B MΦ exhibited comparable viability across all examined time points (fig. S9B). However, loss of *CUL4B* was associated with significantly enhanced intracellular bacterial killing at both 3 and 4 hpi (*P* < 0.05) (Fig. 2B). ΔCUL4B MΦ also exhibited enhanced killing of two additional clinical MRSA strains (fig. S9C) (*P* < 0.05). Further analysis indicated that improved bacterial killing in ΔCUL4B MΦ was not attributable to differences in phagocytic capacity, as similar CFU counts were recovered from WT and mutant MΦ at the earliest time points (Fig. 2B). Nor was the phenotype explained by differences in reactive oxygen species production, as measured by oxidation of DCFH-DA (fig. S9D). In contrast, CUL4B deletion did not alter clearance of other Gram-positive bacterial pathogens, including *Streptococcus pyogenes* (fig. S9E) and *Streptococcus agalactiae* (fig. S9F), for which MΦ play an established role in host defense [e.g., (*72*, *73*)]. These findings suggest that CUL4B activity selectively restrains MΦ bactericidal capacity during MRSA infection.

Given the contributions of lysosomal cathepsins to MΦ antimicrobial defense, we next examined whether CUL4B deletion was associated with altered expression of selected lysosomal proteases. Transcription and translation of CTSL, CTSD, and CTSB were evaluated using RT–quantitative (qPCR) (with glyceraldehyde-3-phosphate dehydrogenase as reference gene) and proteomics analysis. Intriguingly, loss of CUL4B was accompanied by increased *CTSL* transcription (Fig. 2C) in response to MRSA (3 hpi, MOI = 10). *CTSL* encodes cathepsin L, a lysosomal acidic protease previously implicated in nonoxidative killing of SA within MΦ (*74*). Under basal conditions, CUL4B deficiency resulted in a modest increase in CTSL protein abundance compared with WT MΦ (Fig. 2D and datasets S15 and S16). Following MRSA infection (MOI = 10, 3 hpi), ΔCUL4B MΦ displayed a significant elevation in CTSL protein abundance relative to WT cells (Fig. 2D and datasets S15 to S16), coinciding with enhanced bacterial clearance. By contrast, transcription (fig. S9, G and H) and translation (dataset S15) of CTSB and CTSD, which encode additional antimicrobial lysosomal proteases, were unchanged. This suggests a selective effect rather than a general enhancement of lysosomal protease expression. Pretreatment of WT and ΔCUL4B MΦ with bafilomycin before MRSA internalization (3 hpi, MOI = 10) did not abolish the enhanced bactericidal capacity of ΔCUL4B cells (fig. S9I), indicating that the MRSA killing advantage is at least partially independent of lysosomal acidification and may involve additional antimicrobial pathways.

Consistent with this broader reprogramming, quantitative proteomic analysis identified increased abundance of several antimicrobial-associated proteins in CUL4B-deficient MΦ, including S100 calcium–binding protein A8 (S100A8) and S100 calcium–binding protein-A9 (S100A9) under both basal and MRSA-infected conditions (fig. S9J and datasets S15 and S16). These proteins have been implicated in MRSA clearance (*75*) and MΦ functions (*75*, *76*). Together, these findings indicate that loss of CUL4B is associated with enhanced MRSA killing and selective changes in antimicrobial effector expression, including CTSL and S100A8/A9.

### Deletion of CUL4B reduces inflammatory cytokine production in MRSA-infected MΦ

Deletion of *CUL4B* in MRSA-infected MΦ (MOI = 10) led to decreased transcription of *IL-1B* and tumor necrosis factor (*TNF*) (Fig. 2E)*.* TMT-based quantitative proteomics of the secretome, combined with STRING network analysis, revealed significant down-regulation (FC ≤ −1.5 and *P* ≤ 0.05) of interconnected proteins in ΔCUL4B- versus WT-infected (MOI = 10, 4 hpi) THP-1 cells (datasets S12 and S13). Several of these proteins were linked to IL-1 signaling (e.g., IL1RAP, MMP9, APOE, and B2M) or broader regulation of inflammatory responses [e.g., transforming growth factor–β (TGF-β), and CHI3L1] (Fig. 2F and dataset S13).

Consistent with these findings, quantification of proinflammatory markers in culture supernatants of MRSA-infected WT and ΔCUL4B MΦ (MOI = 10) revealed significantly reduced secretion of TNF (1, 2, and 3 hpi), a general readout of Toll-like receptor (TLR) signaling (*77*), and IL-1β (3 and 4 hpi), a canonical readout of inflammasome activation, in ΔCUL4B MΦ compared with WT controls (*P* < 0.05) (Fig. 2, G and H). In addition, secretion of IP-10, a marker of IFN signaling, was significantly decreased in MRSA-infected ΔCUL4B MΦ (fig. S9K) (*P* < 0.05). To further assess this phenotype in a primary MΦ system, bone marrow–derived MΦ (BMDM) from WT and ΔCUL4B mice were infected with MRSA. IL-1β secretion profiling revealed significantly increased IL-1β production by WT murine MΦ at 3 hpi compared with ΔCUL4B cells (fig. S9L) (*P* < 0.05). Together, these findings indicate that CUL4B promotes inflammatory cytokine production, including the inflammasome-dependent cytokine IL-1β, during MRSA infection in both THP-1-derived and murine MΦ.

### CUL4B deletion remodels the MΦ proteome and reduces inflammasome-associated proteins

Since IL-1β is a key cytokine released during pyroptosis, we investigated whether CUL4B deletion alters the translational regulation of proteins linked to inflammatory cell death in MRSA-infected ΔCUL4B versus WT MΦ (datasets S15 and S16). Proteomics analysis revealed significant regulation (FC ≥ 1.5 or FC ≤ −1.5 and adjusted *P* ≤ 0.05) of 216 proteins in mock-infected ΔCUL4B compared to WT THP-1 cells (dataset S16). Down-regulated proteins in ΔCUL4B MΦ were strongly enriched for pathways associated with cellular response to virus, type I IFN production, and acute inflammatory responses (Metascape analysis; *P* value cutoff = 0.01; fig. S10A, top). This signature was driven by coordinated reduction of key antiviral effectors including ISG15, IFI44, IFIT2, IFIT1, IFIH1, IFITM3, and IFIT3 (dataset S16), along with chemokines and signaling mediators such as CXCL10, TGF-β1, TLR2, and CD209 (dataset S16). Additional down-regulated terms included glycosaminoglycan metabolisms and complement/coagulation cascades (Metascape analysis; fig. S10A, top). Conversely, proteins increased in ΔCUL4B MΦ were enriched in pathways related to lipid metabolism, mitophagy, peroxisome proliferator–activated receptor signaling, and detoxification processes, and included factors associated with negative regulation of inflammatory responses (fig. S10A, bottom), such as SELENOS, NDFIP1, CD180, and NQO1 (dataset S16). Collectively, these findings indicate that CUL4B deletion reconfigures the basal MΦ proteome toward reduced antiviral and inflammatory signaling and increased representation of metabolic and immunomodulatory pathways.

During MRSA infection, proteomics analysis revealed significant regulation of 366 proteins in ΔCUL4B versus WT THP-1 MΦ (dataset S16). Among these, 84 proteins (excluding CUL4B) displayed a similar expression pattern in both mock- and MRSA-infected ΔCUL4B MΦ relative to WT cells (fig. S10B and dataset S16). Analysis of the infection-specific proteome using XdeathDB (*78*) and Harmonizome datasets (KEGG pathway hsa04225) (*79*, *80*) identified 19 pyroptosis-associated proteins, including the inflammasome sensor NLRP3, with significantly altered abundance in MRSA-infected ΔCUL4B compared with WT MΦ (Fig. 3A and dataset S16). STRING network analysis further demonstrated that 12 of these proteins, including CD53, NLRP3, FBLN1, FN1, PTPRK, HAVCR2, C3AR1, SERPINE2, SMAD4, TFPI2, and MYH10, formed an interconnected network linked to IL-1β (fig. S10C), aligning with the reduced IL-1β secretion observed in MRSA-infected ΔCUL4B MΦ (Fig. 2G). Because inflammasome activation promotes inflammatory cytokine release but can also compromise MΦ survival during intracellular infection, we next examined whether reduced inflammasome signaling in ΔCUL4B MΦ alters pyroptotic cell death during MRSA infection.

**Fig. 3.**
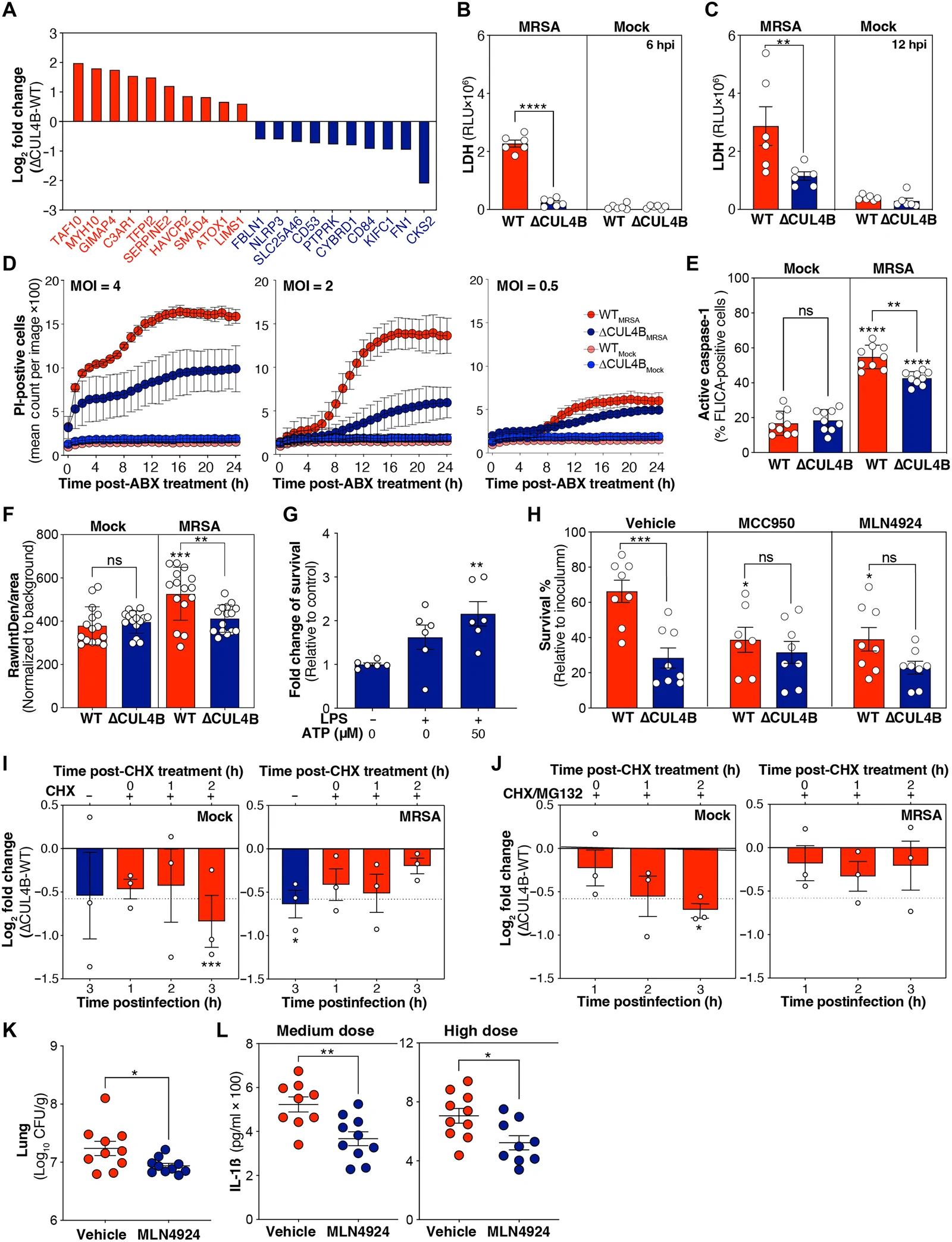
Deletion of CUL4B mitigates cell death in MRSA-infected MΦ. (**A**) Average log_2_ fold changes of significantly regulated cell death–associated proteins in MRSA-infected ΔCUL4B versus WT MΦ (dataset S16). (**B** and **C**) LDH release from MRSA-infected WT and ΔCUL4B MΦ at 6 and 12 hpi. Data represent means ± SEM from three duplicate experiments. (B) *P* < 0.0001; (C) *P* = 0.0043. (**D**) Real-time viability of infected MΦ was monitored by IncuCyte imaging with PI staining for 24 hours. Mock controls shared across MOIs. Data represent means ± SD from a representative triplicate experiment (*n* = 2). (**E** and **F**) Activated caspase-1 was quantified by FAM-FLICA staining and analyzed by flow cytometry (E) or fluorescence microscopy (F). Data represent means ± SEM from three experiments. (E) WT versus WT_MRSA_: *P* < 0.0001, ΔCUL4B versus ΔCUL4B_MRSA_: *P* < 0.0001, WT_MRSA_ versus ΔCUL4B_MRSA_: *P* = 0.0025; (F) WT_MRSA_ versus ΔCUL4B_MRSA_: *P* = 0.0028, WT versus WT_MRSA_: *P* = 0.001. (**G**) LPS/ATP-primed ΔCUL4B MΦ were infected with MRSA, and intracellular survival was quantified. Data represent means ± SEM from two triplicate experiments. Control versus LPS^+^/ATP^+^: *P* = 0.007. (**H**) Intracellular MRSA survival in MΦ following MCC950 and MLN4924 treatment. Data represent means ± SEM from three duplicate/triplicate experiments. Vehicle: *P* = 0.0008, WT_Vehicle_ versus WT_MCC950_: *P* = 0.0434, WT_Vehicle_ versus WT_MLN4924_: *P* = 0.0354. (**I** and **J**) NLRP3 protein abundance following CHX ± MG132 treatment in response to MRSA was determined by proteomics. Untreated infected cells are shown in (A). Data represent means ± SEM from three biological replicates (datasets S16 and S17). (I) Mock: adjusted *P =* 0.007, MRSA: adjusted *P =* 0.037. (J) Mock: adjusted *P =* 0.0303. (**K** and **L**) MLN4924- or vehicle-treated C57BL/6J mice were infected IT with MRSA. Lung bacterial burden (K) and IL-1β (L) were measured 24 hpi. Data represent means ± SEM from 9 to 10 mice per group and were analyzed by the two-tailed *t* test. (K) *P* = 0.0317; (L) medium dose: *P* = 0.0039, high dose: *P* = 0.0170. Data were analyzed by two-way ANOVA with Tukey’s multiple comparisons test (E to G) and Šídák’s multiple comparisons test (B, C, and H). Significance: *P ≤ 0.05; **P ≤ 0.01; ***P ≤ 0.001; ****P ≤ 0.0001; ns, *P* > 0.05.

### CUL4B promotes inflammasome activation and pyroptotic MΦ death during MRSA infection

To determine whether the observed proteomic changes influence MΦ viability during infection, MRSA infection was extended beyond 4 hours to assess cell death progression. Following MRSA internalization (MOI = 10), WT MΦ exhibited significantly increased lactate dehydrogenase (LDH) release at both 6 and 12 hpi compared with ΔCUL4B cells (Fig. 3, B and C). Real-time viability monitoring using the Incucyte live-cell imaging system with propidium iodide (PI) staining revealed higher levels of PI-positive cells in WT MΦ compared with ΔCUL4B MΦ in an MRSA MOI–dependent manner (Fig. 3D and fig. S11A), whereas viability remained comparable between genotypes under mock-infected conditions (Fig. 3D).

Because IL-1β maturation requires caspase-1 activation (*81*), we next quantified caspase-1 activity using FAM-YVAD-FMK staining. Flow cytometry confirmed robust caspase-1 activation in MRSA-infected MΦ (MOI = 5, 6 hpi) relative to mock-infected controls (*P* < 0.05) (Fig. 3E). Notably, WT MΦ showed a significantly higher proportion of caspase-1–positive cells compared with ΔCUL4B cells (Fig. 3E). These findings were further validated by microscopy (MOI = 2.5, 6 hpi) (Fig. 3F and fig. S11B) and immunoblotting, which demonstrated higher levels of cleaved caspase-1 (p20) in WT cells (fig. S11, C and D). Given prior evidence that excessive NLRP3 inflammasome activation can impair MΦ control of MRSA infection (*27*), we next examined whether inflammasome activation modulates the enhanced bactericidal phenotype of ΔCUL4B MΦ. ΔCUL4B MΦ were stimulated using the canonical two-signal inflammasome activation model consisting of lipopolysaccharide (LPS) priming followed by low-dose adenosine 5′-triphosphate (ATP) stimulation, conditions selected to minimize cytotoxicity while preserving MΦ viability during MRSA infection. Cell viability remained comparable across treatment conditions before and after MRSA infection (fig. S9M). Under these conditions, inflammasome-primed ΔCUL4B MΦ exhibited a significantly reduced capacity for intracellular MRSA killing (MOI = 10, 3 hpi) (Fig. 3G).

Consistent with the functional role for inflammasome signaling in intracellular MRSA control, pharmacological inhibition of NLRP3 using MCC950 (Fig. 3H) significantly enhanced intracellular MRSA killing in WT MΦ (3 hpi, MOI = 10), reaching levels comparable to those observed in ΔCUL4B cells. Similarly, inhibition of caspase-1 using VX-765 (fig. S12A) increased intracellular MRSA clearance and phenocopied the enhanced bactericidal activity of ΔCUL4B MΦ. These findings indicate that suppression of inflammasome signaling enhances MΦ control of intracellular MRSA. To determine whether upstream innate immune pathways contribute to this response, we next examined TLR2 signaling. TLR2 inhibition (TL2-C29) did not further improve bacterial killing in WT MΦ but abolished the enhanced killing phenotype of ΔCUL4B cells. These data indicate that TLR2-dependent signaling is required for the antibacterial effect associated with CUL4B deficiency (fig. S12A).

Together, these findings indicate that CUL4B promotes inflammasome activation and pyroptotic MΦ death during MRSA infection, whereas limiting inflammasome activation in ΔCUL4B MΦ preserves MΦ viability and enhances intracellular bacterial clearance. The observation that pharmacological inhibition of either NLRP3 or caspase-1 recapitulates the enhanced bactericidal activity of ΔCUL4B MΦ, together with the requirement for TLR2-dependent signaling, supports a functional connection between CUL4B-regulated inflammatory pathways and MΦ control of intracellular MRSA.

### Reduced NLRP3 abundance in ΔCUL4B MΦ is not explained by transcriptional or proteasomal regulation

Proteomic analysis revealed reduced NLRP3 abundance in ΔCUL4B MΦ relative to WT cells, reaching statistical significance during MRSA (3 hpi, MOI = 10) infection (FC ≤ −1.5 and adjusted *P* ≤ 0.05) (Fig. 3I and datasets S16 and S17). We next examined whether this difference reflects altered transcription or protein stability. RT-qPCR revealed no significant differences in NLRP3 mRNA levels between WT and ΔCUL4B MΦ under either mock- or MRSA-infected condition (3 hpi, MOI = 10) (fig. S9N). To assess whether altered protein degradation contributes to reduced NLRP3 abundance, cycloheximide (CHX) chase experiments were performed. Following blockade of translation, the relative difference in NLRP3 abundance between ΔCUL4B and WT MΦ remained largely stable during MRSA infection (3 hpi, MOI = 10), indicating no evidence for accelerated NLRP3 degradation in ΔCUL4B cells (Fig. 3I and datasets S16 and S17). A similar pattern was observed under mock conditions, although NLRP3 abundance in ΔCUL4B MΦ became more negative at later time points following CHX treatment, reaching statistical significance at 2 hours, but without progressive decline across the chase (Fig. 3I and datasets S16 and S17). Consistent with these findings, inhibition of proteasomal activity using MG132 failed to restore NLRP3 abundance in ΔCUL4B MΦ (Fig. 3J and datasets S16 and S17). Together, these results indicate that reduced NLRP3 abundance in ΔCUL4B MΦ is unlikely to arise from transcriptional regulation or enhanced proteasome-dependent degradation, suggesting alternative regulatory mechanisms such as posttranslational control or indirect modulation through upstream signaling networks.

### Cullin-RING ligase inhibition reduces inflammasome activation and improves bacterial clearance in MRSA pneumonia

To test whether the phenotype observed with CUL4B deletion could be reproduced pharmacologically, we used MLN4924, an inhibitor of the NEDD8-activating enzyme that blocks cullin neddylation and thereby inactivates cullin-RING ligases (CRLs), including those containing CUL4B. WT MΦ were pretreated with MLN4924 before MRSA infection, and IL-1β secretion was measured as a readout of inflammasome activation. CRL inhibition significantly reduced IL-1β secretion in a dose-dependent manner (fig. S12B, left), with effects comparable to those observed with NLRP3 inhibitor MCC950 (fig. S12B, right). Consistent with reduced inflammasome activation, inhibition of CRLs and NLRP3 enhanced intracellular MRSA killing (3 hpi, MOI = 10) in WT MΦ, reaching levels comparable to those observed in ΔCUL4B cells (Fig. 3H).

Because neutrophils play a critical role in host defense against SA, we next examined whether CRL inhibition alters neutrophil bactericidal activity (fig. S12C). Opsonized MRSA were efficiently killed by both MLN4924-treated (1 and 10 μM) and vehicle-treated human neutrophils. Although MRSA survival was modestly increased in neutrophils exposed to the higher MLN4924 dose, overall bactericidal activity remained robust (fig. S12C).

Previous studies have shown that deletion or inhibition of *Nlrp3* improves bacterial clearance and reduces mortality in SA pneumonia (*27*, *34*). To determine whether CRL influences disease progression in vivo, mice were treated with MLN4924 by intraperitoneal injection before intratracheal (IT) MRSA challenge. At 24 hpi, bacterial CFUs recovered from the lungs were significantly lower in MLN4924-treated mice compared with controls (Fig. 3K). In parallel, MLN4924 treatment significantly reduced IL-1β levels in lung homogenates measured 24 hpi in mice challenged with two distinct MRSA inocula (Fig. 3L). Consistent with these effects on bacterial burden and inflammatory cytokine production, MLN4924 treatment was associated with a trend toward increased survival following lethal MRSA pneumonia, with approximately 43% of treated mice surviving infection, whereas all vehicle-treated mice were dead within 24 hpi (fig. S12D). Together, these findings indicate that pharmacological inhibition of CRL activity suppresses inflammasome-associated inflammation and improves bacterial clearance during MRSA pneumonia, highlighting cullin-RING ligases as potential targets for host-directed therapeutic strategies.

### CUL4B activity exacerbates MRSA pneumonia in vivo

Given the enhanced bacterial clearance observed following CRL inhibition in MRSA pneumonia (Fig. 3K), we further investigated whether CUL4B itself contributes to MRSA pathogenesis in vivo. Using a murine IT challenge model with a high-dose MRSA inoculum, WT and ΔCUL4B mice (*41*) were infected and disease progression assessed 24 hpi. At this time point, CUL4B-deficient mice exhibited significantly improved pulmonary bacterial clearance compared with WT controls (*P* < 0.05) (Fig. 4A). Serum LDH levels, used as a marker of tissue injury, were modestly higher in WT mice but did not differ significantly between groups (Fig. 4B). Complete blood count analysis revealed minor, nonsignificant elevations in white blood, lymphocyte, and monocyte counts in ΔCUL4B mice (fig. S13A). In addition, WT mice displayed a higher frequency of bacteremia (100%) (fig. S13B) and increased bacterial loads in blood (fig. S13C), consistent with less effective containment of infection following IT challenge.

**Fig. 4.**
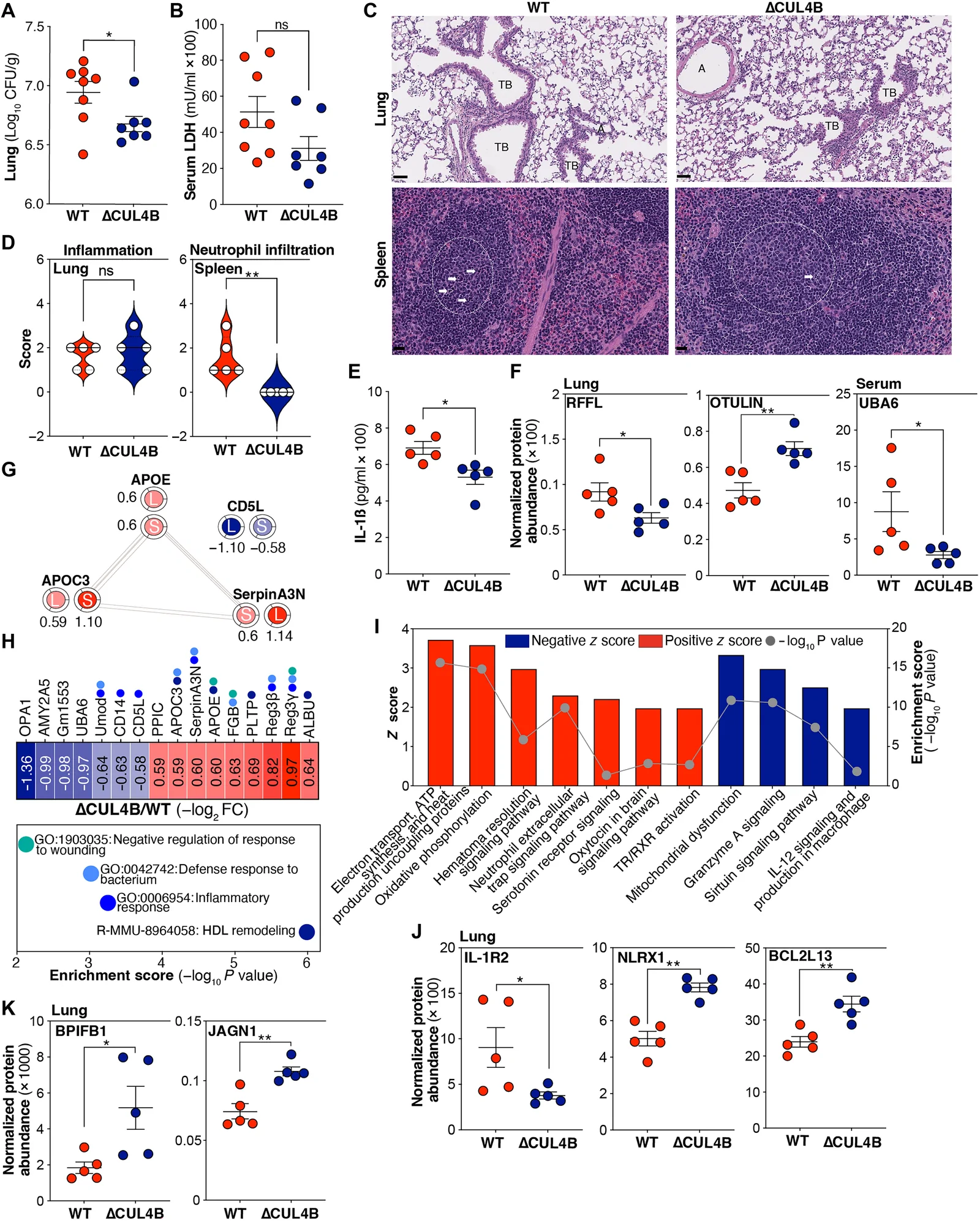
CUL4B deficiency facilitates MRSA clearance in a murine pneumonia model. (**A** and **B**) WT and ΔCUL4B mice were inoculated IT with MRSA. Lung bacterial burden (A) and serum LDH levels (B) were quantified at 24 hpi. Data represent means ± SEM from seven to eight mice per group. (A) *P* = 0.0377. (**C**) Representative H&E-stained 4-μm sections of lung (20×) and spleen (40×) from infected WT and ΔCUL4B mice (*n* = 5 per group), collected 24 hpi and analyzed by light microscopy. Top: TB: terminal bronchiole. A: arteriole. Scale bar, 50 μm. Bottom: Lymphoid follicles with reactive germinal centers (white dashed ovals) and apoptotic lymphocytes (white arrows). Scale bar, 20 μm. (**D**) Histopathological scoring of inflammation and neutrophil infiltration in lung and spleen tissues. Violin plots show interquartile interval (dashed line) and median (solid line). Spleen: *P* = 0.0079. (**E**) IL-1β levels in lung homogenates were quantified. Data represent means ± SEM from five mice per group. *P* = 0.0155. (**F**) Protein abundance of RFFL (*P* = 0.030), OTULIN (*P* = 0.0063), and UBA6 (*P* = 0.031) in lung and serum proteomes. (**G**) STRING analysis of significantly regulated proteins shared between serum (S) and lung (L) proteomes. Node color indicates Log_2_ FC (ΔCUL4B/WT). (**H**) Heatmap and Metascape (*121*) enrichment analysis of regulated serum proteins. Immunoglobulin-associated proteins excluded. (**I**) IPA analysis of altered canonical pathways in lung proteomes (dataset S21). Only infection- and inflammation-related pathways are shown. (**J** and **K**) Protein abundance of IL-1R2 (*P* = 0.037), NLRX (*P* = 0.0033), BCL2L13 (*P* = 0.0031), BPIFB1 (*P* = 0.0146), and JAGN1 (*P* = 0.0059) in the lung proteome. Data were analyzed by two-tailed *t* test (A, B, and E) and the Mann-Whitney test (D). Significance: *P ≤ 0.05; **P ≤ 0.01; ns, *P* > 0.05.

Histopathological analysis of lung tissue [hematoxylin and eosin (H&E) staining] from MRSA-infected WT and ΔCUL4B mice demonstrated mild pulmonary inflammation in both groups, with comparable neutrophil infiltration and tissue damage across lung compartments including parenchyma, air spaces, and perivascular regions (Fig. 4, C and D). However, spleens from WT mice displayed significantly greater neutrophil infiltration compared with ΔCUL4B mice (Fig. 4, C and D) (*P* < 0.05), indicating altered peripheral immune cell accumulation during MRSA pneumonia. Consistent with the reduced inflammasome activity observed in vitro, ΔCUL4B mice displayed significantly lower IL-1β levels in lung homogenates 24 hours after MRSA infection (Fig. 4E). Together, these findings indicate that CUL4B activity is associated with enhanced inflammasome-mediated inflammation and higher levels of bacteremia during MRSA pneumonia, whereas CUL4B deficiency improves bacterial clearance and limits bacterial burden in blood.

### Proteomic insights into CUL4B deletion during MRSA pneumonia

To further characterize the effects of CUL4B deletion during infection, we performed proteomic profiling of lung and serum samples collected 24 hours post–IT MRSA challenge (datasets S18 and S20). Volcano plot analysis identified significant differential regulation of 121 proteins in the lung and 60 proteins in serum of ΔCUL4B- versus WT-infected mice, including several immunoglobulin-related proteins (fig. S13D and datasets S19 and S21). Euclidean hierarchical clustering of normalized protein abundances highlighted distinct proteomic signatures separating the two genotypes (fig. S13E). Notably, loss of CUL4B resulted in altered abundance of several regulators of ubiquitin signaling. The E3 ubiquitin ligase rififylin (RFFL) and the ubiquitin-like modifier-activating enzyme 6 (UBA6), key regulators of protein ubiquitination (*82**–**84*), were significantly reduced in the lung and serum proteomes (Fig. 4F). Conversely, the deubiquitinating enzyme OTULIN (ubiquitin thioesterase otulin), which specifically cleaves linear polyubiquitin chains (*85*, *86*), was significantly increased in the lung proteome of MRSA-infected ΔCUL4B mice (Fig. 4F). Together, these changes suggest that CUL4B deletion perturbs ubiquitin signaling networks during MRSA pneumonia.

Several immune-associated proteins were also differentially regulated in the lung and serum proteomes of MRSA-infected ΔCUL4B mice, including SERPINA3N, CD5L, APOE, and APOC3, along with nine immunoglobulin G–related proteins (Fig. 4G and datasets S19 and S21). Among these, CD5L is noteworthy because elevated CD5L levels have been reported in both experimental pneumonia models and clinical cases of SA/MRSA pneumonia (*87*). In contrast, reduced levels of APOC3 and APOE have been linked to organ dysfunction and mortality in patients with sepsis and septic shock (*88*). Consistent with the improved pulmonary bacterial clearance and reduced bacteremia observed in ΔCUL4B mice (Fig. 4A and fig. S13, B and C), CD5L was reduced (fig. S13F), whereas APOE and APOC3 were increased in the proteomes of MRSA-infected ΔCUL4B animals (Fig. 4G). Together, these proteomic signatures further support a protective host response in the absence of CUL4B during MRSA pneumonia.

### Functional and metabolic impact of CUL4B deletion

Functional enrichment analysis of serum markers (dataset S19) indicated that CUL4B deletion significantly affected pathways related to lipid metabolism, inflammation, and immune responses (Fig. 4H). In particular, the GO term “high-density lipoprotein (HDL) remodeling” was linked to increased abundance of ALBU, PLTP, APOE, and APOC3 (Fig. 4H). Consistent with these findings, serum lipid profiling revealed significantly elevated LDL levels in WT mice (*P* < 0.05), while HDL levels were modestly reduced at 24 hours post–IT infection (fig. S13G). Together, these results indicate altered lipid metabolism in ΔCUL4B mice during MRSA infection. CUL4B-deficient mice also exhibited increased expression of antimicrobial peptides with tissue repair functions (*89**–**91*), such as Reg3γ and Reg3β (Fig. 4H), consistent with a protective host response. In contrast, soluble CD14, a prognostic marker of Gram-positive sepsis (*92*) and an indicator of organ dysfunction in patients with sepsis (*88*), was elevated in WT mice (Fig. 4H and fig. S13H), consistent with the increased bacteremia observed following MRSA infection in these mice (fig. S13, B and C).

Functional exploration of regulated lung proteins (dataset S21) using IPA identified significant activation or inhibition of 11 canonical pathways in ΔCUL4B- versus WT-infected mice (Fig. 4I). Notably, pathways associated with excessive inflammatory responses, including granzyme A signaling and IL-12 production in MΦ, were inhibited in ΔCUL4B mice. In contrast, pathways linked to mitochondrial function and metabolic regulation, such as oxidative phosphorylation and TR/RXR activation, as well as immune modulatory signaling pathways including serotonin receptor signaling, were enhanced (Fig. 4I). In addition, pathways related to neutrophil activity (e.g., NETs signaling) and tissue repair processes (e.g., hematoma resolution) were significantly up-regulated, suggesting improved host defense while limiting inflammatory tissue damage (Fig. 4I).

Several molecular regulators contribute to this balanced immune state (dataset S21). NLR family member X1 (NLRX1), a known negative regulator of inflammatory signaling that helps fine-tune immune responses (*93*), and IL-1 receptor type 2 (IL-1R2), a decoy receptor that limits IL-1β signaling (*94*, *95*), were differentially regulated in ΔCUL4B mice. Suppression of IL-1R2 is noteworthy, as elevated IL-1R2 levels have been associated with sepsis (*96*) and acute respiratory distress syndrome (*97*). In addition, ΔCUL4B mice exhibited increased expression of Bcl-2–like protein 13 (BCL2L13), a BCL2-family protein involved in regulating apoptotic cell death (Fig. 4J) (*98*). Because bacterial pathogens have been shown to suppress BCL2L13 expression as an immune evasion strategy (*99*, *100*), its increased abundance in CUL4B-deficient animals may contribute to improved bacterial clearance. Further supporting enhanced antimicrobial capacity, CUL4B-deficient mice exhibited increased expression of protein jagunal homolog 1 (JAGN1) (Fig. 4K), a regulator of neutrophil antimicrobial function (*101*), and BPI fold-containing family B member 1 (BPIFB1) (Fig. 4K), a protein with direct antimicrobial activity (*102*). However, multiple neutrophil serine proteases, including CTSG, ELANE, and PRTN3, which constitute the nonoxidative antimicrobial arsenal were also down-regulated (fig. S13I), suggesting remodeling of neutrophil antimicrobial effector program.

Together, these findings indicate that CUL4B deletion promotes a coordinated host response characterized by enhanced antimicrobial defenses, improved metabolic adaptation, and reduced immune-mediated tissue damage, thereby contributing to improved outcomes during MRSA pneumonia. These proteomic and pathway-level changes are consistent with the improved bacterial clearance and reduced systemic dissemination observed in ΔCUL4B mice, suggesting that CUL4B activity shapes host ubiquitin signaling networks that influence immune and metabolic responses during infection.

## DISCUSSION

MΦ are crucial effector cells in controlling bacterial burden and mitigating SA-associated lethality, making them central to host defense across diverse in vivo infection models (*35**–**37*, *103*, *104*). To advance the development of host-directed therapeutics, recent studies have applied multi-omics approaches to generate high-resolution insights into the complex molecular networks governing SA-MΦ interactions (*105*, *106*). Here, we combined high-throughput experimentation with multi-omics analysis to define MΦ-specific signatures associated with MRSA infection across multiple regulatory layers. The limited concordance between transcriptomic and proteomic datasets likely reflects extensive posttranscriptional and translational regulations during the MΦ response to MRSA infection. While several canonical inflammatory pathways (e.g., IFN signaling) were recapitulated, our study identifies the E3 ubiquitin ligase CUL4B as a candidate regulator of MΦ innate immune responses to MRSA infection. Iterative validation of multi-omics predictions through functional assays further illustrates the utility of systems-level approaches in identifying host-directed regulators of bacterial infections. Future studies using dual host-pathogen multi-omics approaches will further refine this regulatory landscape by capturing reciprocal adaptations during MRSA infection.

Our findings suggest that loss of CUL4B is associated with a shift in MΦ antimicrobial properties, characterized by enhanced MRSA killing alongside selective changes in effector molecules such as CTSL and S100A8/A9. While these molecular changes are associated with the observed functional phenotype, the current data do not establish whether they are causally responsible for increased bactericidal activity. Rather, they likely reflect components of a broader antimicrobial state associated with loss of CUL4B. Dissecting which downstream pathways are necessary or sufficient for this phenotype will require further mechanistic mapping of CUL4B-dependent substrates and regulatory networks.

Inflammasome activation represents a double-edged sword in the host response to bacteria, depending on the site, stage, and magnitude of infection (*107**–**109*). During early and/or low-inoculum stages of infection, inflammasome-induced IL-1β plays a key role in recruiting immune cells and promoting bacterial clearance before systemic spread. In contrast, during deep-seated or systemic infections, excessive inflammasome activation, MΦ cell death, and exaggerated IL-1β release can drive hyperinflammatory, neutrophil-mediated tissue damage and contribute to septic shock pathophysiology. For example, while NLRP3 deletion (*34*) or pharmacological inhibition (*27*) protects mice against lethality in hyperinflammation-driven SA pneumonia, it does not confer protection in nonlethal, mucosal SA urinary tract infection (*110*). In this context, CUL4B appears to influence the threshold of inflammasome activation in MΦ. We find that CUL4B deficiency reduces caspase-1 activation, limits IL-1β maturation, and diminishes pyroptotic cell death during MRSA infection. Pharmacological inhibition of NLRP3 or caspase-1 reproduced the enhanced bactericidal phenotype observed in ΔCUL4B MΦ, whereas experimental activation of the inflammasome partially reversed this phenotype. Together, these complementary perturbation studies support a functional contribution of inflammasome signaling to the antibacterial effects associated with CUL4B deficiency. Thus, while additional mechanisms are likely to contribute, restrained inflammasome activation represents the most functionally supported pathway linking CUL4B deficiency to improved MΦ control of intracellular MRSA. In addition, the requirement for TLR2 signaling in the enhanced antibacterial phenotype of ΔCUL4B MΦ suggests that the protective effects of CUL4B deficiency occur within the context of intact TLR2-dependent antibacterial responses. The precise relationship between TLR2 signaling and altered inflammasome activity in CUL4B-deficient MΦ remains an important area for future investigation.

Our studies identify CUL4B as a contributor to inflammasome-associated inflammatory responses during MRSA pneumonia. Consequently, *CUL4B* deletion or broad-spectrum CRL inhibition improves bacterial clearance and reduces lung levels of IL-1β, a key inflammasome-dependent cytokine. Although pulmonary MRSA reduction in ΔCUL4B mice is modest, it was accompanied by reduced bacteremia, indicating that even subtle improvements in local bacterial control can affect systemic infection burden. Because these analyses were performed using global knockout mice, contributions from other immune populations remain possible and will require future cell type–specific studies. CUL4B deficiency was also associated with enhanced intracellular MRSA killing, reduced inflammasome activity, and decreased pyroptosis in MΦ. In line with our findings, inhibition of NLRP3 inflammasome activation has previously been shown to enhance SA clearance by both primary human monocytes and murine MΦ (*27*). Notably, individuals with *CUL4B* mutations at conserved amino acid residues exhibit elevated peripheral monocyte counts, further supporting a regulatory role for CUL4B in monocyte and MΦ biology (*44*). The augmented bactericidal capacity of CUL4B-deficient MΦ was notably specific to MRSA. Elucidating whether discrete SA virulence determinants selectively interface with CUL4B-dependent pathways during intracellular infection will be an important focus of future investigations.

While loss of CUL4B led to reduced IL-1β levels in MΦ, prior work has shown that stable CUL4B knockdown by short hairpin RNA decreases LPS-induced tristetraprolin (TTP), a key regulator of cytokine mRNA stability, thereby attenuating TNF production in RAW264.7 murine MΦ (*56*). TTP also modulates the stability of additional inflammatory mediators, including granulocyte-macrophage colony-stimulating factor, IL-6, CCL3, and IL-1β (*111**–**114*). *CUL4B*-deficient BMDMs exhibited altered TNF-α and IL-6 production in response to stimulation with LPS or other TLR ligands (*39*, *53*), potentially through aberrant TTP regulation. Thus, CUL4B’s modulation of MΦ inflammatory responses is likely context-dependent, with its net effect influenced by the relative contribution of inflammasome signaling. Although CUL4B is established as a scaffold for cullin-RING E3 ligases, the specific MΦ substrates orchestrating MRSA-selective responses remain undefined. Our proteomics analyses implicate alterations in multiple protein networks, including lysosomal proteases, providing a framework for future mechanistic investigation.

Given the critical role of NLRP3-mediated inflammasome activation in host defense against pathogenic bacteria, including MRSA [reviewed in (*115*)], it is important to systematically identify additional host regulators of inflammasome dynamics. Key cellular processes—such as membrane permeabilization, lysosomal destabilization, mitochondrial dysfunction, and post-translational modifications (PTMs) of NLRP3 and associated inflammasome components—serve as major triggers of activation (*116**–**118*). Notably, ubiquitination of NLRP3 by specific E3 ubiquitin ligases can either enhance or suppress inflammasome activation [reviewed in (*118*)]. For example, Pellino-2 promotes K63-linked ubiquitination that enhances NLRP3 activation and inflammasome assembly, leading to IL-1β maturation and secretion (*66*). Loss of Pellino-2 impairs NLRP3 inflammasome activation, reduces LPS-induced mortality in vivo, and decreases IL-1β secretion in MΦ exposed to *Pseudomonas aeruginosa* and *Escherichia coli* (*66*).

Collectively, our data suggest that CUL4B positively influences NLRP3 inflammasome activation. CUL4B deficiency is associated with reduced NLRP3 abundance, diminished caspase-1 activation, decreased IL-1β production, and reduced pyroptotic cell death during MRSA infection. Pharmacological inhibition of NLRP3 or caspase-1 recapitulated the enhanced bactericidal phenotype observed in ΔCUL4B MΦ, supporting a functional contribution of inflammasome signaling to the antibacterial effects associated with CUL4B deficiency. However, the direct molecular substrates and regulatory mechanisms linking CUL4B activity to altered inflammasome responses remain incompletely defined.

At the same time, the proteomics analyses provide additional insight into molecular networks that may shape this phenotype and warrant further investigation. Among the most prominent changes were alterations in proteins involved in ubiquitin-dependent signaling, including reduced abundance of the ubiquitin ligase RFFL and the ubiquitin-activating enzyme UBA6, together with increased abundance of the deubiquitinase OTULIN. Given the established role of ubiquitin-dependent regulation in inflammatory signaling and NLRP3 inflammasome activity, these observations are consistent with a broader shift in the regulatory landscape governing innate immune activation. In particular, OTULIN has been shown to restrict linear ubiquitin chain accumulation and dampen nuclear factor κB signaling (*119*), suggesting one potential mechanism through which inflammatory responses may be restrained in the absence of CUL4B. Additional changes were observed in pathways linked to cellular homeostasis and host defense. Increased abundance of BCL2L13, a regulator of mitochondrial and apoptosis-associated pathways (*98*), points to potential alterations in stress-response pathways during infection. Likewise, changes in JAGN1 and BPIFB1, together with alterations in neutrophil serine proteases and pathways associated with granzyme A signaling and neutrophil extracellular trap (NET) formation, suggest broader remodeling of antimicrobial effector programs within the infected host. Although these observations provide potentially informative clues regarding downstream consequences of CUL4B deficiency, the present study was not designed to determine the functional contribution of these individual factors. Accordingly, these proteomic signatures should be viewed as a framework for future mechanistic studies aimed at identifying the specific CUL4B-dependent substrates and regulatory networks that govern MΦ responses to MRSA infection.

Together, these changes place CUL4B upstream of a coordinated regulatory axis linking ubiquitin signaling and cell fate decision to inflammatory outcome of MRSA infection. Although further studies are required to elucidate the precise molecular mechanisms involved, small molecule inhibitors targeting the CRL4 complex in which CUL4B serves as a scaffold, have demonstrated antitumor activity in preclinical models (*120*). Similar strategies may hold promise as host-directed therapeutics to limit hyperinflammation during severe bacterial infections.

## MATERIALS AND METHODS

Detailed materials and methods describing bacterial strains, cell lines, stimulation practices, bacterial internalization and cytotoxicity assay, proteomic and transcriptomic profiling of MRSA-infected MΦ and data analysis, immunoblot analysis, qRT-PCR analysis, IFNAR signaling assay, confocal microscopy analysis, structural analysis, real-time cytotoxicity analysis using Incucyte, caspase-1 activation analysis, neutrophil isolation and serum collection, neutrophil killing assay, murine model of IT MRSA infection, lung and spleen histopathology, cytokine and cholesterol levels analysis, proteome analysis of murine lung and serum, and statistical analysis are provided in the Supplementary Materials.

### Ethics statements

All animal experiments were conducted in compliance with ethical guidelines for animal research, following Institutional Animal Care and Use Committee regulations and approved under UC San Diego IRB protocol S00227M and Weill Cornell Medicine IRB 2009-0110. Blood samples from healthy volunteers were collected with written informed consent, adhering to the ethical principles of the Declaration of Helsinki and approved by the UC San Diego Human Research Protection Program (IRB protocol 131002).

## Supplementary Materials

### The PDF file includes:

Supplementary Results Supplementary Materials and Methods Figs. S1 to S16 Table S1 Legends for datasets S1 to S21 References

### Other Supplementary Material for this manuscript includes the following:

Datasets S1 to S21
