# Supplementary material for: Integrative multi-omics reveals CUL4B as a modulator of macrophage pyroptosis and innate defense against Staphylococcus aureus
Source: Sci Adv. 2026 Oct 14;12(42):eaeh4023. doi: 10.1126/sciadv.aeh4023 (PMC13665969; doi:10.1126/sciadv.aeh4023)
Supplement: Supplementary file 1 [file sciadv.aeh4023_sm.pdf]

Science Advances
AAAS

Supplementary Materials for

Integrative multi-omics reveals CUL4B as a modulator of macrophage pyroptosis and innate defense against *Staphylococcus aureus*

Fatemeh Askarian *et al.*

Corresponding author: Fatemeh Askarian, faaskarian@health.ucsd.edu; Victor Nizet, vnizet@health.ucsd.edu

Sci. Adv. **12**, eaeh4023 (2026)
DOI: 10.1126/sciadv.aeh4023

The PDF file includes:

Supplementary Results
Supplementary Materials and Methods
Figs. S1 to S16
Table S1
Legends for datasets S1 to S21
References

Other Supplementary Material for this manuscript includes the following:

Datasets S1 to S21

SUPPLEMENTARY RESULTS

Validation of multi-omics datasets characterizing regulatory networks in the MΦ response to intracellular MRSA infection. MRSA intracellular viability analysis revealed more than 90% of MRSA were either phagocytosed by or adhered to MΦ within one hour post-infection (hpi), prior to antibiotic (ABX, gentamicin/lysostaphin) treatment to eliminate extracellular or escaped bacteria. Intracellular MRSA survival progressively declined at 2, 4, 7, and 24 hpi **(fig. S1A)**. By 2 hpi (1 h post-ABX treatment), lactate dehydrogenase (LDH) release, a marker of host cell lysis, increased, suggesting that the reduction in intracellular MRSA was due to both MΦ-mediated killing and bacterial escape into the extracellular milieu **(fig. S1B)**.

Principal component analysis (PCA) (**Figs. 1A–B**) was further supported by hierarchical clustering of π-scores **(fig. S1C)** and abundance heatmaps **(fig. S1D)** from the proteome analyses. Given the similarity in translational responses at 4 and 7 hpi **(Fig. 1A, fig. S1D)**, these time points were pooled for subsequent analysis **(fig. S1C)**. Volcano plots **(fig. S1E)** and Venn diagrams of significantly regulated proteins **(fig. S1F)** and genes **(fig. S4A)** revealed differential expression across all examined time points **(Datasets S2, S5)**. Some changes were time point-specific, whereas others were conserved across multiple time points **(Datasets S3, S6; figs. S1F and S4A)**.

Functional enrichment analysis via Ingenuity Pathway Analysis (IPA) **(fig. S2)** and Gene Ontology (GO) **(figs. S3A–D, S4B–D)** provided insight into key MΦ response pathways, including inflammatory and oxidative stress responses. GO enrichment analysis of significantly regulated proteins **(figs. S3A–C)** and genes **(figs. S4B–D)** highlighted shared terms related to antiviral defense responses and type interferon (IFN) signaling, consistent with prior studies linking type I interferon (IFN) signaling to SA pathogenesis and immune modulation (*67–70*). To validate the reproducibility of our datasets, we evaluated IFN regulatory factor (IRF) pathway activation in response to internalized MRSA and an additional MRSA USA300 strain (LAC) using WT and ΔIFNAR2 reporter THP-1 cells **(fig. S5A)**. While no luciferase activity was detected in ΔIFNAR2 MΦ, both MRSA strains significantly induced luciferase expression in WT cells compared to mock-infected controls **(fig. S5A)**. This was accompanied by increased production of several inflammatory cytokines (e.g., TNF, IL-1β, IP-10) in MRSA-infected WT MΦ cells compared to ΔIFNAR2 **(fig. S5B)**. Confocal microscopy confirmed colocalization of GFP-expressing MRSA with pSTAT1, indicating activation of IFN-I signaling **(fig. S5C)**. These findings confirm the reproducibility of the multi-omics datasets and demonstrate activation of IFN-I signaling during intracellular MRSA infection.

Integrated transcriptomic and proteomic analyses identify multilayered CUL4B-associated regulatory programs. Comparative transcriptomic and proteomic analyses revealed both concordant and discordant regulatory patterns among CUL4B-associated targets (**Fig. 1F**, **fig. S7B).** Non-differentially expressed genes and proteins are shown in *italics* in the figures.

Of the 79 significantly regulated proteins and genes associated with CUL4B, twenty-three targets were shared across all identified regulatory networks (**Fig. 1F**). Six proteins/genes (AKT1, SAE1, EIF4G1, MAT2A, UBE2I, GLS) exhibited differential regulation at the protein level without corresponding changes at the mRNA level. In contrast, 17 genes exhibited concordant regulation at both transcript and protein levels **(Fig. 1F)**. An additional five genes (*EIF4E, IFIT2, PLAU, MX*2, and *SP100*) showed consistent regulation in only one molecular layer, consistent with partial coupling between transcript and protein dynamics **(Fig. 1F)**.

Beyond these shared targets, 56 genes/proteins were unique to this regulator **(fig. S7B; Dataset S10)**. Within this subset, five proteins/genes (PRDX3, TPM3, TPI1, NDUFB1, HSPA9) were specifically linked to CUL4B across proteomic and network-based analyses **(Dataset S8)**. Notably, a larger group of 31 proteins, including GLO1, PREP, SOD1, SUMO2/3, BAX, EZH2, RHOA, and NPM1, were differentially expressed at the protein level but not at the mRNA level **(fig. S9B;**

Dataset S10). Finally, 27 genes showed distinct regulation at the mRNA level in MRSA-infected MΦ compared with mock-infected cells.

SUPPLEMENTARY MATERIALS AND METHODS

Bacterial strains and growth conditions. *Staphylococcus aureus* (SA) strains used included USA300-MRSA (TCH1516, ATCC BAA-1717, WT MRSA), its isogenic mutant USA300-MRSAΔHla (ΔHla) (*123*), and MRSA LAC (USA300-0114 PFGE type) (*124*) (gift from Prof. Alex Horswill, University of Colorado). Additional strains included *Streptococcus pyogenes* (M1T1, strain 5448 (*125*)), and *S. agalactiae (*COH1, serotype III, MLST-17) (*126*). Clinical MRSA isolates from patients with ankle arthritis/hardware (02/2023) and pacemaker endocarditis (01/2023) were provided by Dr. George Sakoulas (UC San Diego). SA cultured Todd Hewitt broth (THB) at 37°C with shaking (200 rpm). *S. pyogenes* and *S. agalactiae* were grown in THB at 37°C under static conditions. For infections, overnight cultures were washed twice in 1× PBS and resuspended in RPMI/FBS (see below) or 1× PBS (Sigma-Aldrich).

Cell culture and genetic modifications. THP-1 monocytes (ATCC TIB-202, *Homo sapiens*, male) were authenticated by ATCC via short tandem repeat (STR) profiling. Cells were maintained in RPMI 1640 (Gibco) supplemented with 10% fetal bovine serum (FBS, Avantor Seradigm), 2 mM L-glutamine, 4.5 g/L glucose, 10 mM HEPES, 1 mM sodium pyruvate (Gibco), 0.05 mM 2-mercaptoethanol (Sigma-Aldrich), and 100 U/mL penicillin–100 µg/mL streptomycin (Gibco) at 37°C with 5% CO_2. For infection assays, penicillin/streptomycin were omitted, and this medium is referred to as RPMI/FBS. THP-1 KO-CUL4B (ΔCUL4B) cells (transcript ID: ENST00000371322.11) were generated by Synthego via CRISPR/Cas9 targeting CUL4B exon 5 (gRNA: UGGCAUCCUUACCAAAUGGA, PAM: GGG). Editing efficiency in pooled knockouts was estimated at 94%. Single-cell clones were Sanger sequenced using primers CUL4BFW (5′-AGTCCCCTCGGCTAACAAAA-3′) and CUL4BREV (5′-TGGGCATATTCTCAGATAGATGTGT-3′), aligned to WT THP-1, and confirmed via immunoblot and RT-PCR (outlined in the “Immunoblot analysis” and “Quantitative real-time polymerase chain reaction (qRT-PCR) analysis” sections). THP-1 Dual (thpd-nfis) and THP-1 Dual KO-IFNAR2 (ΔIFNAR2) (thpd-koifnar2) cells were obtained from InvivoGen and maintained per the manufacturer’s instructions in RPMI 1640 supplemented with 10% FBS, 2 mM L-glutamine, 10 mM HEPES, 100 µg/mL normocin (InvivoGen), and 100 U/mL penicillin–100 µg/mL streptomycin. When required, blasticidin and zeocin (InvivoGen) were added. THP-1 cells were cultured at 5×10^5/mL, passaged every 4–5 days, and routinely tested for Mycoplasma contamination using MycoStrip (InvivoGen). Bone marrow-derived macrophages (BMDMs, *mus musculus*, C57BL/6 background) were isolated from femur and tibia from both hind limbs of *CUL4B* knockout and WT mice (see “murine model of intratracheal (IT) MRSA infection” for details). The cells were cultured in DMEM/F12 (Sigma-Aldrich) supplemented with FBS (10% v/v), 100 U/mL penicillin–100 µg/mL streptomycin, and recombinant mouse-derived macrophage colony-stimulating factor (M-CSF, 25 ng/mL, Proteintech). The culture medium was replaced every other day. On day 6, differentiated BMDMs were seeded into 24-well plates at a density of 5×10^5 cells/well and used for the MRSA challenge the following day.

Bacterial internalization and cytotoxicity assay. THP-1 cells were seeded in 12-well plates (1×10^6 cells/well) in RPMI/FBS and differentiated with 25 nM phorbol 12-myristate 13-acetate (PMA, Sigma-Aldrich). After 24 hours, cells were washed and replenished with fresh RPMI/FBS. Bacterial cultures were prepared as described in “Bacterial strains and growth conditions,” washed with PBS, and resuspended in RPMI/FBS at the desired CFU. Differentiated THP-1 cells (48 h) or BMDMs (day 7) were infected with MRSA at the indicated MOI for 1 h at 37°C with 5% CO_2. Uninfected cells (mock) served as controls. Cells were then washed with PBS, and extracellular MRSA was efficiently eliminated by replacing the medium with RPMI/FBS containing 40 µg/mL lysostaphin from *Staphylococcus staphylolyticus* (Sigma-Aldrich) and 100 µg/mL gentamicin (Sigma-Aldrich) (ABX) (*127*, *128*). Mock-infected cells were subjected to the same antibiotic treatment conditions (e.g., gentamicin/lysostaphin) to control for potential effects of the antibiotic exposure on macrophage responses. When relevant, infections were also performed with *S. pyogenes* (MOI = 5) and *S. agalactiae* (MOI = 5) with extracellular bacteria removed using gentamicin/penicillin G

(Sigma-Aldrich). At the indicated time points, cells were washed, lysed with ice-cold H_2O containing 0.3% saponin for 5 minutes at 4°C, and pipetted vigorously. Bacterial survival was assessed by serial dilution, plating on Todd Hewitt agar (THA), overnight incubation at 37°C, and colony counting.

When indicated, cells were pretreated with vehicle, MCC950 (InvivoGen, inh-mcc), MLN4924 (MedChemExpress, HY-70062), bafilomycin A1 (InvivoGen, tlrl-baf1, 1 µM), VX-765 (InvivoGen, inh-vx765i-1, 50 µM), and TL2-C29 (InvivoGen, inh-C29, 200 µM) for 90 minutes before infection. When appropriate, THP-1 cells were primed with LPS (1 µg/mL, Sigma-Aldrich) for 3 h, followed by ATP treatment (50 µM, InvivoGen) for 30 minutes, after which the medium was replaced prior to MRSA infection. Supernatants were collected and analyzed for cytotoxicity using a cytotoxicity detection kit or an LDH-Glo cytotoxicity kit (Promega) per the manufacturer's instructions. MOI, inhibitor concentrations, and time points for plating are detailed in figure legends.

Proteomic profiling of MRSA-infected macrophages and data analysis. THP-1 cells (ATCC) were seeded in 175 cm² flasks (30 × 10^6 cells/flask, Corning) and differentiated with 25 nM PMA (Sigma-Aldrich). After 24 h, cells were washed and replenished with fresh RPMI/FBS. Forty-eight hours post-differentiation, cells were infected with MRSA (MOI = 5) for 1 hour at 37°C with 5% CO_2. Uninfected cells (mock) served as controls. Cells were then washed with PBS, and extracellular bacteria were eliminated by replacing the medium with RPMI/FBS containing 40 µg/mL lysostaphin and 100 µg/mL gentamicin. Each experimental time point used 1.2 × 10^8 cells per biological replicate. At 1, 2, 4, 7, and 24 hours post-infection (hpi), cells were harvested and lysed in a buffer containing 3% SDS (Fisher Scientific), 75 mM NaCl, 1 mM NaF, 1 mM β-glycerophosphate, 1 mM sodium orthovanadate, 10 mM sodium pyrophosphate, 1 mM PMSF, 1× Complete Mini EDTA-free protease inhibitor (Roche), and phosphatase inhibitor (Thermo Fisher Scientific) in 50 mM HEPES (pH 8.5) (*129, 130*). Lysates were vortexed, sonicated for 10 minutes, and centrifuged (13,200 rpm, 5 min). Supernatants were collected for downstream analysis, as previously described with modifications (*130*).

Following lysis, proteins were reduced and alkylated with dithiothreitol (DTT) and iodoacetamide (IAA, Sigma-Aldrich) as previously described (*131*). Proteins were precipitated using the chloroform/methanol method (*132*), and pellets were resuspended in 1 M urea, 50 mM HEPES (pH 8.5) for digestion. Proteins were digested as previously described (*130*), and peptides were desalted with C18 solid-phase extraction columns (Waters) (*133*). Desalted peptides were dried, resuspended in 50% acetonitrile/5% formic acid, and quantified using the Pierce Quantitative Colorimetric Peptide Assay (Thermo Fisher Scientific). Next, 50 µg of peptide per sample were labeled, and an equal amount from each sample was pooled to create a standard bridge channel for each MS run (*134*). Peptides were labeled with tandem mass tag (TMT) reagents (Thermo Fisher Scientific) (*135*, *136*). TMT reagents were prepared at 20 µg/mL in dry acetonitrile and incubated with peptides resuspended in 30% acetonitrile, 200 mM HEPES (pH 8.5). Each sample was randomized and labeled with 8 µL of its respective TMT reagent, with TMT-126 used for the pooled standard bridge channel. Labeling proceeded for 1 h at room temperature before quenching with 9 µL of 5% hydroxylamine (Sigma-Aldrich) for 15 minutes. Labeled samples were acidified with 50 µL of 1% trifluoroacetic acid, combined into their respective multiplexed experiments, desalted, and lyophilized. Multiplexed samples were fractionated using the Pierce High pH Reversed-Phase Peptide Fractionation Kit (Thermo Fisher Scientific) per the manufacturer's instructions. Fractionated peptides were lyophilized and resuspended in 5% acetonitrile/5% formic acid for LC-MS^2/MS^3 analysis.

LC-MS^2/MS^3 analysis was performed on an Orbitrap Fusion mass spectrometer (Thermo Fisher Scientific) coupled to an Easy-nLC 1000 system. Peptides were separated on a home-packed column (100 µm ID, 360 µm OD) containing 0.5 cm C4 resin (5 µm), 0.5 cm C18 resin (3 µm), and 30 cm C18 resin (1.8 µm) at 60°C. Elution was performed with a linear gradient of 11–30% acetonitrile in 0.125% formic acid at 300 nL/min over 165 minutes. Electrospray ionization was

applied at 2000 V via a stainless-steel T-junction. Mass spectrometry was conducted in data-dependent mode with an MS1 survey scan (m/z 500–1200) at 120,000 resolution in the Orbitrap (AGC: 5×10^5, max injection time: 100 ms, s-lens RF: 60). Top-speed mode was used to maximize spectra acquisition within a 5 s cycle. The most abundant ions were selected for MS^2 and MS^3 analysis for peptide identification and quantitation, respectively. For MS^2, ions above 5×10^5 intensity were isolated (0.5 m/z window), fragmented by collision-induced dissociation (NCE: 30%), and detected in the ion trap (rapid scan rate, AGC: 1×10^4, max injection time: 35 ms). MS^3 analysis used synchronous precursor selection to enhance TMT reporter ion quantification (*137*). A maximum of 10 MS^2 ions were isolated (2 m/z window) and fragmented via higher-energy collision dissociation (NCE: 55%). MS^3 fragment ions were detected in the Orbitrap at 60,000 resolution (low mass: 110 m/z, AGC: 1×10^5, max injection time: 100 ms). MS^2 ions within 40 m/z below and 15 m/z above the precursor were excluded from MS^3 isolation.

MS raw files were processed using Proteome Discoverer 2.1.0.81 (Thermo Fisher Scientific). Peptide-spectrum matching was performed with the SEQUEST-HT node (*139*) against a concatenated *Homo sapiens* and *S. aureus* USA300-MRSA database (UniProt, downloaded 08/03/2016). A decoy search with reversed protein sequences estimated the false discovery rate (FDR) (*139–141*). Search parameters included a 50 ppm MS^1 mass tolerance (*142*, *143*), 0.6 Da MS^2 mass tolerance, and full trypsin digestion with up to two missed cleavages. Static modifications included TMT-10plex labeling (lysines/N-termini) and carbamidomethylation (cysteines); variable modifications included methionine oxidation and serine, threonine, and tyrosine phosphorylation. Peptide and protein identifications were filtered to <1% FDR using a target-decoy approach (*141*). Ambiguous peptides were assigned to the protein with the most matched peptides per the law of parsimony (*142*). For quantification, TMT reporter ion intensities were extracted from MS^3 spectra, filtering for spectra with >10 average signal-to-noise ratio per reporter ion and <25% isolation interference (*144*). Data normalization followed a multistep process. At the protein level, reporter ion intensities were summed, normalized to their bridge channel, and then to the bridge channel median (*134*). Next, all values were normalized to the dataset median (*145*).

Dysregulated proteins were identified using one-way ANOVA for mock- and MRSA-infected THP-1 cells (1, 2, 4, 7, and 24 hpi), with significance criteria of fold change (FC) ≥ 1.5 ($\log_2$ FC ≥ 0.58) or FC ≤ −1.5 ($\log_2$ FC ≤ −0.58) and an adjusted *P*-value ≤ 0.05. Volcano plots were generated in Python, and data were transformed using principal component analysis (PCA). K-means clustering identified four groups of time points with distinct protein abundance patterns (1, 2, 4 & 7, and 24 hpi). Dysregulated proteins for each cluster, relative to uninfected controls, were identified using a *t*-test (FC ≥ 1.5 or FC ≤ −1.5, adjusted *P*-value ≤ 0.05) and used as inputs for Ingenuity Pathway Analysis (IPA) (QIAGEN Inc.). Unique and shared dysregulated proteins across clusters were analyzed for pathway enrichment. GO Biological Processes enrichment was visualized using REVIGO (*147*). Upstream regulator analysis in IPA identified activated and inhibited regulators, while causal network analysis defined regulatory pathways (*147*, *148*). Master regulators shared across time clusters (AREG, ATM, CUL4B, MYC, PKG, ROCK, and RACK) with a z-score ≥ 2 or ≤ −2 (adjusted *P*-value < 0.05) were selected for visualization. Unique and shared genes regulated by these master regulators were visualized using Cytoscape (*149*) and analyzed by the UpSetPlot (*150*). CUL4B-regulated gene expression and protein abundance were represented using heatmaps (Seaborn) (*151*) and network graphs (Cytoscape) (*149*). Protein–protein interaction networks were visualized using STRING (version 12) (*152*), and enrichment analysis was performed with Metascape (v3.5.20240901) using a minimum overlap of 3, *P*-value cut-off of 0.01, and a minimum enrichment threshold of 2 (*121*). Metascape *P*-values were calculated using a cumulative hypergeometric distribution. When indicated, pathway analysis was performed using IPA. Pathway significance was determined using a right-tailed Fisher's exact test (*P*-value < 0.05), and pathway activation (≥ 2)/inhibition (≤ −2) was predicted based on the IPA activation z-score.

Transcriptomic profiling of MRSA-infected macrophages and data analysis. THP-1 cells were seeded in 6-well plates (2×10^6 cells/well) in RPMI/FBS and differentiated with 25 nM PMA. After

24 hours, cells were washed and replenished with fresh RPMI/FBS. Forty-eight hours post-differentiation, cells were infected with MRSA (MOI = 5) for 1 h at 37°C with 5% CO_2. Uninfected cells (mock) served as controls. Cells were then washed with PBS, and extracellular bacteria were eliminated by replacing the medium with RPMI/FBS containing 40 μg/mL lysostaphin and 100 μg/mL gentamicin (MRSA).

At 7 and 24 h post-infection (hpi), cells were harvested using RNAprotect (Qiagen), and RNA was extracted with the RNeasy Midi Kit (Qiagen) following the manufacturer's instructions. RNA quality was assessed using an Agilent TapeStation 4200, and samples with an RNA Integrity Number (RIN) >8.0 were used for RNA sequencing library preparation with the TruSeq Stranded mRNA Sample Prep Kit (Illumina). Libraries were generated from 200 ng of RNA per sample, with RNA shear time modified to five minutes. Samples were multiplexed and sequenced with 100-base-pair (bp) paired-end reads (PE100) to a depth of approximately 40 million reads per sample on an Illumina NovaSeq 6000. Demultiplexing was performed using bcl2fastq v2.20 (Illumina). Sequence data were quality-controlled with FastQC and summarized using MultiQC (*153*). Low-quality bases and sequencing adapters were trimmed with Trimmomatic (*154*). Reads were aligned to the GRCh38 GCF_000001405.26 reference genome using STAR (*155*) and quantified with HTSeq (*156*). Principal component analysis of variance-stabilizing transformed counts was performed in R and visualized with ggplot2 (*157*). Differentially expressed genes (DEGs) were identified using DESeq2 (*157*), with a significance threshold of FC ≥ 2 ($\log_2$ FC ≥ 1) or FC ≤ −2 ($\log_2$ FC ≤ −1) and an adjusted *P*-value ≤ 0.05 for MRSA-infected macrophages relative to mock controls at each time point (7 and 24 hpi). Gene set enrichment analysis (GSEA) was performed for genes ranked by shrunken log fold change from DESeq2 shrinkage, and GO Biological Processes enrichment analysis was conducted using ClusterProfiler (*158*).

Immunoblot analysis. Immunoblot analysis was performed on THP-1-derived macrophage lysates as previously described (*159*), with modifications. WT and ΔCUL4B THP-1 cells were seeded in 12-well plates (1 × 10^6 cells/well) in RPMI/FBS and differentiated with 25 nM PMA. Forty-eight hours post-differentiation, cells were either left uninfected or infected with MRSA as described in the "Bacterial internalization and cytotoxicity assay". For CUL4B detection, the cells were washed with PBS and lysed with ice-cold RIPA buffer (Thermo Fisher Scientific) supplemented with 1× Complete Mini EDTA-free protease inhibitor and phosphatase inhibitor. For caspase-1 detection, proteins from both the cells and culture supernatant were precipitated using the methanol/chloroform method. Samples were separated by SDS-PAGE and transferred onto polyvinylidene fluoride (PVDF) membranes (Millipore) using wet transfer. Membranes were blocked with Intercept (PBS) blocking buffer (LI-COR Biosciences) and incubated overnight at 4°C with CUL4B (Proteintech, 12916-1-AP, 1:1000, PRID: AB_2086699), caspase-1 (cleaved Asp297, p20) (Thermo Fisher Scientific, PA5-77886, 1:500, PRID: AB_2735573), caspase-1 polyclonal (to detect procaspase-1, Proteintech, 22915-AP, 1:1000, PRID: AB_2876874), and GAPDH (Thermo Fisher Scientific, PA1-987, 1:1000, PRID: AB_21073711; R&D, MAB5718, Clone #686613, 1:500) antibodies. After four washes, blots were probed with IRDye 800CW goat anti-rabbit secondary antibody (LI-COR Biosciences, 926-32211, 1:10,000), IRDye 680RD goat anti-rabbit secondary antibody (LI-COR Biosciences, 926-68071, 1:10,000), and IRDye 680RD goat anti-mouse secondary antibody (LI-COR Biosciences, 926-68070, 1:10,000). When indicated, the membranes were stripped with NewBlot PVDF stripping buffer (LI-COR Biosciences) according to the manufacturer's instructions, blocked with blocking buffer, and re-probed with the specified primary antibody. Fluorescent blot imaging was performed using the LI-COR Odyssey infrared imaging system (LI-COR Biosciences). MagicMark™ XP (Invitrogen) and/or SeeBlue™ (Invitrogen) protein standards were used as molecular weight markers. When indicated, the ratio of cleaved caspase-1 (p20) to GAPDH was quantified by densitometric analysis using Image Studio. The uncropped blots are provided in figs. S15 and S16.

Quantitative real-time polymerase chain reaction (qRT-PCR) analysis. WT and ΔCUL4B THP-1 cells were seeded in 12-well plates (1 × 10^6 cells/well) in RPMI/FBS, differentiated with PMA,

and either left uninfected or infected with MRSA (MOI = 10) as described in "Bacterial internalization and cytotoxicity assay". At 1 and 3 h post-infection (hpi), cells were treated with RNAlater (Qiagen), and RNA was extracted using the RNeasy Mini Kit (Qiagen) following the manufacturer's instructions. RNA integrity and quantity were assessed using a NanoDrop. Reverse transcription of RNA was performed using the iScript™ Reverse Transcription Supermix (Bio-Rad) per the manufacturer's recommendations. qRT-PCR was conducted using SYBR Green (Bio-Rad) on a CFX96 Touch Real-Time PCR Detection System (Bio-Rad). Gene expression was normalized to *GAPDH*, and relative expression levels were calculated using the delta-delta C_q ($\Delta\Delta C_q$) method. Negative controls, including no-template and no-reverse transcriptase reactions, were included. Detection primers for *CUL4B, TNF, IL1B*, *CTSL*, *CTSB*, *CTSD, NLRP3* and *GAPDH* were used, with their sequences provided in Table S1 and purchased from Integrated DNA Technologies (IDT).

IFNAR signaling assay. THP-1 Dual and THP-1 Dual KO-IFNAR2 (ΔIFNAR2) cells, carrying an IRF-inducible Lucia luciferase reporter gene, were seeded in 96-well plates (0.1 × 10^6 cells/well) in RPMI/FBS and differentiated with 25 nM PMA according to the manufacturer's guidelines (InvivoGen). After 24 hours, cells were washed and replenished with fresh RPMI/FBS. MRSA cultures were prepared as described in "Bacterial strains and growth conditions" washed with PBS, and resuspended in RPMI/FBS at the desired CFU. Forty-eight hours post-differentiation, cells were infected with MRSA (MOI = 1) for 1 hour at 37°C with 5% CO_2. Uninfected cells (mock) served as controls. Cells were then washed with PBS, and extracellular bacteria were eliminated by replacing the medium with RPMI/FBS containing 40 µg/mL lysostaphin and 100 µg/mL gentamicin. Luciferase activity was measured in the cell culture supernatant 18 hours post-infection using the QUANTI-Luc kit, following the manufacturer's guidelines (InvivoGen).

Confocal microscopy analysis. THP-1 cells were seeded and differentiated on 8-well chamber coverslips (IBIDI, 0.2 × 10^6 cells/well) and either left untreated or infected with MRSA USA300 TCH 1516 pcm29 (MRSA-GFP) (*160*) (MOI = 10) as described in "Bacterial internalization and cytotoxicity assay". Two hours post-infection (1 h post-ABX treatment), cells were washed with PBS, fixed with 4% paraformaldehyde (Thermo Fisher Scientific), and permeabilized with 0.1% Triton X-100 (Sigma-Aldrich) for 5 minutes at room temperature. Cells were blocked with 3% goat serum for 45 minutes with gentle shaking, then incubated with phospho-STAT1 (Tyr701) rabbit monoclonal antibody (Cell Signaling, 9167, 1:500, UniProt ID: P42224) for 2 h at room temperature. After five PBS washes (2 minutes each), cells were incubated with Alexa Fluor 647 secondary antibody (Invitrogen, A-21245, 1:750, PRID: AB_2535813) for 1 h at room temperature, followed by additional PBS washes. Actin was stained with TRITC-phalloidin (Life Technologies, 1:750) for 30 minutes, and nuclei were stained with Hoechst 33342 (1 µg/mL, Invitrogen) for 5 minutes. Slides were mounted with VECTASHIELD antifade mounting medium (Vector Laboratories). Images were captured using a TCS SP5 confocal microscope (Leica Microsystems) equipped with a 63× NA 1.4 Plan Apochromat objective.

Reactive oxygen species (ROS) assay. WT and ΔCUL4B THP-1 cells were seeded and differentiated in 96-well black/clear plates (0.1 × 10^6 cells/well), then either left untreated or infected with MRSA USA300 (MOI = 1) as described in "Bacterial internalization and cytotoxicity assay". Six hours post-infection (5 h post-antibiotic treatment), the cells were washed with PBS and incubated in Hanks' Balanced Salt Solution containing calcium and magnesium (Gibco, Life Technologies) supplemented with 10 µM 2′,7′-dichlorofluorescin diacetate (DCFH-DA; Sigma-Aldrich) for 30 minutes at 37°C. The cells were then washed with PBS, and fluorescence intensity was measured using a plate reader (485 nm excitation, 530 nm emission).

Proteomics profiling of MRSA-infected WT and ΔCUL4B THP-1 macrophages and data analysis. THP-1 cells were seeded in 6-well plates (2 × 10^6 cells/well) in RPMI/FBS and differentiated with 25 nM PMA. The experiment was performed with 12 × 10^6 cells per condition per biological replicate. Twenty-four hours post-differentiation, cells were washed and replenished

with fresh medium. Forty-eight hours post-differentiation, cells were infected with MRSA (MOI = 10) for 1 h at 37°C with 5% CO_2. Uninfected cells (mock) served as controls. Cells were then washed with PBS, and extracellular MRSA was eliminated by replacing the medium with RPMI/FBS containing 40 µg/mL lysostaphin and 100 µg/mL gentamicin. Where indicated, cells were either left untreated or treated with cycloheximide (CHX, 100 µg/mL, Sigma-Aldrich) and MG132 (10 µM, InvivoGen).

At 1, 2, 3, and 4 h post-infection (depending on the experimental condition specified in the figure legends), cell culture supernatants and corresponding cell pellets were collected. A total of 250 µL of whole-cell lysate or bacterial culture supernatant was mixed with 250 µL of ice-cold ProtiFi lysis buffer containing 5% SDS, 50 mM triethylammonium bicarbonate (TEAB, pH 8.5), 1× Complete Mini EDTA-free protease inhibitor (Roche), and 1× PhoSTOP EAZY phosphatase inhibitor (Roche). Cells were lysed by vigorous vortexing and shaking for 30 minutes, followed by a 5-minute water bath sonication to ensure complete membrane disruption. Cell debris was removed by centrifugation (10,000 × *g*, 5 min).

Lysed samples were reduced with 5 µL of 500 mM DTT for 45 minutes at 37°C. Following reduction, proteins were alkylated with 15 µL of 500 mM IAA for 45 minutes at room temperature in the dark. Alkylation was quenched with 20 µL of 500 mM DTT, and proteins were acidified with 27 µL of 12% phosphoric acid. Denatured proteins were loaded onto ProtiFi S-Trap mini columns and processed per the manufacturer's instructions (ProtiFi, K02-mini-80). Proteins were digested for 4 h at 37°C with 2.5 µg of sequencing-grade trypsin (Promega, PRV5117) in 50 mM TEAB, pH 8.5. Peptides were subsequently eluted by sequential centrifugation (1,000 × *g*, 1 minute) with 125 µL of 50 mM TEAB, 125 µL of 5% formic acid, and 125 µL of 50% acetonitrile/5% formic acid. Eluted peptides were frozen at −80°C and lyophilized using a Savant SPD111V SpeedVac Concentrator (Thermo Fisher Scientific) with a −105°C Savant RVT5105 Refrigerated Vapor Trap (Thermo Fisher Scientific) and vacuum supplied by a Fisher Scientific Maxima D4A Rotary Vane Pump (Thermo Fisher Scientific). Lyophilized peptides were stored at −80°C until desalting. For desalting, peptides were resuspended in 1 mL of 0.1% trifluoroacetic acid (TFA), vortexed for 20 minutes, sonicated in a Branson 2800 water-bath sonicator (Branson Ultrasonic) for 5 minutes, and centrifuged to remove insoluble debris. Purified peptides were loaded onto 50 mg C18 Sep-Pak 1 cc vac cartridges (Waters, WAT054955), pre-conditioned with two washes of 100% acetonitrile and three washes of 0.1% TFA. Peptides were loaded onto the column and washed with 0.1% TFA (five times), 0.5% acetic acid (once), and eluted with 40% acetonitrile/0.5% acetic acid followed by 80% acetonitrile/0.5% acetic acid. Eluted peptides were frozen at −80°C and lyophilized as described above. Peptides were reconstituted in 1 mL of 50% acetonitrile and quantified using the Pierce Peptide Colorimetric Quantitative Assay Kit (Thermo Fisher Scientific, 23275) per the manufacturer's instructions. A total of 50 µg of peptides was aliquoted for TMT labeling and freeze-dried as previously described. For non-TMT-labeled samples (data-independent acquisition; DIA), 30 µg of peptides was aliquoted per sample, frozen at -80°C, and lyophilized.

Lyophilized peptides set for TMT-labeled proteomics were resuspended in 50 µL of 30% dry acetonitrile in 50 mM HEPES (pH 8.5), vortexed for 20 minutes, sonicated in a Branson 2800 water-bath sonicator (Branson Ultrasonic) for 5 minutes, centrifuged (10,000 × *g*, 5 min), vortexed for 20 minutes, and sonicated in a Branson 2800 water-bath sonicator (Branson Ultrasonic) for 5 minutes. TMT-10plex (Thermo Fisher Scientific, TMT lot number YK379479) labels were resuspended in the same buffer, and 8 µL of each TMT label was added per sample (randomized), incubated at room temperature for 1 h, and quenched with 5% hydroxylamine for 15 minutes, vortexed, and sonicated in a Branson 2800 water-bath sonicator (Branson Ultrasonic) for 5 minutes. Reactions were acidified with 50 µL of 1% TFA, combined into one plex, frozen at −80°C, and lyophilized before desalting as previously described. Labeled samples were fractionated using the Pierce Peptide Fractionation Kit (Thermo Fisher Scientific, 84868), yielding eight fractions, which were freeze-dried before LC-MS^3 analysis. For LC-MS^3 analysis, freeze-dried fractions were resuspended in 30 µL of 5% acetonitrile/5% formic acid, vortexed for 20 minutes, sonicated in a

Branson 2800 water-bath sonicator (Branson Ultrasonic) for 5 minutes, diluted 1:3 in the same buffer, centrifuged (13,000 rpm, 5 min), vortexed for 20 minutes, sonicated in a Branson 2800 water-bath sonicator (Branson Ultrasonic) for 5 minutes, and transferred to glass vials (Thermo Fisher Scientific). LC-MS^2/MS^3 was performed using an Orbitrap Fusion mass spectrometer coupled to an Easy-nLC 1000. Fractions were run on a 180-min gradient, progressing from 3% to 100% acetonitrile in 0.125% formic acid. Peptides were separated using an EASY-Spray PepMap Neo C18 column (150 mm × 75 µm, Thermo Fisher Scientific), with electrospray ionization at 2000V.

For non-TMT labeled samples and DIA mass spectrometry, lyophilized peptides were reconstituted in 100 µL of 5% acetonitrile and 5% formic acid (FA), followed by vortexing for 20 minutes. Samples were then sonicated (water bath) for 5 minutes and centrifuged (10,000 × *g*, 5 min). The clarified supernatants were transferred into SureSTART 2 mL vials and loaded into the VanquishNeo autosampler. Subsequently, 1 µL of each sample was injected and separated on a reverse-phase EASY-IC spray LC column (Aurora Elite, 15 cm × 75 µm, 1.7 µm C18 column; IonOptiks AUR3-15075C18-XT) coupled to a trap/loop column (PepNeo, 300 µm x 5 mm, 5 µm C18; Thermo Fisher Scientific, 174500). Chromatographic separation was performed on an Astral mass spectrometer (Thermo Fisher Scientific) using a 23-min DIA method with an 80% ACN gradient at a flow rate of 100 µL/min.

For TMT-labeled samples, MS^2 data were processed using Proteome Discoverer 2.5.0.400 (ThermoFisher Scientific) and searched against the human reference proteome (UP000005640, downloaded 12-14-2023) using the Sequest algorithm (*138*), including a decoy database for false discovery rate (FDR) estimation (*139*–*141*). Search parameters included a 50 ppm MS^1 mass tolerance (*142*, *143*), a 0.6 Da MS^2 mass tolerance, static modifications for TMT 10-plex and TMTpro tags on lysine/N-termini (+229.162932 Da), carbamidomethylation of cysteines (+57.02146 Da), and variable oxidation of methionine (+15.99492 Da) (*130*). Percolator was used to achieve 1% FDR at the peptide and protein levels (*161*, *162*). Quantitative analysis was based on TMT reporter ion intensities from MS^3 spectra, using signal-to-noise (S/N) values for downstream quantitation (*134*, *145*). Normalized summed S/N ratios per protein per sample were used, with further normalization to the dataset median to account for minor variations in labeled protein amounts. Missing values were replaced with the dataset median for downstream binary comparisons (*130*). For DIA samples, which were run on the orbitrap Astral mass spectrometer, raw DIA spectra were searched against the human reference proteome (UP000005640, downloaded 08-18-2025) using DIA-NN (*163*). The resulting *report.parquet* files were processed in FragPipe-Analyst (*164*) for protein group and peptide group quantification, as well as global data normalization using DIAgui (*165*). Missing values were imputed using a "Perseus-style" approach.

For TMT-labeled samples, statistical analysis was performed on average protein abundances using ANOVA followed by a Tukey HSD post-hoc test. For non-TMT-labeled samples using FragPipe-Analyst software (*164*), in the differential expression analysis framework, individual features, encompassing either proteins or peptides, are interrogated using feature-specific linear models in conjunction with empirical Bayesian moderation as implemented in limma (*166*). Both nominal *p*-values and adjusted *p*-values according to the Benjamini–Hochberg procedure were reported. Proteins with FC ≥ 1.5 ($\log_2$ FC ≥ 0.585) or FC ≤ −1.5 ($\log_2$ FC ≤ −0.585) and *P*-value/adjusted *P*-value ≤ 0.05 were analyzed for Gene Ontology (GO) enrichment and network analysis using Metascape (v3.5.20240901) (*121*) and STRING (*152*). Molecular function annotation was conducted using the Panther knowledgebase (*167*, *168*).

Prediction of CUL4B structure. Predictions were performed using the complete CUL4B sequence (UniProt ID: Q13620) and the AlphaFold 3 server (*169*). The image was generated using PyMOL v3.0.4.

Real-time cytotoxicity analysis using Incucyte. WT and ΔCUL4B THP-1 cells were seeded into 96-well plates (1×10^5 cells/well) in RPMI/FBS and differentiated with PMA. Cells were either left uninfected or infected with MRSA at MOIs of 0.5, 2, and 4, as described in the "Bacterial internalization and cytotoxicity assay" section. One hour post-infection, cells were washed with PBS, and extracellular MRSA was eliminated by replacing the medium with RPMI/FBS supplemented with lysostaphin, gentamicin, and propidium iodide (PI) (Life Technologies, final concentration 1 μg/mL). Plates were immediately transferred to the IncuCyte SX5 (Sartorius BioAnalytical Instruments) and scanned hourly for 24 h, capturing four frames per well (magnification = 20×) using the G/O/NIR Optical Module. Analysis was completed with the Basic Analyzer Optical Module software (IncuCyte) using a top-hat segmentation radius of 100 μm, and an Orange Mean Intensity threshold of 2. Events per frame were averaged at each time point and plotted as mean ± SD from a representative experiment conducted in triplicate.

Caspase-1 activation analysis. WT and ΔCUL4B THP-1 cells were seeded into 12-well plates (1×10^6 cells/well) for flow cytometry or onto 8-well chamber coverslips (IBIDI, 2×10^5 cells/well) for microscopy analysis in RPMI/FBS and differentiated with PMA. Cells were either left uninfected or infected with MRSA (MOI = 2.5–5, 6 hpi) as described in the "Bacterial internalization and cytotoxicity assay" section. When applicable, LPS-treated cells (1 μg/mL, InvivoGen) followed by ATP stimulation (5 mM, InvivoGen) served as positive controls. At 6 hours post-infection, caspase-1 activity was assessed using the fluorescent FAM–FLICA caspase-1 (YVAD) kit (ImmunoChemistry Technologies) per the manufacturer's instructions. For flow cytometry, cells were additionally stained with DRAQ7 (Invitrogen, 3 μM) for 10 minutes at room temperature before analysis on a FACSCanto II (BD Biosciences) using FlowJo software. The percentage of FLICA–caspase-1–positive cells quantified caspase-1 activation in response to MRSA infection. The gating strategy is shown in fig. S14. For microscopy, cells were further stained with Hoechst (ImmunoChemistry Technologies) and phalloidin-rhodamine (Thermo Fisher Scientific), mounted, and imaged using a Zeiss Axio Observer.D1 Inverted Microscope. Images were processed in Fiji, with the region of interest (ROI) established using phalloidin signals. Pre-processing included application of the Log function, rolling ball subtraction (radius = 70), and a minimum filter (radius = 2). The Li algorithm was used for thresholding. The same pre-processing steps were applied to the FAM-FLICA channel, and ROI was measured for area, integrated density, and median intensity. Raw integrated density was normalized by area to quantify FAM-FLICA intensity. Data were represented as mean RawIntDen/area (normalized to mean background).

Neutrophil isolation, serum collection, and killing assay. Neutrophils were isolated from heparinized venous blood collected from healthy adult volunteers (18 to 60 years of age; male and female) using 1-Step Polymorphprep (Fresenius Kabi Norge AS) by density-gradient centrifugation (*170*). Pooled normal human serum (NHS) was prepared from non-heparinized clotted blood collected from multiple healthy donors by centrifugation (15 min, 2000 × *g*), pooled, and stored at −80°C (*170*). Ethics declarations are described in the respective section. For the neutrophil killing assay, freshly isolated human neutrophils were pretreated with vehicle or increasing concentrations of MLN4924 for 30 minutes before bacterial inoculation. MRSA cultures were prepared as described in "Bacterial Strains and Cell Lines," washed with PBS, and resuspended in RPMI to the desired CFU concentration. Bacteria were opsonized with 5% pooled NHS for 5 min at 37°C with shaking in low-binding Eppendorf tubes and then added to purified human neutrophils (5×10^5) at the indicated MOI and incubated for 30 minutes at 37 °C on a horizontal rotator. Cells were lysed with ice-cold H_2O supplemented with 0.3% (w/v) saponin, and bacterial survival was assessed by serial dilution in PBS, plating on TH agar, overnight incubation at 37 °C, and colony counting.

Murine model of intratracheal (IT) MRSA infection. IT infection was performed as previously described (*170*). C57BL/6 mice (Jackson Laboratory; male and female; 8 weeks old), wild-type ($CUL4B^{fl/y}$; male; 8 to 11 weeks old; n = 8 mice per group), and ΔCUL4B ($CUL4B^{\Delta/y}$; male; 8 to 11 weeks old; n = 7 mice per group) mice were used for the *in vivo* studies. WT and ΔCUL4B mice

were provided by Prof. Zhou (Weill Cornell Medicine) and generated as previously described (*41*, *46*). Briefly, the *CUL4B* knockout allele ($CUL4B^{\Delta/y}$) was generated by a germline-induced deletion of exons 4 and 5, achieved by crossing floxed *CUL4B* mice ($CUL4B^{fl/y}$) with the *SOX2-Cre* transgenic line. $CUL4B^{fl/y}$ mice carry a floxed *CUL4B* allele, with exons 4 and 5 flanked by loxP sites (*41*, *46*). No formal randomization was performed for comparisons between genotypes, as mice were assigned to experimental groups based on genotype. When indicated, C57BL/6J mice (male and female, n = 9–10 mice per group) were randomly assigned to receive intraperitoneal administration of MLN4924 (HY-70062, MedChemExpress) or vehicle in two doses. The first dose (30 mg/kg) was given 24 h prior to infection, and the second dose (10 mg/kg) was administered 0.5 h before the MRSA IT challenge. Sample sizes were determined based on statistical power considerations and prior studies in the field. No animal was excluded from analysis unless a technical failure occurred (e.g., unsuccessful intratracheal instillation, failure to recover from anesthesia). Details of inoculum, sex of mice, and the number of mice per experiment are provided in the figure legends. An overnight MRSA culture was grown in THB at 37 °C with shaking (200 rpm), washed with PBS, and resuspended to the indicated inoculum doses. WT and ΔCUL4B mice received 5×10^7 CFU/30 µL per mouse, whereas MLN4924-treated mice received sublethal doses of 1×10^7 or 7×10^7 CFU/30 µL per mouse (designated medium- and high-doses in Fig. 3L, respectively) or a lethal dose of 1.8×10^8 CFU/30 µL per mouse. Bacterial titers were confirmed by serial dilution and plating on TH agar. Mice were anesthetized with ketamine (100 mg/kg) and xylazine (10 mg/kg), and MRSA was delivered intratracheally using a long sterile pipette tip under visualization with an otoscope (Welch Allyn). Mice were placed on a warming pad for recovery. For survival analysis, mice were monitored every 12 h for the first 48 h and every 24 h thereafter, and deaths were recorded. At 24 h post-infection, mice were euthanized, and lungs were homogenized using a Mini BeadBeater (BIOSPEC Products). Homogenates were serially diluted in PBS, plated on TH agar for CFU enumeration, and reported as CFU/g lung tissue. Supernatants were centrifuged (10,000 × *g*, 10 min, 4 °C) and stored at −80 °C for cytokine analysis. When applicable, blood was collected using BD Microtainer tubes with or without K_2E (K_2EDTA) anticoagulant for plasma and serum preparation, respectively. Serum was used for proteomics and LDH analysis (LDH-Glo cytotoxicity assay, Promega), following the manufacturer's instructions. K_2E-treated blood was used for systemic bacterial burden assessment and complete blood count (CBC) analysis. CBC was performed within 2 h of collection at the UCSD Murine Hematology and Coagulation Core Laboratory using a Hemavet 950FS Multi-Species Hematology System (Drew Scientific) with mouse-specific settings in a blinded manner.

Lung and spleen histopathology. Lungs and spleens from intratracheally challenged WT and ΔCUL4B mice (n = 5 mice per group) were fixed in 10% paraformaldehyde (PFA) for 24 h, then transferred to 70% ethanol for long-term storage. Tissue sectioning and hematoxylin and eosin (H&E) staining were performed at the Moores Cancer Center Biorepository and Tissue Technology Core. Sections were deparaffinized using xylene, rehydrated through an ethanol/water gradient, and rinsed with deionized water. Rehydrated slides were stained with H&E and scanned using a Leica Aperio AT2 Whole Slide Scanner. Histopathological scoring was performed in a blinded manner. Digital slides were analyzed using ImageScope software.

Analysis of cytokine and cholesterol levels. Cell culture supernatants from MRSA-infected WT and ΔCUL4B THP-1 cells were collected, centrifuged (2000 × *g*, 10 min, 4 °C), and stored at −80 °C. Cytokine levels were measured using the Bio-Plex Pro™ human cytokine assay (Bio-Rad) or the human DuoSet ELISA kit (R&D) according to the manufacturer's instructions. The lung homogenates and BMDM culture supernatant were analyzed using the Bio-Plex Pro™ mouse cytokine assay (Bio-Rad) or the mouse DuoSet ELISA kit (R&D), following the manufacturer's guidelines. Mouse sera were also used to measure cholesterol levels using mouse LDL- and HDL-cholesterol kits (Crystal Chem).

Proteome analysis of murine lung and serum. Pre-homogenized whole lung samples (50 µL) and serum (10 µL) from intratracheally challenged WT (n = 5) and ΔCUL4B (n = 5) mice were

thawed on ice and subjected to albumin and immunoglobulin depletion using HSA/Immunoglobulin mini spin columns (Thermo Fisher Scientific) per the manufacturer's instructions. Depleted lung homogenates or serum (180 μL) were mixed with an equal volume of ProtiFi lysis buffer, vortexed for 20 min, sonicated for 5 min, and centrifuged (10,000 × *g*, 5 min, RT) to remove debris. Protein digestion, desalting, and TMT labeling (Thermo Fisher Scientific, TMT lot number YL379187) were performed as described in "Proteomics profiling of MRSA-infected WT and ΔCUL4B THP-1 macrophages and data analysis" For basic pH reverse-phase chromatography fractionation, labeled peptides were resuspended in 25 mM ammonium bicarbonate, processed via HPLC (BioBasic-18 C18 column, Thermo Fisher Scientific), and separated into 24 fractions, which were combined into 12 distinct fractions and freeze-dried before multiplexed LC-MS^3 analysis. MS data acquisition, spectral processing, and statistical analysis were conducted following the same methods, with lung and serum samples searched against the *Mus musculus* reference proteome (UP000000589, downloaded April 07, 2022).

Quantification and statistical analysis

Data were analyzed and visualized using GraphPad Prism (version 10). Results are presented as mean ± standard error of the mean (SEM) or standard deviation (SD), as specified in the figure legends. Statistical significance ($P < 0.05$) was determined using unpaired *t*-tests, one-way ANOVA, or two-way ANOVA, as appropriate. Further details, including the statistical test used, the *P*-value, the number of experiments, and the number of replicates, are provided in the figure legends. Statistical analyses for transcriptomic and proteomic data are described in their respective sections.

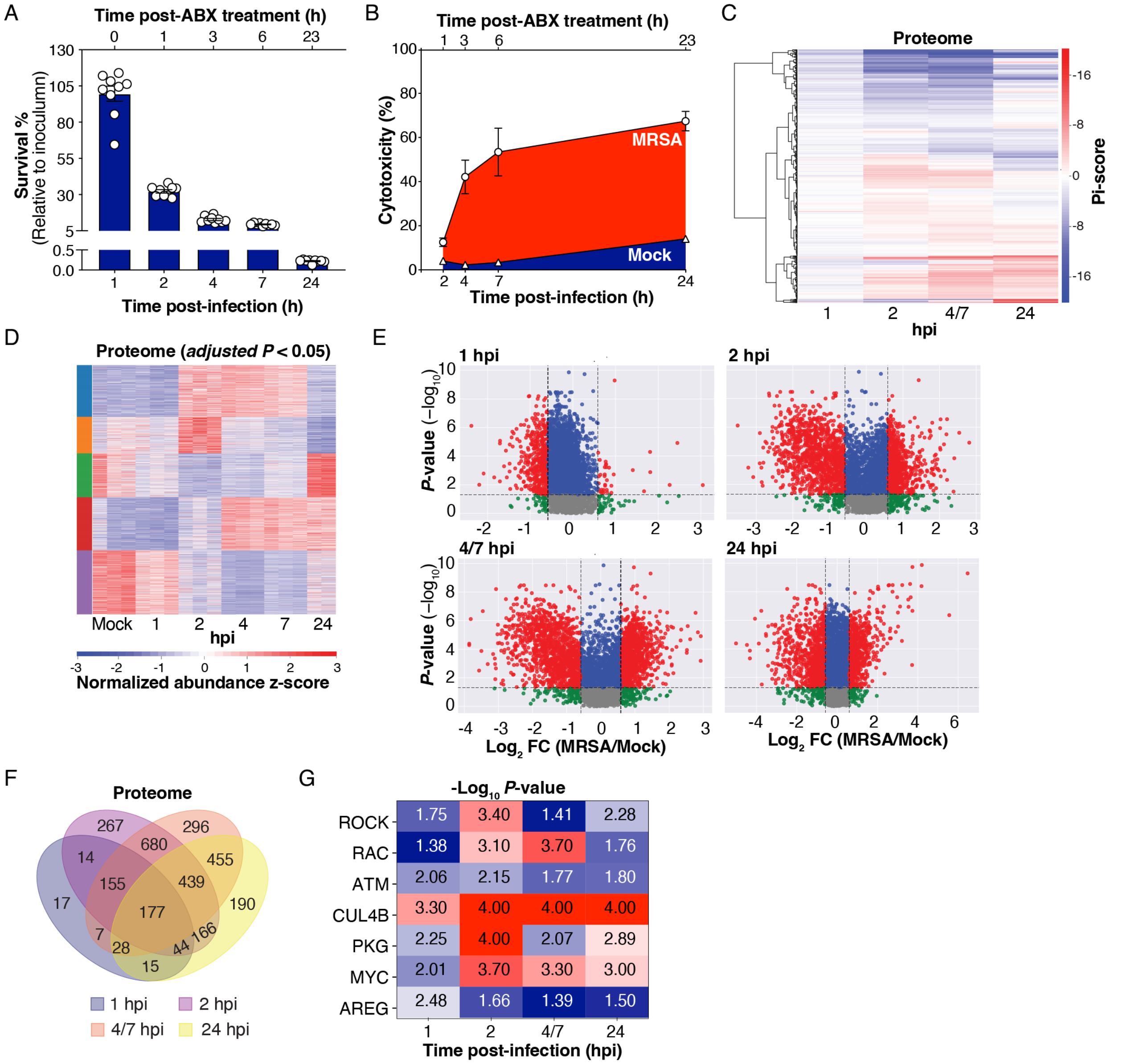

Figure S1. Macrophage proteome responses to MRSA infection. (A) Time course analysis of intracellular MRSA killing by wild-type THP-1 cells (MΦ). Differentiated THP-1 cells (1×10^6 cells/well) were infected with MRSA for 1 hour (MOI = 5), then washed and incubated in medium supplemented with gentamicin and lysostaphin (ABX treatment). Intracellular MRSA was quantified by lysing the cells at 1, 2, 4, 7, and 24 hours post-infection (hpi), and enumerating the bacteria. Survival was calculated relative to inoculum. The data are plotted as mean ± SEM, representing three experiments conducted in triplicate. **(B)** Time course analysis of MΦ viability in response to intracellular MRSA. Differentiated THP-1 cells (1×10^6 cells/well) were infected with MRSA for 1 hour (MOI = 5), then washed and incubated in medium supplemented with gentamicin and lysostaphin as described in A. Macrophage viability was assessed by measuring LDH release in the cell supernatant and normalized to a Triton X-100-positive control. The data are plotted as mean ± SEM, representing two experiments performed in triplicate. **(C)** Heatmap displaying protein abundance in MRSA-infected MΦ cells and mock controls at various time points post-infection for proteins significantly dysregulated at least one time point (adjusted $P \leq 0.05$). The colors represent the normalized count z-score for each protein across all time points. **(D)** Agglomerative hierarchical clustering of pi-scores (π) associated with the proteome datasets in MRSA-infected MΦ cells compared to mock controls. The π scores or protein significance scores were calculated by considering both the *P*-value and fold change as described previously (*171*). **(E)** Volcano plots

showing differentially abundant proteins in the proteome of MRSA-infected MΦ cells relative to mock-infected controls at multiple time points post-infection. The black dotted line(s) through the *y*-axis and *x*-axis, indicate significance cut-offs at adjusted $P = 0.05$ ($-\log_{10} = 1.3$) and fold change (FC) ≥ 1.5 or FC ≤ −1.5 in protein abundance, respectively. Significance was determined using a two-tailed paired *t*-test. **(F)** Venn diagram of differentially abundant proteins (FC ≥ 1.5 or FC ≤ −1.5 and adjusted $P \leq 0.05$) in MRSA-infected MΦ cells and mock controls at various time points post-infection in the proteome datasets (Dataset S2). **(G)** Heatmap displaying the network bias-corrected *P*-values for predicted significantly affected regulators (z-scores ≥ 2 or ≤ −2) shared across all time points in response to MRSA infection (Dataset S7). IPA regulator analysis was conducted using the significantly dysregulated proteins (FC ≥ 1.5 or FC ≤ −1.5 and adjusted $P \leq 0.05$) identified in the proteome dataset for MRSA- compared to mock-infected MΦ cells (Dataset S2). The z-scores and the network of linked dysregulated proteins are shown in Fig. 1C and fig. S6, respectively.

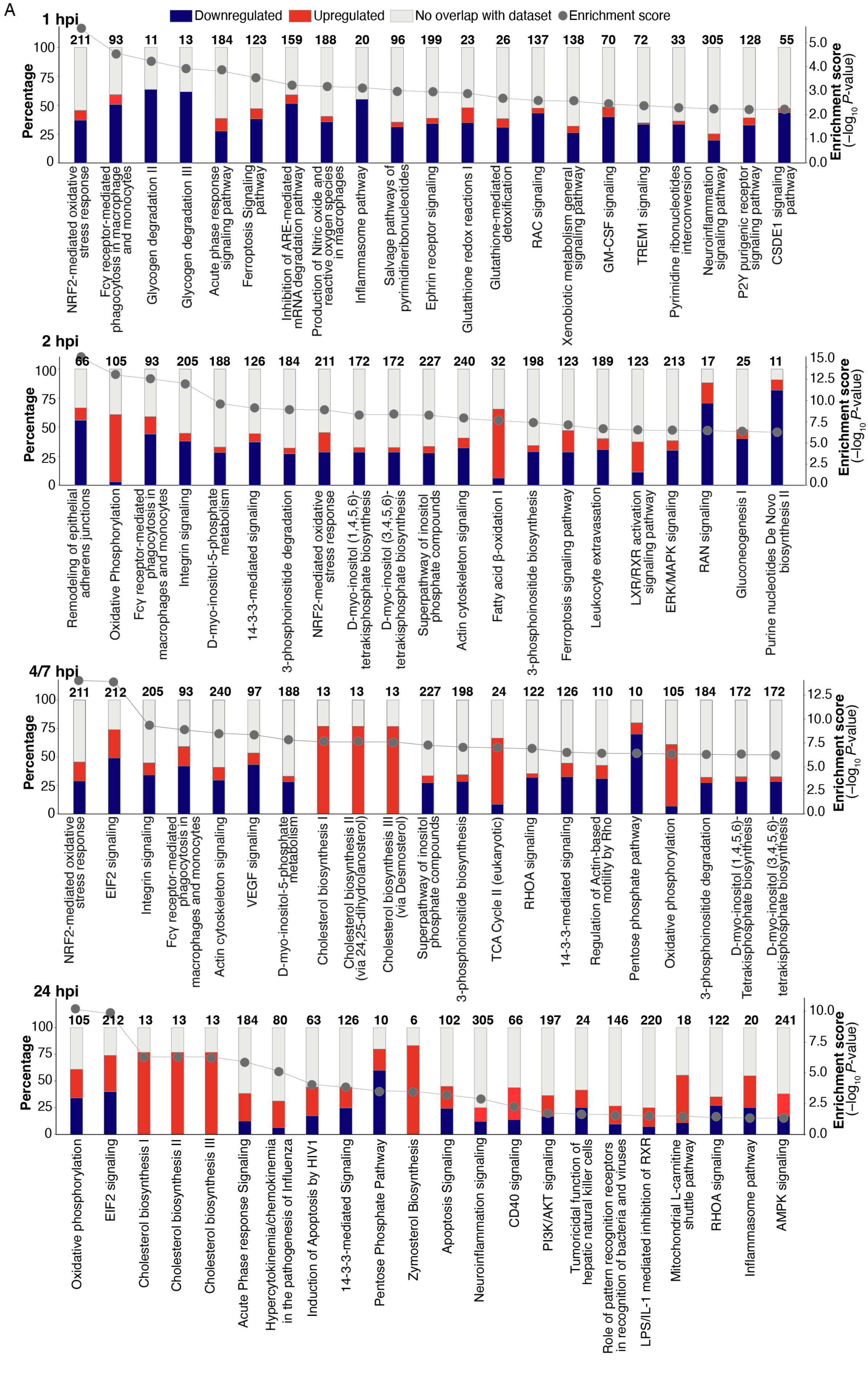
A
Downregulated
Upregulated
No overlap with dataset
Enrichment score
1 hpi
2 hpi
4/7 hpi
24 hpi
Percentage
Enrichment score (−log10 P-value)

Figure S2. Visualizations of the top 21 significantly altered IPA canonical pathways in the proteome of MRSA-infected macrophages. (A) The left y-axis shows the percentage of upregulated (red), downregulated (blue), and non-dysregulated proteins (light grey) in each pathway. The right y-axis shows the enrichment score ($-\log_{10}$ *P*-value) for each pathway. The *P*-value was calculated using a right-tailed Fisher's exact test. Significant canonical pathways ($P \leq 0.05$) are ordered by decreasing enrichment score. The numbers at the top of each bar represent the total number of proteins in that canonical pathway (Dataset S11). Enrichment analysis was performed using the list of significantly dysregulated proteins (Dataset S2) in MRSA-infected MΦ cells compared to mock controls (FC ≥ 1.5 or FC ≤ −1.5 and adjusted $P \leq 0.05$).

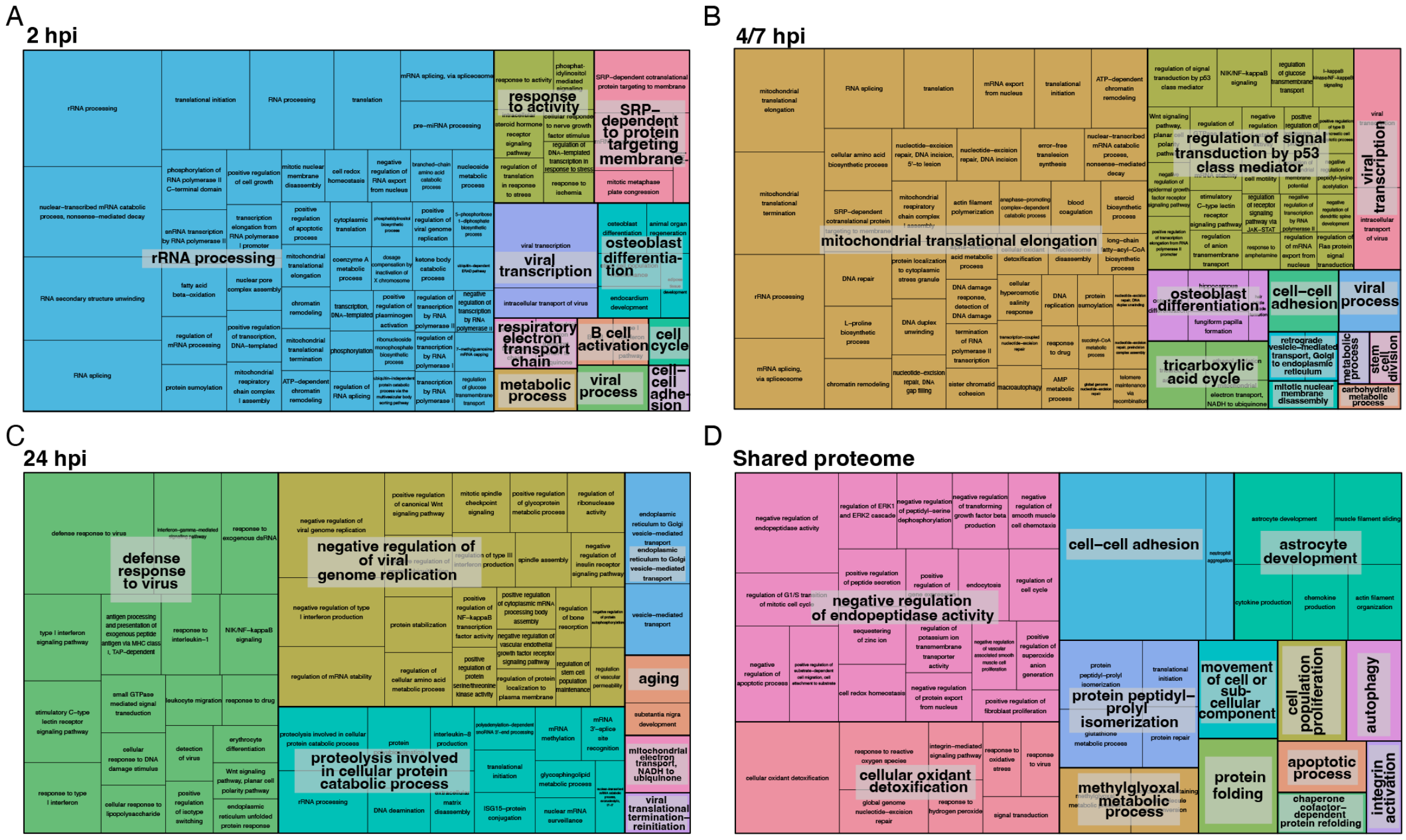

Figure S3. REVIGO Treemaps showing the Gene Ontology (GO) enrichment analysis in the proteome of MRSA-infected macrophages. Gene Ontology (GO) biological process terms were enriched for significantly dysregulated proteins (FC ≥ 1.5 or FC ≤ −1.5 and adjusted $P \leq 0.05$) in the **(A–C)** unique (2, 4/7, 24 hpi) and (D) shared (1, 2, 4/7, 24 hpi) proteomes of MRSA-infected MΦ cells compared with mock controls at multiple time points post-infection. Each rectangle represents a single enriched GO term, and related terms are color-coded. The rectangle size reflects the enrichment *P*-value ($-\log_{10}$) of the GO term.

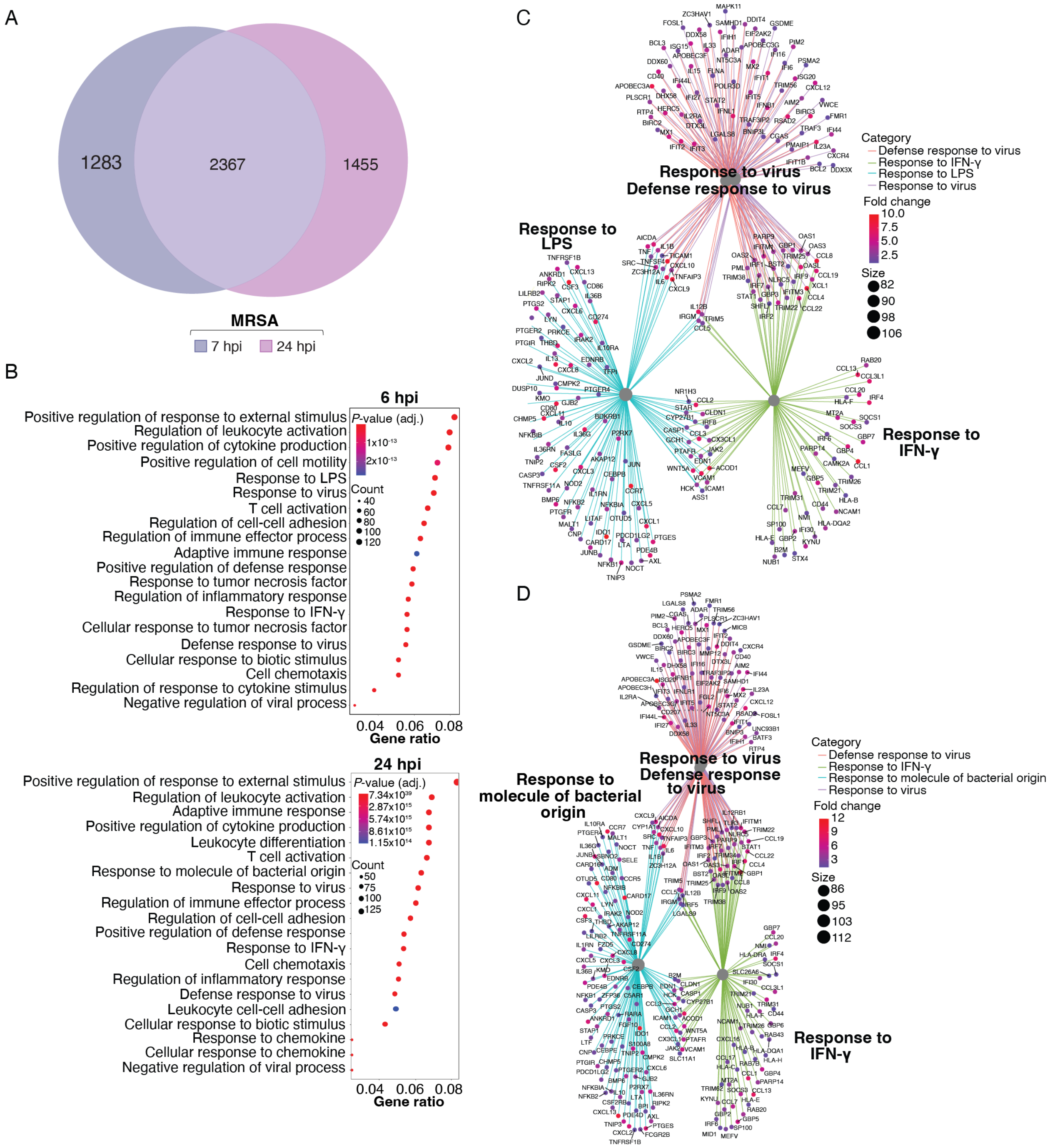

Figure S4. Transcriptomic response of macrophages to MRSA infection. (A) Venn diagram of differentially expressed genes (FC ≥ 2 or FC ≤ −2 and adjusted $P \leq 0.05$) in MRSA-infected MΦ cells and mock-infected controls at 7 and 24 hours post-infection in the transcriptome datasets. **(B)** Dot plot visualizations of the top 20 significantly enriched GO terms (adjusted $P \leq 0.05$) in the transcriptome of MRSA-infected MΦ cells compared to mock-infected controls. Enrichment analysis was performed using the list of significantly upregulated genes (FC ≥ 2 and adjusted $P \leq 0.05$) at 7 (upper panel) and 24 (lower panel) hours post-infection (hpi) using clusterProfiler in R. The size of each dot represents the number of genes associated with the specific GO term, while the color intensity indicates the level of significance. The enrichment *P*-value was calculated based on a cumulative hypergeometric distribution. **(C-D)** Network plots showing selected enriched GO terms from (B) and linked upregulated genes (FC ≥ 2 and adjusted $P \leq 0.05$) in the transcriptome of MRSA-infected MΦ cells compared to mock-infected controls at 7 (C) and 24 (D) hours post-infection (hpi). Grey circles indicate the number of upregulated genes linked to each GO term. The

fold changes in expression are indicated by the dot colors. The plots were generated using clusterProfiler in R.

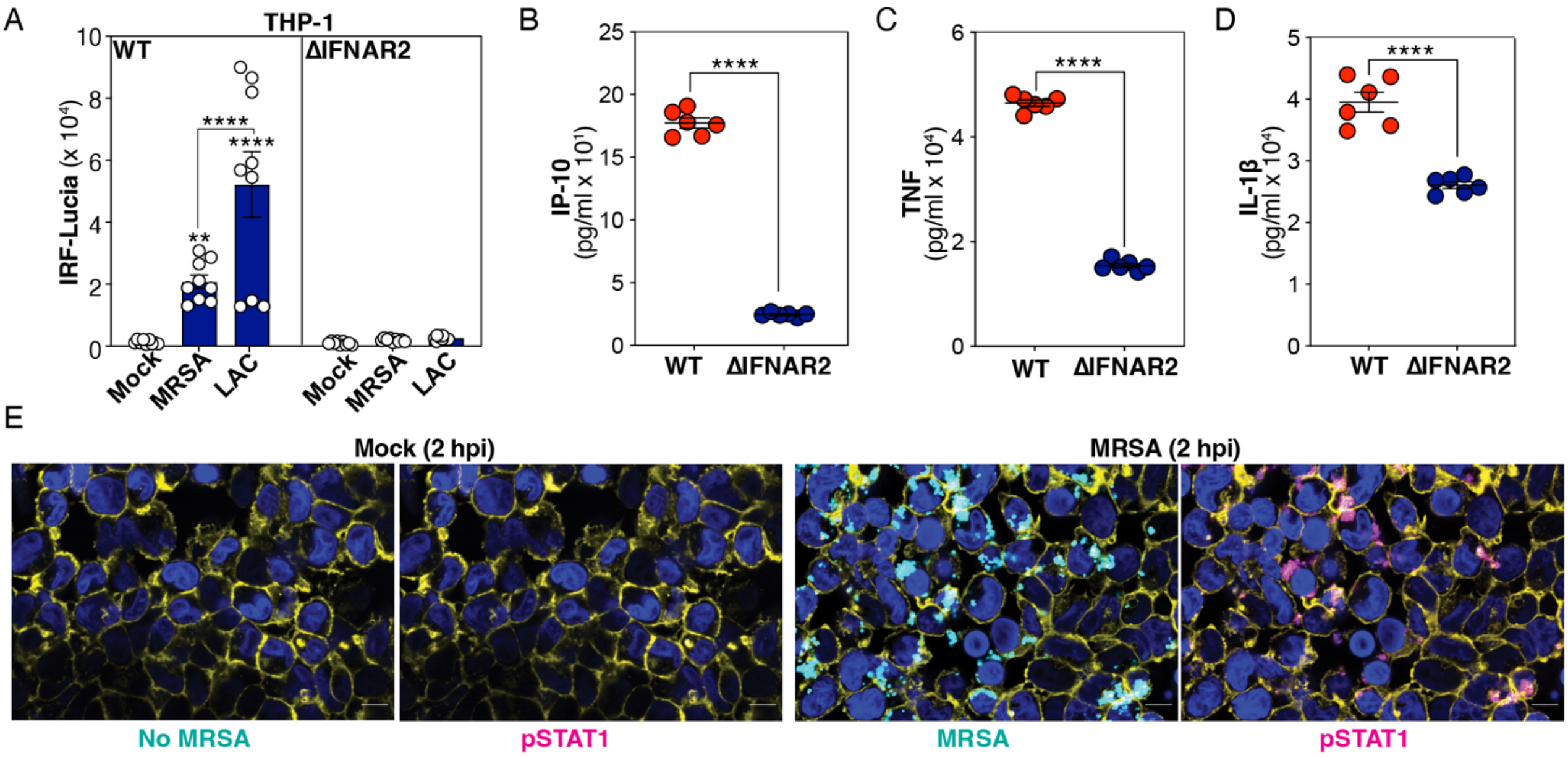

Figure S5. Type I IFN signaling is activated in MRSA-infected macrophages. (A) Analysis of IFNAR signaling activation in WT and ΔIFNAR2 THP-1 Dual cells in response to internalized MRSA. Differentiated THP-1 cells were left uninfected or infected with the TCH1516 and LAC MRSA strains (MOI = 1), were washed and incubated in medium supplemented with gentamicin and lysostaphin. Activation of IFNAR signaling-dependent luciferase expression was assessed in the cell supernatant at 18 hours post-infection (hpi). The data are plotted as mean ± SEM, representing three experiments performed in triplicate, and were analyzed by two-way ANOVA (Tukey's multiple comparisons). Mock vs. TCH1516: $P = 0.0084$; mock vs. LAC: $P < 0.0001$; TCH1516 vs. LAC: $P < 0.0001$. **(B–D)** Quantification of IP-10, TNF, and IL-1β levels in the supernatants of MRSA-infected WT and ΔIFNAR2 cells. Cells were infected as described in (A) (MOI = 5, 6 hpi). The data are plotted as mean (pg/mL) ± SEM, representing two experiments conducted in triplicate, and were analyzed by unpaired two-tailed *t*-test. (B): $P < 0.0001$; (C): $P < 0.0001$; (D): $P < 0.0001$. **(E)** Representative confocal microscopy images of GFP-expressing MRSA- or mock-infected MΦ cells captured at 2 hours post-infection (hpi). Differentiated THP-1 cells were infected with MRSA (MOI = 10). One hour post-infection, the cells were washed and incubated in medium supplemented with gentamicin and lysostaphin. The cells were stained with pSTAT1 antibody, Hoechst, and phalloidin. Three separate channels were merged into a single image using Fiji. The scale bar represents 20 µm. The significant differences are marked with asterisks (*): $^{**}P \leq 0.01$; $^{****}P \leq 0.0001$.

A

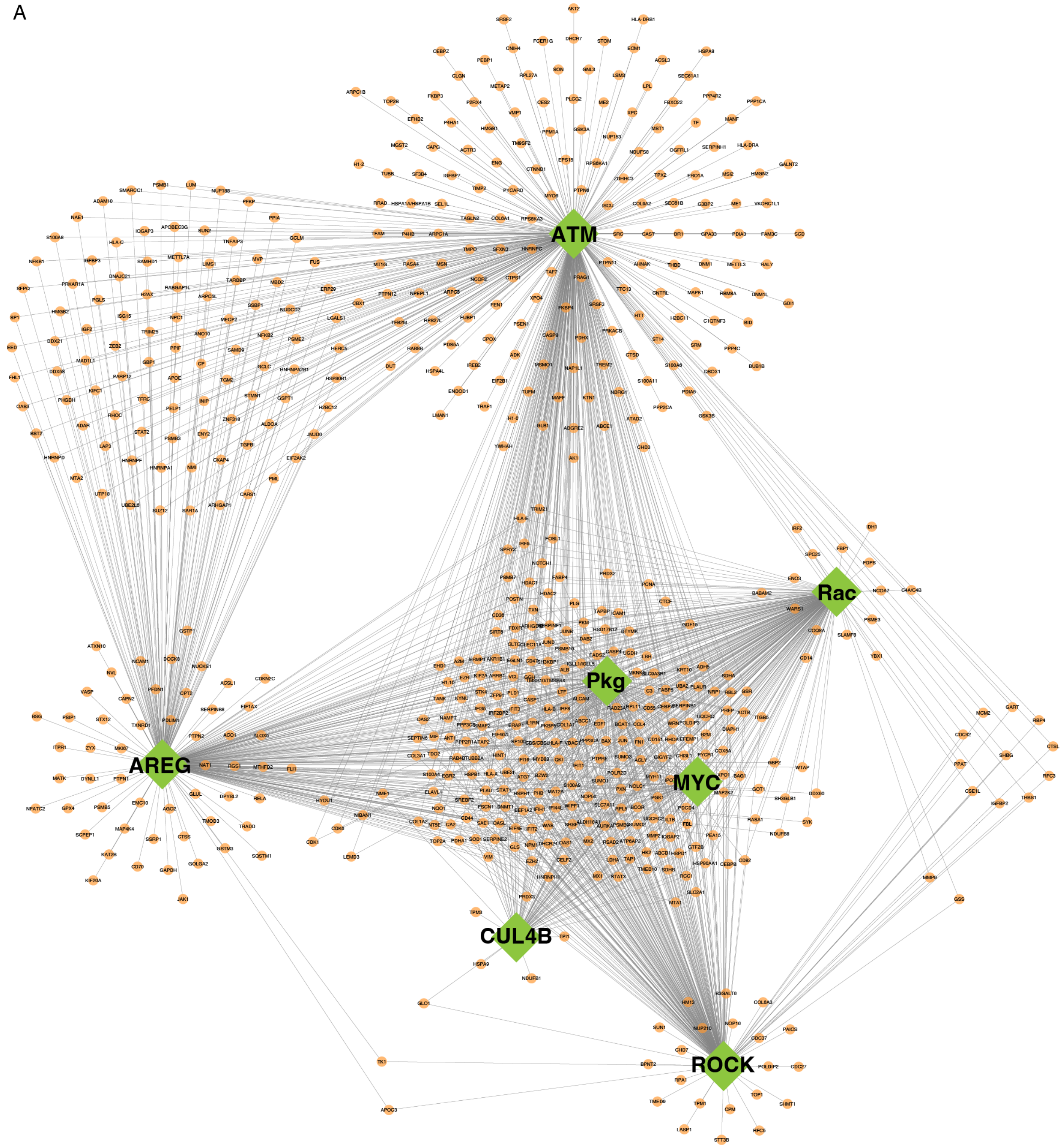

Figure S6. Regulator interaction network. The network displays significantly dysregulated proteins (FC ≥ 1.5 or FC ≤ −1.5, adjusted $P \leq 0.05$) in the proteome dataset of MRSA-infected MΦ cells compared with mock-infected MΦ cells (Dataset S2), linked to affected regulators shared across all time points (Fig. 1C). Regulator analysis was conducted using IPA (z-scores ≥ 2 or ≤ −2, network bias-corrected P-values ≤ 0.05), and the network was created using Cytoscape. The network bias-corrected P-values are shown in fig. S1G.

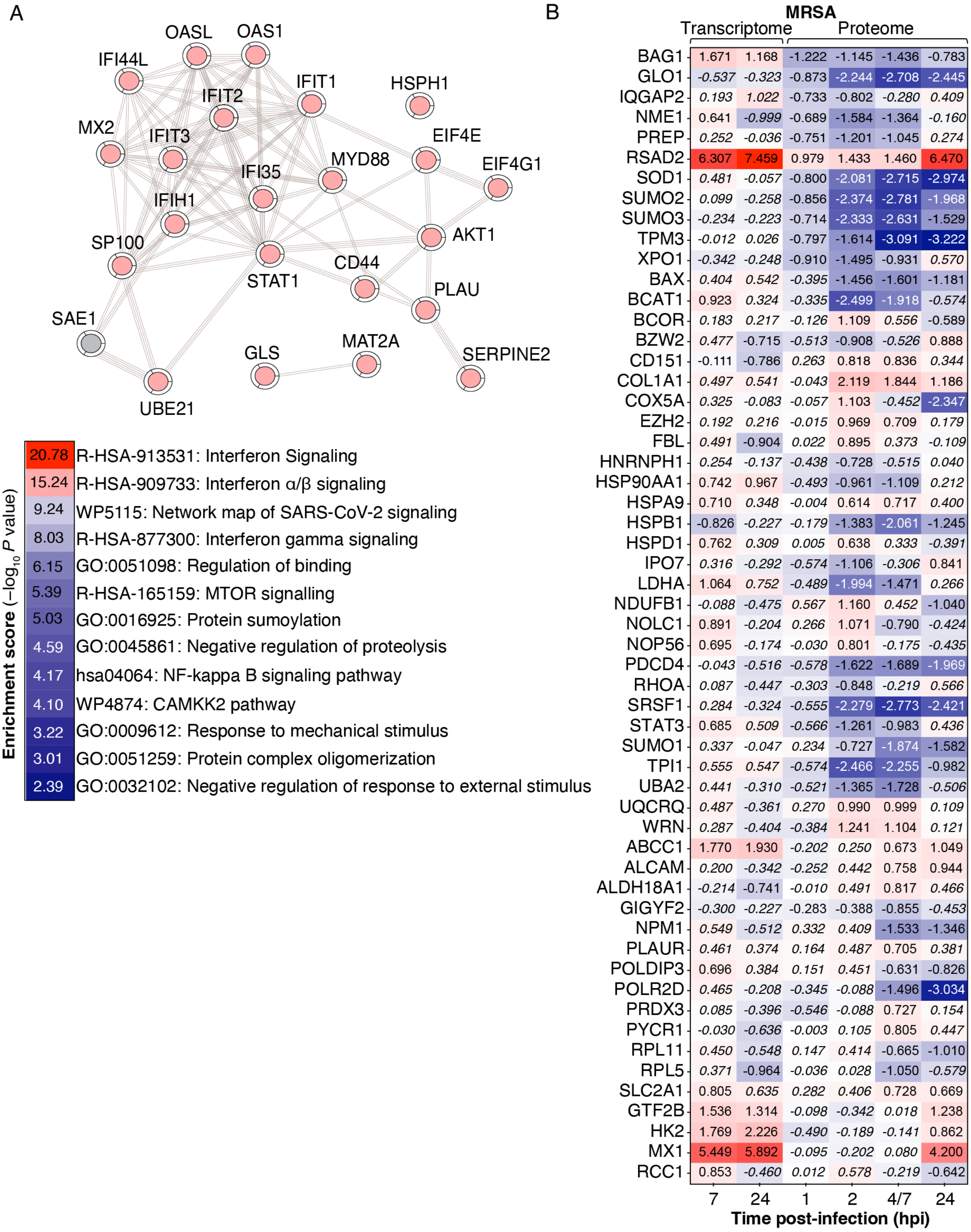

Figure S7. IPA analysis reveals that intracellular MRSA triggers the activation of CUL4B in macrophages. (A) STRING network and enrichment scores ($-\log_{10}$ *P*-value ≥ 2) of significantly regulated (FC ≥ 1.5 or FC ≤ −1.5) genes (adjusted *P* ≤ 0.05) and proteins (adjusted all *P* ≤ 0.05) at one or more time points in MRSA-infected MΦ cells compared with mock-infected controls, shared across all regulators (Dataset S9, Fig. 1F). Enrichment analysis was performed using Metascape (background genes: all genes) (*121*), and the *P*-value was calculated based on a cumulative hypergeometric distribution. The proteins linked to the top three enriched pathways are depicted in the STRING network in pastel red. **(B)** Heatmap showing the average fold changes ($\log_2$) for significantly dysregulated (FC ≥ 1.5 or FC ≤ −1.5) genes (adjusted *P* ≤ 0.05) and proteins (adjusted

all $P \leq 0.05$) at one or more time points in MRSA-infected MΦ cells compared to mock-infected controls, linked to CUL4B. Nonsignificant dysregulated proteins/genes for each time point are marked in *italics* (Dataset S10).

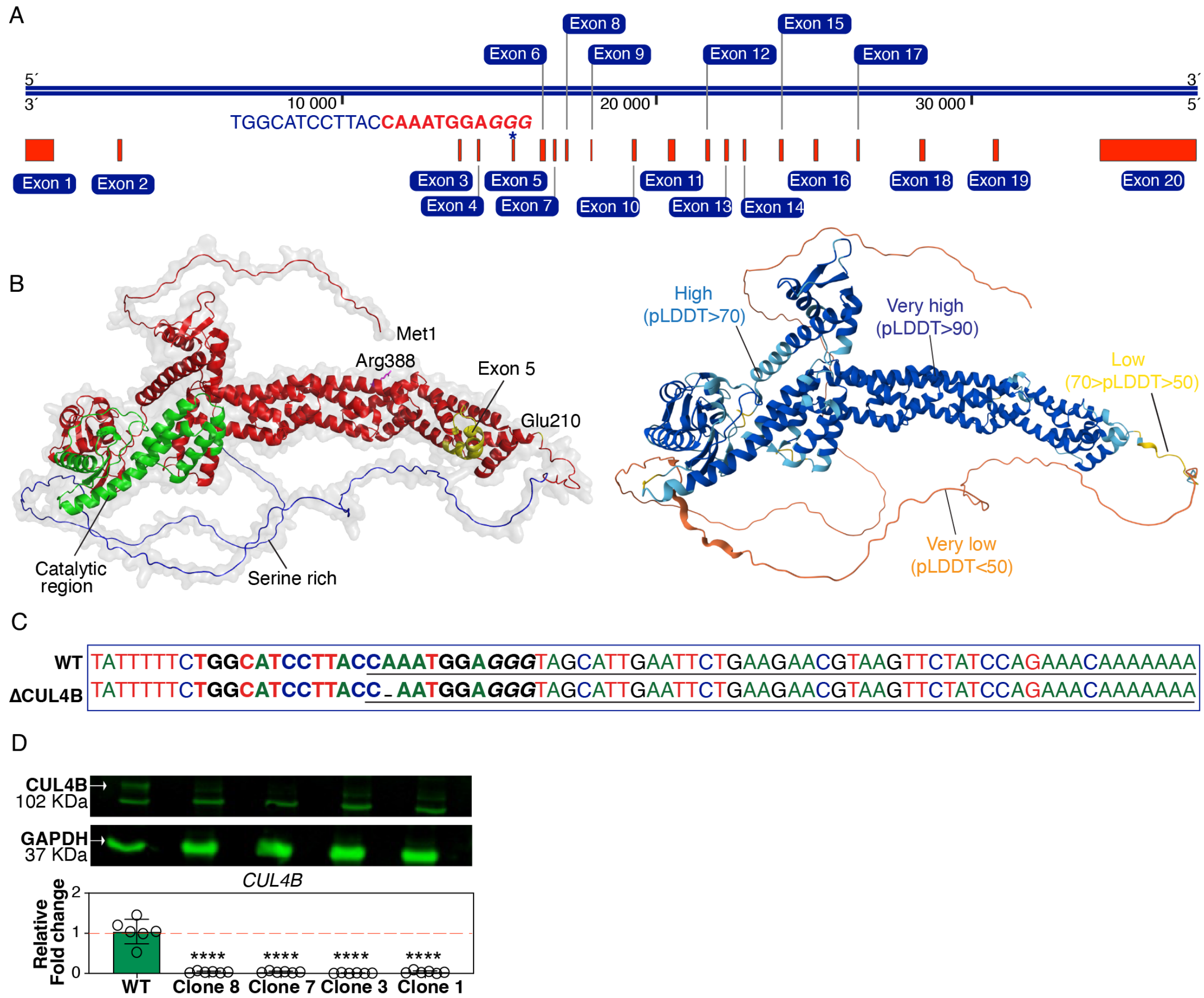

Figure S8. Generation and validation of *CUL4B deletion* in THP-1 cells. (A) A schematic diagram of the genomic structure of the human *CUL4B* gene (RefSeq: NM_001079872.2, GENCODE Transcript: ENST00000371322.11) was generated using SnapGene (37,105 nt). Exons are defined according to RefSeq: NM_001079872.2. The region targeted by the gRNA is indicated by asterisks on the genomic map, and the portion of the gRNA sequence that aligns with exon 5 is highlighted in red/bold, while the PAM sequence is marked in *italics*. **(B)** Left panel: The AlphaFold 3-predicted structure of CUL4B (AF database ID: AF-Q13620-F1) is shown in red-colored cartoon representation with a transparent surface outline. Certain parts of the protein are colored according to the annotation of Zou *et al.* (2007) (*44*): "serine-rich" (residues 61–193; blue), "catalytic" (residues 588–736; green) and Arg544 (purple). The amino acids corresponding to exon 5 of the *CUL4B gene* (as detailed in A) (residues 301–324) are colored yellow. Right panel: The part of the CUL4B protein ranging from amino acid Met1 to Glu210 is predicted to be intrinsically disordered by the AlphaFold 3 algorithm (pLDDT < 50). **(C)** THP-1 cells were treated with a CRISPR/Cas9 protein complexed with gRNA-ΔCUL4B (UGGCAUCCUUACCAAAUGGA, PAM sequence: GGG). Single-cell clones were Sanger sequenced and aligned to the WT THP-1 sequence. Deletion (indels) and the sequences of the gRNA (in bold) and PAM (in bold italics) are indicated. A portion of the sequence from exon 5 is underlined. **(D)** Genetically confirmed ΔCUL4B clones were analyzed by immunoblot and RT-PCR for CUL4B (upper panel) and relative *CUL4B* mRNA expression (lower panel, $2^{-\Delta\Delta Cq}$) in WT and selected ΔCUL4B THP-1 clones, respectively. GAPDH was used as an endogenous control or a loading control. The uncropped immunoblot is shown in fig. S15. The mRNA expression data are plotted as mean ± SD, representing two experiments conducted in triplicate, and were analyzed by two-way ANOVA (Dunnett's multiple comparisons test). WT vs. ΔCUL4B (clones): $P < 0.0001$. The significant differences are marked with asterisks (*): ****$P \leq 0.0001$.

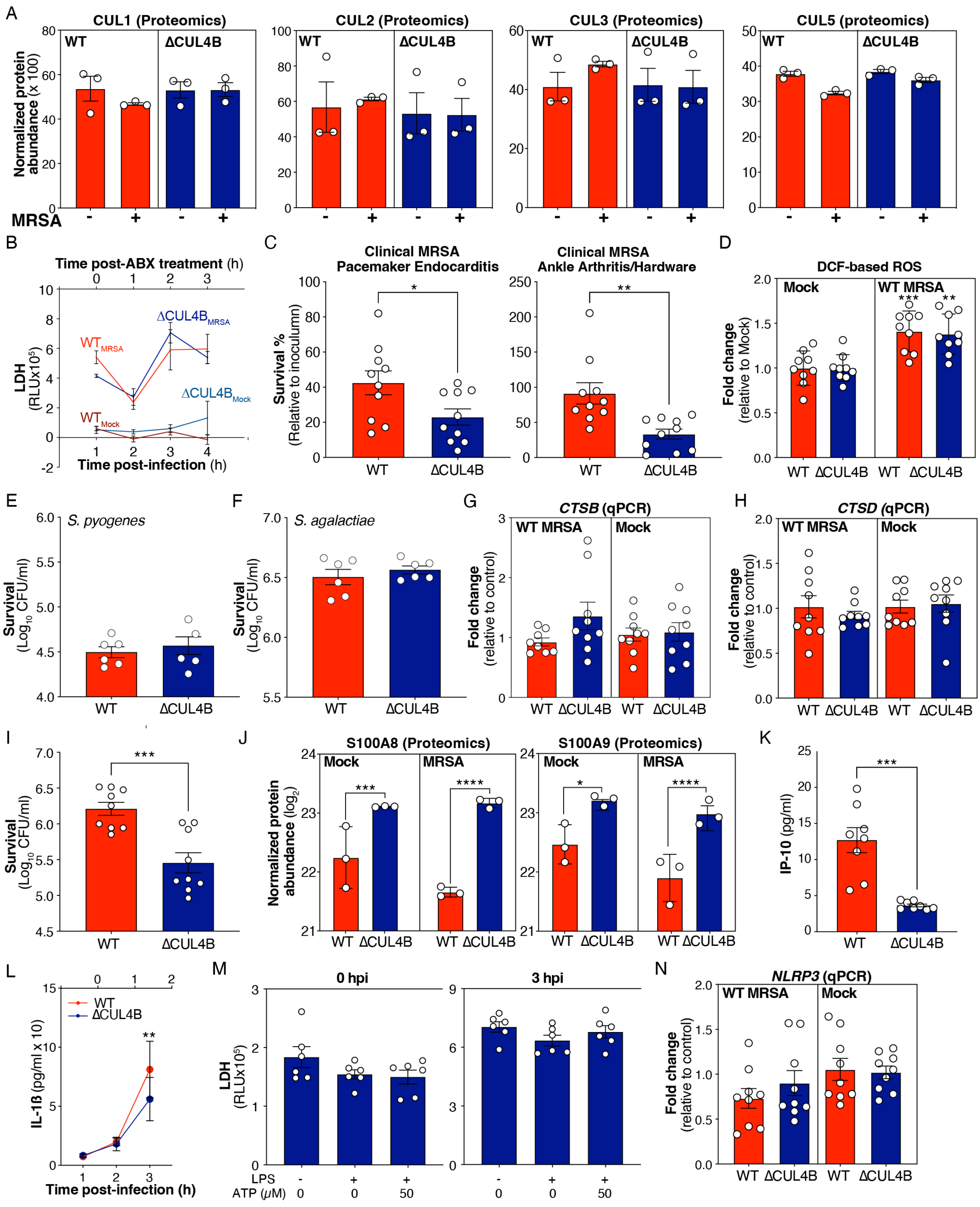

Figure S9. Transcription and translation of CUL4B and other cullins in MRSA-infected macrophages. (A) Protein abundance values of selected Cullins in MRSA- or mock-infected WT and ΔCUL4B MΦ cells. Cells were infected for 1 hour (MOI = 10), then washed and incubated in medium supplemented with gentamicin and lysostaphin (ABX treatment). The cells were lysed 4 hours post-infection (hpi) and subjected to LC-MS^2/MS^3-based quantitative proteomics. The data are plotted as mean normalized protein abundance ± SEM (Datasets S14–S15), representing three biological replicates, and were analyzed by Tukey's HS-test (post hoc) after an analysis of variance

(ANOVA) test (outlined in the Materials and Methods). **(B)** Time course analysis of WT and ΔCUL4B viability in response to MRSA. Differentiated MΦ cells (1 × 10^6 cells/well) were infected with MRSA for 1 hour (MOI = 10), then washed and incubated in medium supplemented with gentamicin and lysostaphin. Macrophage viability was assessed by measuring LDH release in the cell culture supernatant. The data are plotted as mean ± SEM, representing three experiments performed in duplicate, and were analyzed by two-way ANOVA (Tukey's multiple comparisons test). **(C)** Intracellular killing of MRSA clinical strains by WT and ΔCUL4B MΦ cells. Cells were infected (MOI = 10) as described in A. Intracellular MRSA was quantified by lysing the cells and enumerating the bacteria 3 hpi (or 2 h post-ABX treatment). Survival was calculated relative to the inoculum. The data are plotted as mean (percentage of survival) ± SEM, representing four experiments conducted in duplicate/triplicate, and were analyzed by an unpaired two-tailed *t*-test. Left panel: *P* = 0.0293; right panel: *P* = 0.0029. **(D)** DCF-based ROS assays were conducted to assess the impact of CUL4B on ROS production in MΦ cells. Differentiated THP-1 cells were left uninfected or infected with MRSA (MOI = 1) as described in A. ROS levels were quantified at 6 hpi (or 5 h post-ABX treatment) and expressed as the fold change of induction relative to mock-infected cells. The data are plotted as mean ± SEM, representing three experiments conducted in triplicate, and were analyzed by two-way ANOVA (Tukey's multiple comparisons test). WT (Mock vs. MRSA): *P* = 0.0008; ΔCUL4B (Mock vs. MRSA): *P* = 0.0019. **(E)** Intracellular killing of *S. pyogenes* by WT and ΔCUL4B MΦ cells. Cells were infected with *S. pyogenes* for 1 hour (MOI = 5), then washed and incubated in medium supplemented with gentamicin/penicillin. Intracellular bacteria were quantified by lysing the cells and enumerating the bacteria 4 hpi. The data are plotted as mean ± SEM, representing two experiments conducted in triplicate, and were analyzed by an unpaired two-tailed *t*-test. **(F)** Intracellular killing of *S. agalactiae* by WT and ΔCUL4B MΦ cells. Cells were infected with *S. agalactiae* for 1 hour (MOI = 10), then washed and incubated in medium supplemented with gentamicin/penicillin. Intracellular bacteria were quantified by lysing the cells and enumerating the bacteria 4 hpi. The data are plotted as mean ± SEM, representing two experiments conducted in triplicate, and were analyzed by an unpaired two-tailed *t*-test. **(G)** Relative mRNA expression of *CTSB* ($2^{-\Delta\Delta Cq}$) in MRSA- or mock-infected WT and ΔCUL4B MΦ cells. Cells were infected with WT MRSA for 1 hour (MOI = 10), then washed and incubated in medium supplemented with gentamicin and lysostaphin. The cells were subjected to qRT-PCR at 3 hpi. *GAPDH* was used as an endogenous control. The data are plotted as mean ± SEM, representing three experiments conducted in duplicate/triplicate, and were analyzed by and were analyzed by two-way ANOVA (Tukey's multiple comparisons test). **(H)** Relative mRNA expression of *CTSD* ($2^{-\Delta\Delta Cq}$) in MRSA- or mock-infected WT and ΔCUL4B MΦ cells. Cells were infected with WT MRSA as described in G and subjected to qRT-PCR at 3 hpi. *GAPDH* was used as an endogenous control. The data are plotted as mean ± SEM, representing three experiments conducted in duplicate/triplicate, and were analyzed by two-way ANOVA (Tukey's multiple comparisons test). **(I)** Intracellular killing of MRSA clinical strains by WT and ΔCUL4B MΦ cells. Cells were pretreated with bafilomycin A1 (1 µM, 90 minutes), followed by infection (MOI = 10) as described in A. Intracellular MRSA was quantified by lysing the cells and enumerating the bacteria 3 hpi (or 2 h post-ABX treatment). The data are plotted as mean ± SEM, representing three experiments conducted in triplicate, and were analyzed by an unpaired two-tailed *t*-test. Left panel: *P* = 0.0004. **(J)** Histogram showing the normalized S100A8 and S100A9 abundance ($\log_2$) in ΔCUL4B relative to WT MΦ cells under mock- and MRSA-infected conditions. Cells were infected with WT MRSA (MOI = 10, 3 hpi) as described in A and subjected to LC-MS^2/MS^3-based quantitative proteomics. The data are plotted as mean ± SEM, representing three biological replicates (Datasets S15–S16). The cutoff for significance was defined as FC ≥ 1.5 or FC ≤ −1.5, with an adjusted $P \leq 0.05$. S100A8: Mock: adj. *P-value = 0.0005,* MRSA: adj. *P-value* < 0.0001; S100A9: Mock: adj. *P-value = 0.00345*, MRSA: adj. *P-value* < 0.0001. **(K)** Quantification of IP-10 levels in the supernatants of MRSA-infected WT and ΔCUL4B cells 3 hpi using a multiplex assay. Cells were infected as described in A (MOI = 10, 3 hpi). The data are plotted as mean (pg/mL) ± SEM, representing three experiments conducted in triplicate/duplicate, and were analyzed by an unpaired two-tailed *t*-test. WT vs. ΔCUL4B: *P* = 0.0001. **(L)** Quantification of IL-1β levels in the supernatants of MRSA-infected WT ($CUL4B^{+/\Delta}$) and ΔCUL4B ($CUL4B^{\Delta/y}$) BMDMs using a multiplex

assay. Cells were infected with MRSA as described in A. The data are plotted as mean (pg/mL) ± SD, representing 3 mice/group, conducted in duplicate/triplicate, and analyzed by two-way ANOVA (Šídák's multiple comparisons). 3 hpi: *P* = 0.0012. **(M)** ΔCUL4B macrophages were primed with LPS for 3 h, followed by brief (0.5 h) low-dose ATP treatment. The cells were washed and infected with MRSA (MOI = 10, 3 hpi) as described in A. Macrophage viability was assessed by measuring LDH release in the cell culture supernatant both prior to (0 hpi, before washing) and post-MRSA infection (3 hpi). The data are plotted as mean ± SEM, representing two experiments performed in triplicate, and were analyzed by one-way ANOVA (Tukey's multiple comparisons). (**N)** Relative mRNA expression of *NLRP3* (2^-ΔΔCq) in MRSA- or mock-infected WT and ΔCUL4B MΦ cells. Cells were infected with WT MRSA as described in G and subjected to qRT-PCR at 3 hpi. *GAPDH* was used as an endogenous control. The data are plotted as mean ± SEM, representing three experiments conducted in duplicate/triplicate, and were analyzed by and were analyzed by two-way ANOVA (Tukey's multiple comparisons test). The significant differences are marked with asterisks (*): **P* ≤ 0.05; ***P* ≤ 0.01; ****P* ≤ 0.001; *****P* ≤ 0.0001.

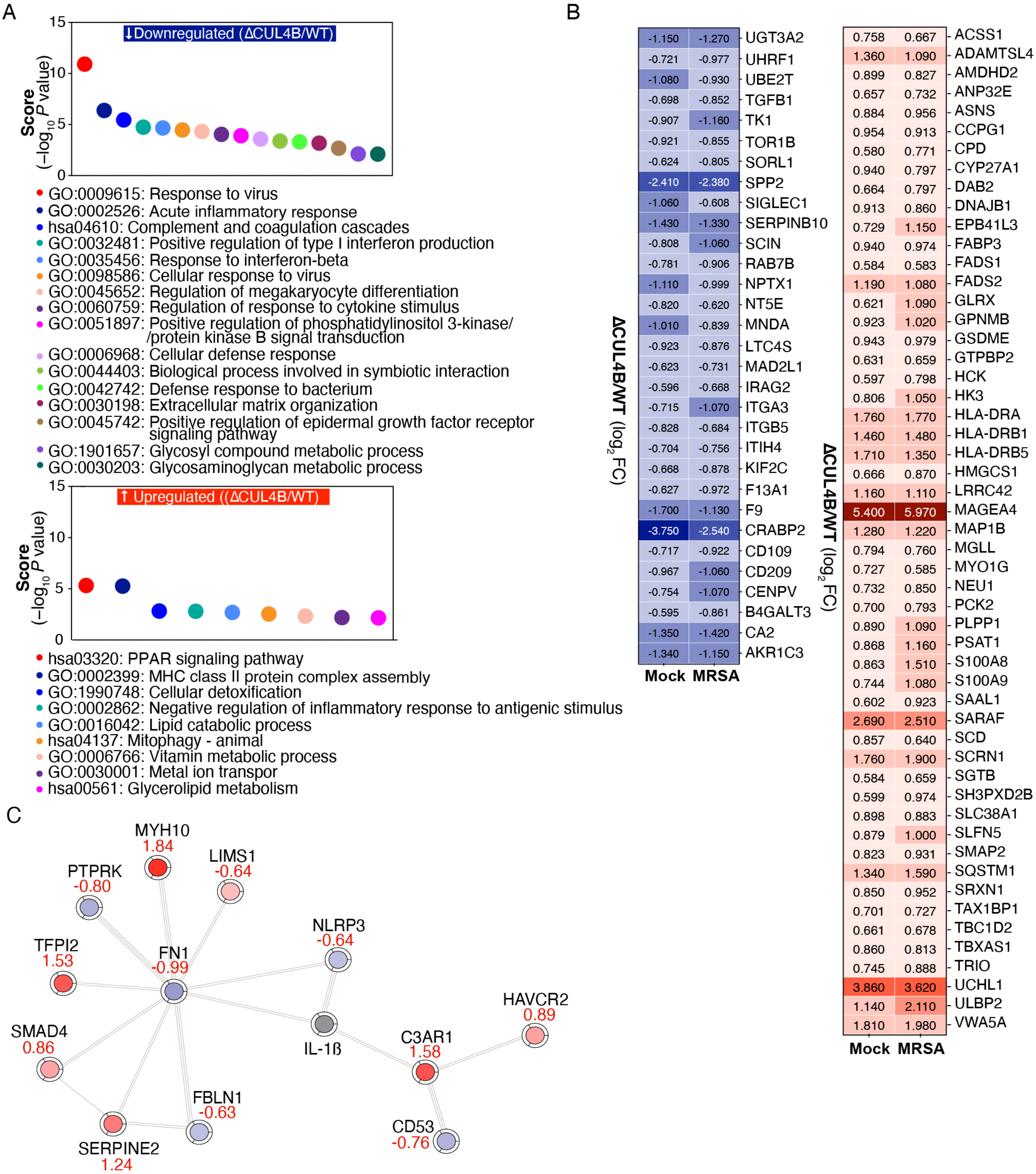

Figure S10. The proteomic response to *CUL4B* deletion in mock- and MRSA-infected THP-1 derived macrophages. **(A)** A dot plot of enrichment scores ($-\log_{10}$ *P*-value ≥ 2, *P*-value cutoff = 0.01) displays pathways and cellular processes enriched in the proteome of mock-infected ΔCUL4B compared to WT MΦ cells (Dataset S16). Enrichment analysis was performed using the list of significantly regulated proteins (FC ≥ 1.5 or FC ≤ −1.5; adjusted *P* ≤ 0.05) by Metascape (*121*); the *P*-value was calculated based on a cumulative hypergeometric distribution. **(B)** Heatmap showing the average fold change values ($\log_2$) for significantly regulated proteins (FC ≥ 1.5 or FC ≤ −1.5; adjusted *P* ≤ 0.05) belonging to the shared proteome in mock- and MRSA-infected ΔCUL4B relative to WT MΦ cells (Dataset S16). **(C)** Protein–protein interaction networks were constructed using STRING for the significantly regulated proteins associated with cell death (FC ≥ 1.5 or FC ≤ −1.5; adjusted *P* ≤ 0.05), as listed in Fig. 3A. The average fold change values ($\log_2$) were also

visualized on the nodes using a blue–white–red gradient. The IL-1β node incorporated into the network by the STRING algorithm is marked in grey. Singletons were excluded from the visualization.

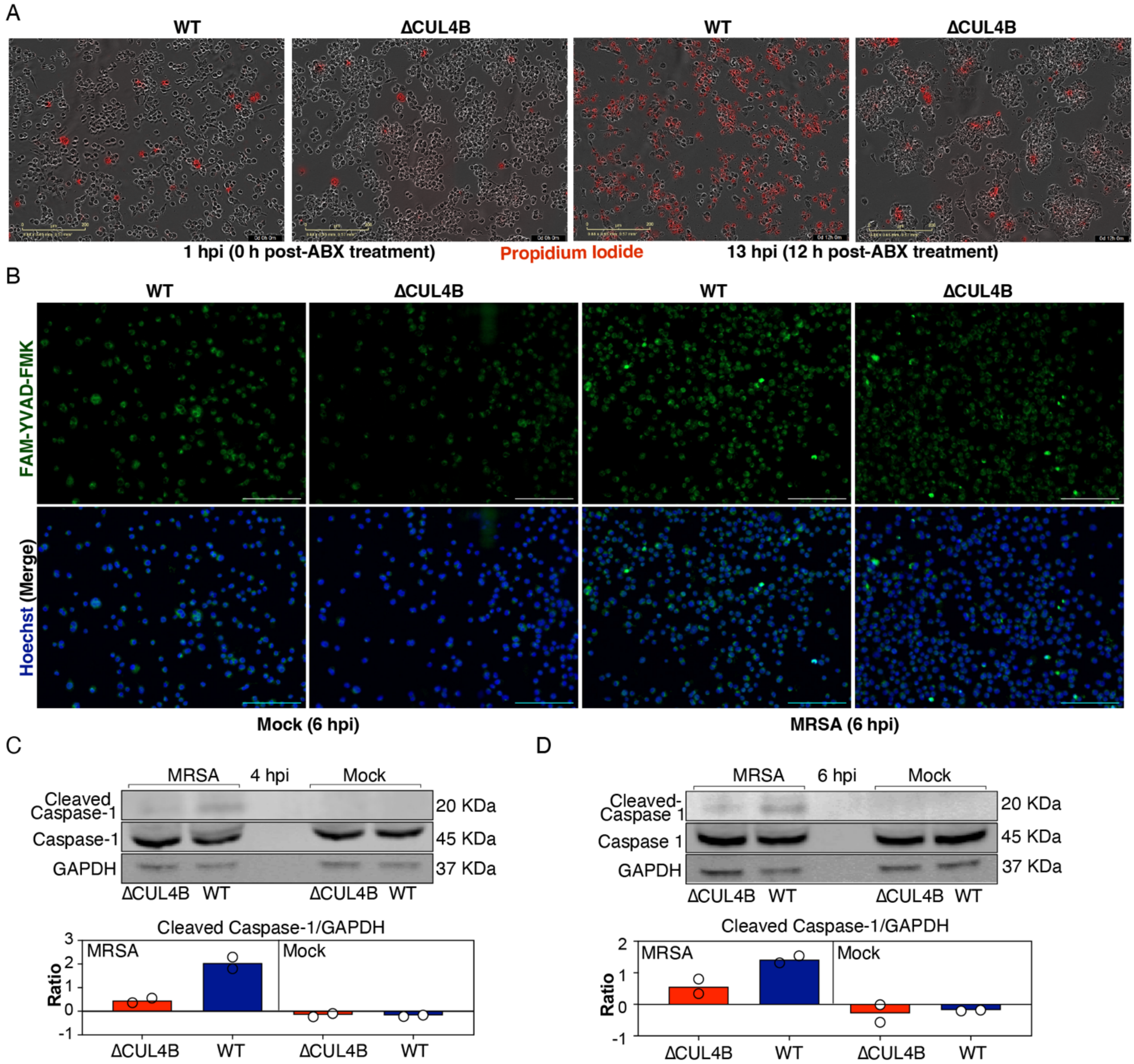

Figure S11. Deletion of CUL4B alleviates cell death in MRSA-infected macrophages. **(A)** Representative images of real-time viability analysis of MRSA- or mock-infected WT and ΔCUL4B MΦ cells at 1 and 13 hours post-infection (hpi). Differentiated THP-1 cells were infected with MRSA (MOI = 2). One hour post-infection, cells were washed and incubated in medium supplemented with gentamicin and lysostaphin. The cells were then transferred to the IncuCyte, where they were incubated with medium containing gentamicin, lysostaphin, and propidium iodide (PI). Images were captured by IncuCyte Zoom with a 10× objective (scale bar = 200 µm). **(B)** Representative images of activated caspase-1 in MRSA- or mock-infected WT and ΔCUL4B macrophages (MOI = 2.5, 6 hpi). One hour post-infection, cells were washed and incubated in medium supplemented with gentamicin and lysostaphin. The cells were stained with FAM-FLICA caspase-1 (FAM-YVAD-FMK) and Hoechst, and analyzed by fluorescence microscopy (20× magnification, scale bar = 100 µm). **(C–D)** Representative images of immunoblot analyses of MRSA- or mock-infected WT and ΔCUL4B MΦ cells at (C) 4 hpi and (D) 6 hpi. Differentiated THP-1 cells were infected with MRSA (MOI = 10), as described in A. Combined cell lysates and supernatants were precipitated using the methanol/chloroform method and subjected to immunoblot analysis to detect cleaved caspase-1 (p20), procaspase-1, and GAPDH. The ratio of cleaved caspase-1 (p20) to GAPDH was quantified by densitometric analysis using Image Studio on two independent membranes, each corresponding to a separate biological replicate. The uncropped immunoblot is shown in fig. S16.

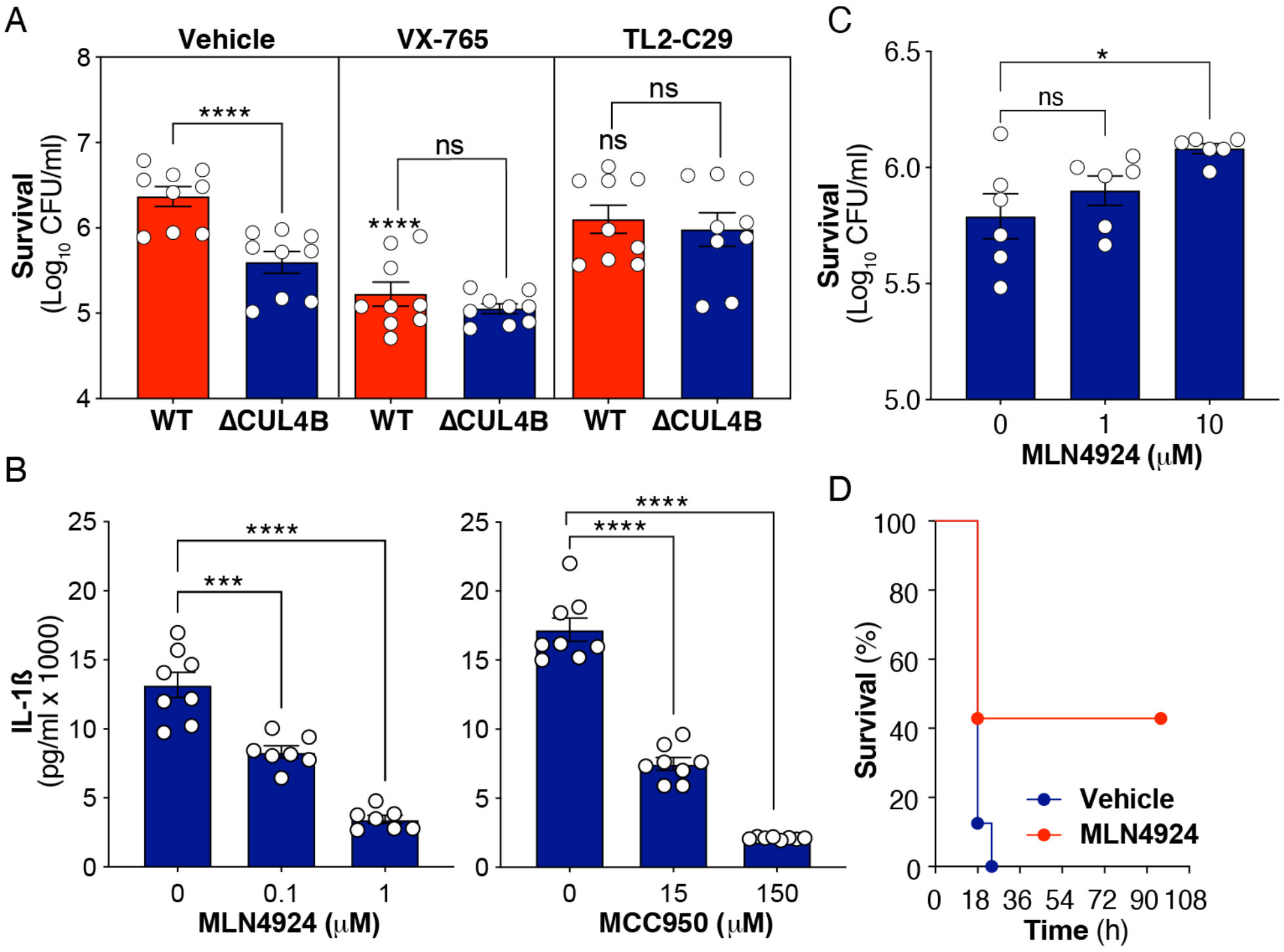

Figure S12. Pharmacological inhibition of inflammasome signaling and cullin neddylation during MRSA infection. (A) WT and ΔCUL4B macrophages were pretreated with VX-765 and TL2-C29 and infected with MRSA (MOI = 10). One hour post-infection, cells were washed and incubated in medium supplemented with gentamicin and lysostaphin. Intracellular MRSA was quantified (3 hpi) by lysing the cells and enumerating the bacteria. The data are plotted as the mean ± SEM, representing three experiments performed in triplicate, and were analyzed by two-way ANOVA (Tukey's multiple comparisons). Vehicle: $P \leq 0.001$ (WT vs. ΔCUL4B); VX-765: $P \leq 0.001$ (WT vs. ΔCUL4B; $WT_{Vehicle}$ vs. $WT_{VX\text{-}765}$). **(B)** Quantification of IL-1β levels in the supernatants of MRSA-infected WT MΦ cells at 3 hpi. Cells were pretreated with increasing concentrations of MCC950 and MLN4924 and infected with MRSA (MOI = 10) as described in A. The data are plotted as mean (pg/mL) ± SEM, representing three experiments conducted in duplicate/triplicate, and analyzed by one-way ANOVA (Tukey's multiple comparisons test). MCC950: $P \leq 0.001$ (0 vs. 15 μM; 0 vs. 150); MLN4924: $P = 0.0001$ (0 vs. 10 μM), $P \leq 0.001$ (0 vs. 1 μM). **(C)** Survival of MRSA following 30 minutes of incubation with freshly isolated human neutrophils pretreated with increasing concentrations of MLN4924. The data are plotted as mean ± SEM, representing two experiments conducted in triplicate, and were analyzed by one-way ANOVA (Tukey's multiple comparisons test). MRSA: $P = 0.0217$ (0 vs. 10 μM). **(D)** Female 8-week-old C57BL/6J mice (Vehicle: n = 8/group; MLN4924: n = 7/group) were inoculated IT with 1.8×10^8 CFU MRSA per mouse. Survival is shown using Kaplan–Meier survival curves and was analyzed using the log-rank (Mantel–Cox) test. $P = 0.055$. The significant differences are marked with asterisks (*): *$P \leq 0.05$; ***$P \leq 0.001$; ****$P \leq 0.0001$; ns = non-significant ($P > 0.05$).

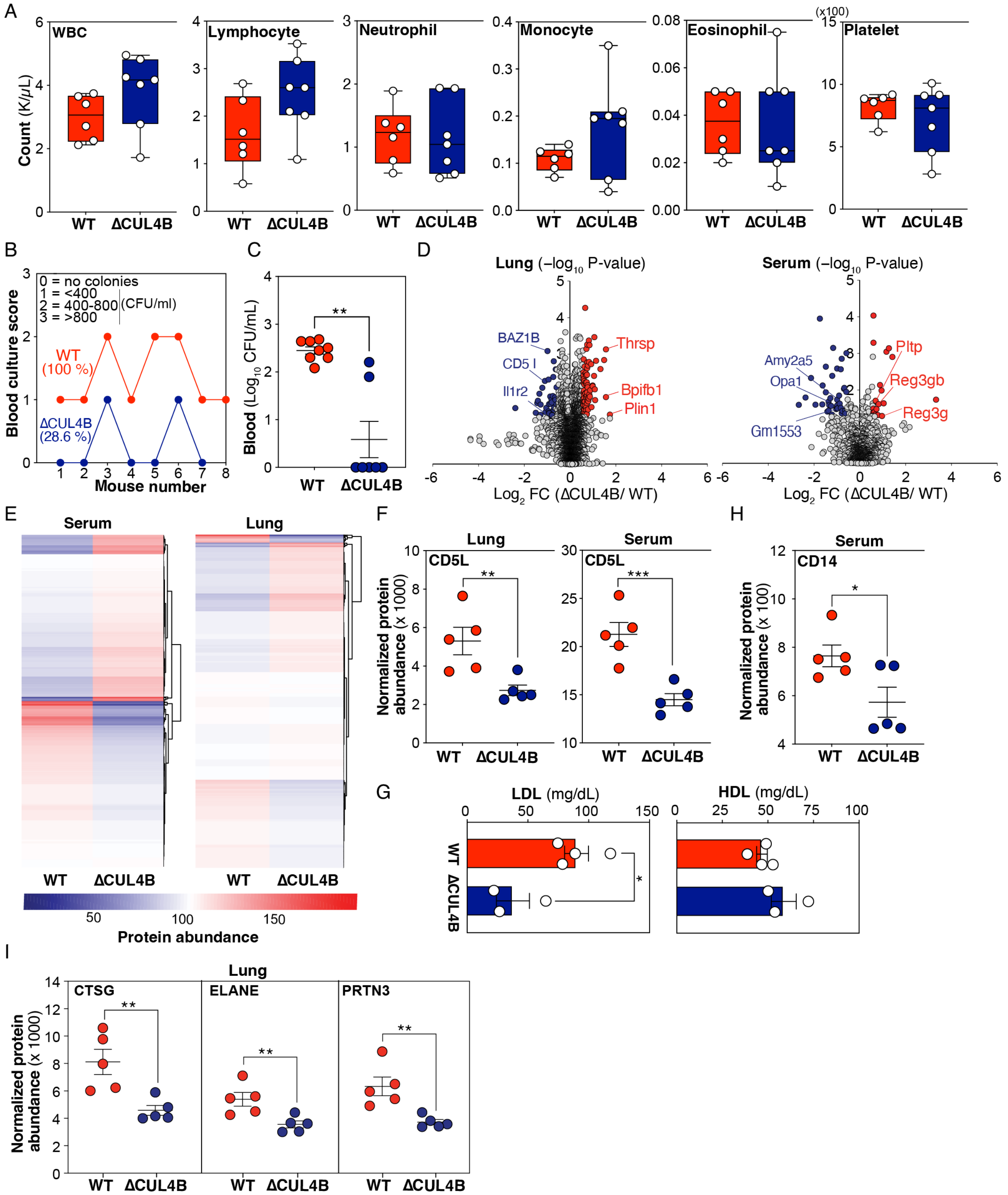

Figure S13. CUL4B deficiency promotes MRSA clearance *in vivo*. (A) Male wild-type (n = 8) and ΔCUL4B (CUL4B$^{\Delta/y}$, n = 7) C57BL/6J mice were inoculated IT with 5 × 10^7 CFU MRSA WT per mouse. Blood cell counts were performed 24 hpi. Data are plotted as box plots and were analyzed by a two-tailed *t*-test. The box represents the interquartile interval (25%–75%), with the horizontal line in the box indicating the median. Whiskers correspond to the minimum and maximum values. **(B)** WT (n = 8) and ΔCUL4B (n = 7) mice were intratracheally challenged with MRSA (as detailed in A), and blood cultures were assessed 24 hours post-infection. Mice were scored based on bacterial load in blood. The percentage of mice with positive blood cultures is provided within the figure. **(C)** WT (n = 8) and ΔCUL4B (n = 7) mice were intratracheally challenged

with MRSA (as detailed in A). Bacterial loads in blood (CFU/mL) were enumerated 24 hpi. Data are plotted as the mean ± SEM, representing 7–8 mice per group, and were analyzed by a two-tailed *t*-test. $P = 0.0024$. **(D)** Volcano plots showing differentially expressed proteins in the lung (left panel) and serum (right panel) proteomes of intratracheally MRSA-infected ΔCUL4B relative to WT mice. Significance cutoffs were $P = 0.05$ ($-\log_{10} = 1.3$) and fold change (FC) ≥ 1.5 or FC ≤ −1.5. Significance was determined using a two-tailed paired *t*-test, and significantly up- or downregulated proteins are marked in red and blue, respectively. **(E)** Euclidean hierarchical clustering analysis of normalized protein abundances in the lung and serum of intratracheally infected ΔCUL4B and WT mice (as described in A, n = 5 mice/group). **(F)** Protein abundance values of CD5L (CD5 molecule-like) in serum and lung proteomes of intratracheally challenged WT and ΔCUL4B mice (as described in A, n = 5 mice/group) (Datasets S19 and S21). Serum: $P = 0.0053$; Lung: $P = 0.0009$. **(G)** LDL- and HDL-cholesterol levels in the serum of intratracheally challenged ΔCUL4B (n = 3) and WT (n = 4) mice 24 hpi (as described in A). Data are plotted as mean ± SEM, representing 3–4 mice per group, and were analyzed by a two-tailed *t*-test. LDL: $P = 0.0234$. **(H)** Protein abundance of CD14 (monocyte differentiation antigen CD14) in serum proteome of intratracheally challenged WT and ΔCUL4B mice (as described in A, n = 5 mice/group) (Dataset S19). $P = 0.043$. **(I)** Protein abundance values of CTSG (cathepsin G), ELANE (neutrophil elastase), and PRTN3 (myeloblastin) in lung proteome of intratracheally challenged WT and ΔCUL4B mice (as described in A, n = 5 mice/group) (Dataset S21). CTSG: $P = 0.0049$; ELANE: $P = 0.0080$; PRTN3: $P = 0.0041$. The significant differences are marked with asterisks (*): *$P \leq 0.05$; **$P \leq 0.01$; ***$P \leq 0.001$.

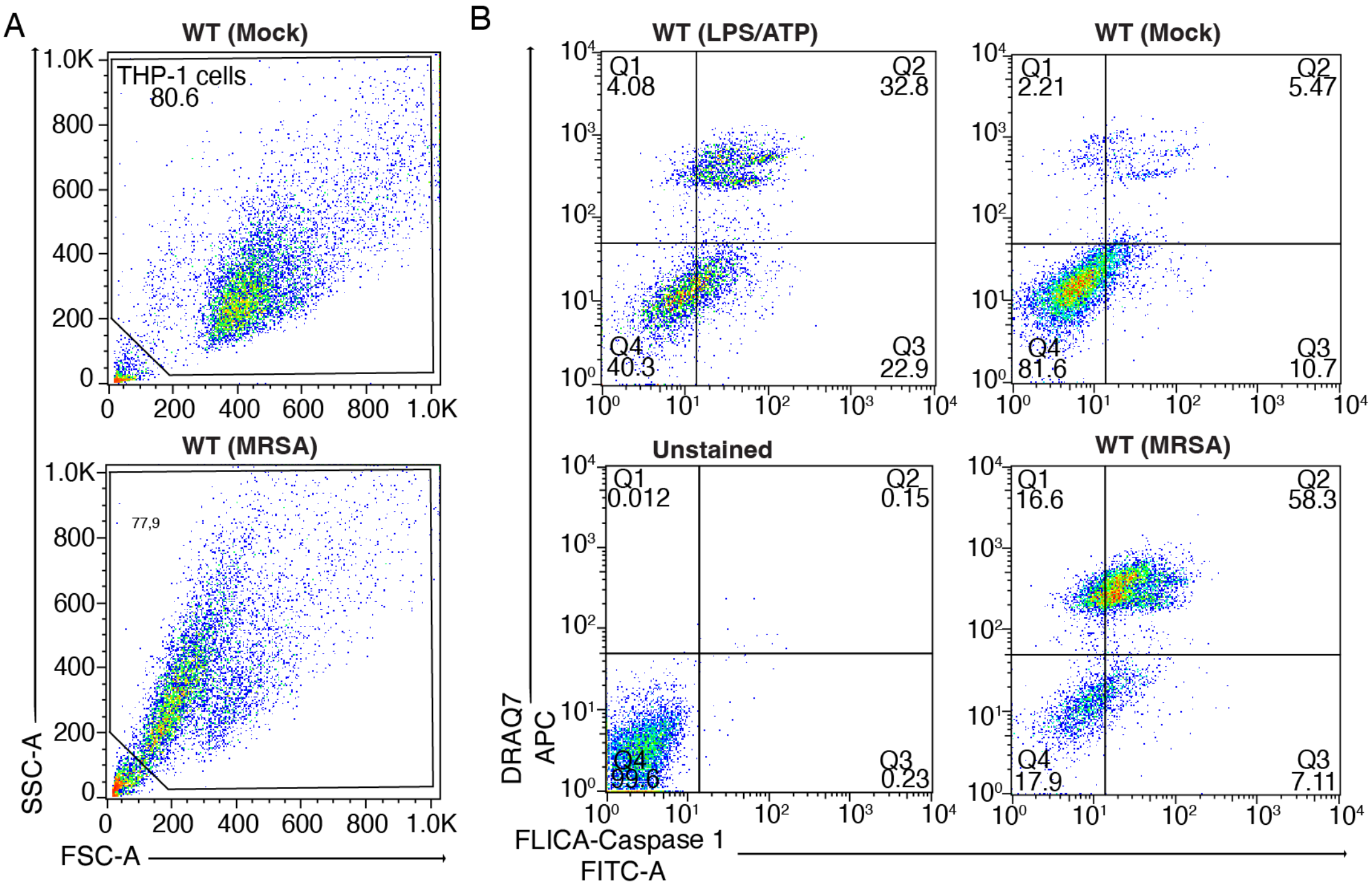

Figure S14. The representative flow cytometry plots illustrate the gating strategy used to quantify activated caspase-1. MRSA- or mock-infected ΔCUL4B and WT cells were left unstained or stained with FAM-FLICA caspase-1 (FAM-YVAD-FMK) and DRAQ7 (live/dead) reagents and analyzed using flow cytometry. The data were processed with FlowJo, and **(A)** cells were gated on side scatter (SSC) versus forward scatter (FSC). Subsequently, cells were gated based on live (APC^-) or dead (APC^+) status, as well as the FLICA–caspase-1 positive ($FITC^+$) and negative ($FITC^-$) markers. **(B)** Flow plots are shown for LPS/ATP-treated cells (positive control), unstained mock- and MRSA-infected cells. The percentage of cells in the FLICA–caspase-1^+ (Q2+Q3) population was used to quantify cells with activated caspase-1 in response to MRSA infection.

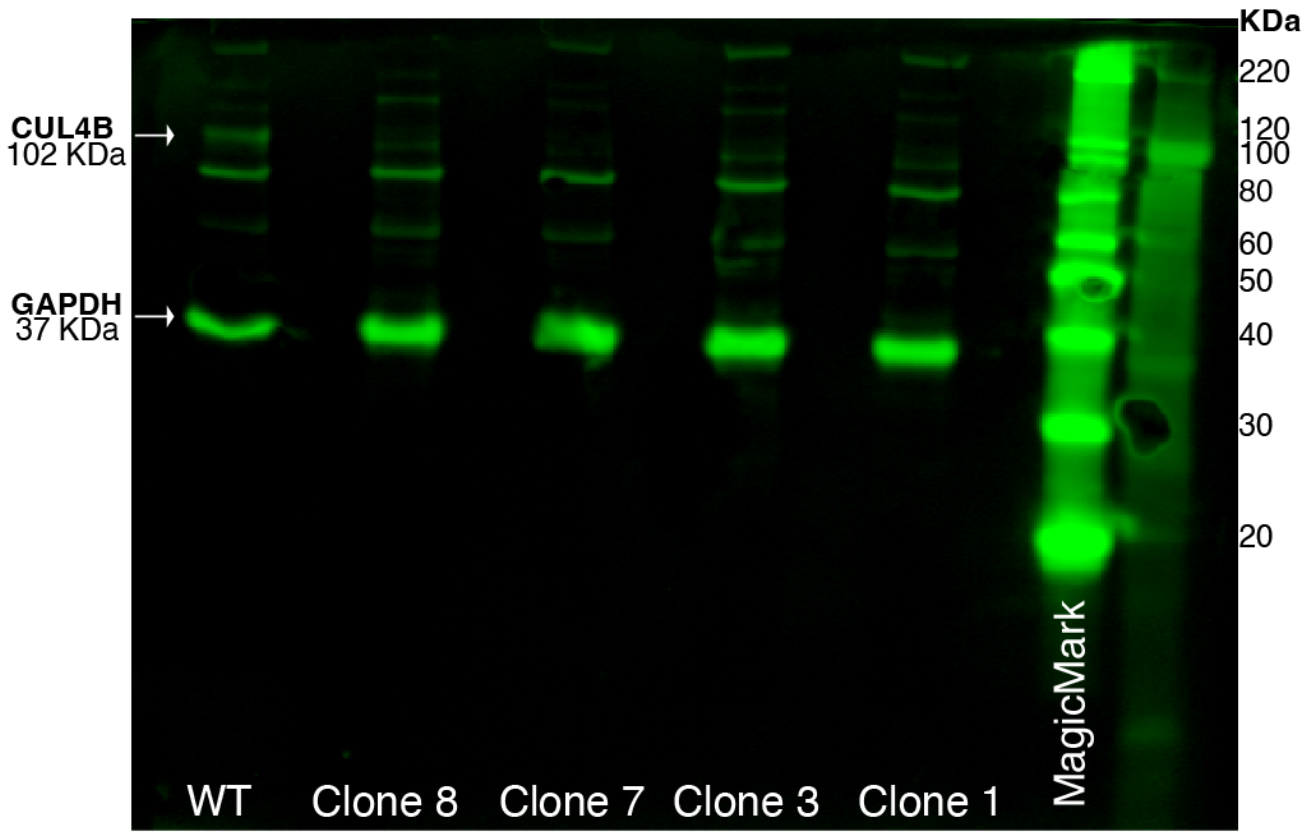

Figure S15. The genetically confirmed ΔCUL4B THP-1 clones were verified by immunoblotting for CUL4B expression. WT THP-1 cells served as a positive control. GAPDH was used as a loading control. MagicMark™ XP western protein standard was used as a molecular weight marker. The cropped western blot is shown in fig. S8D.

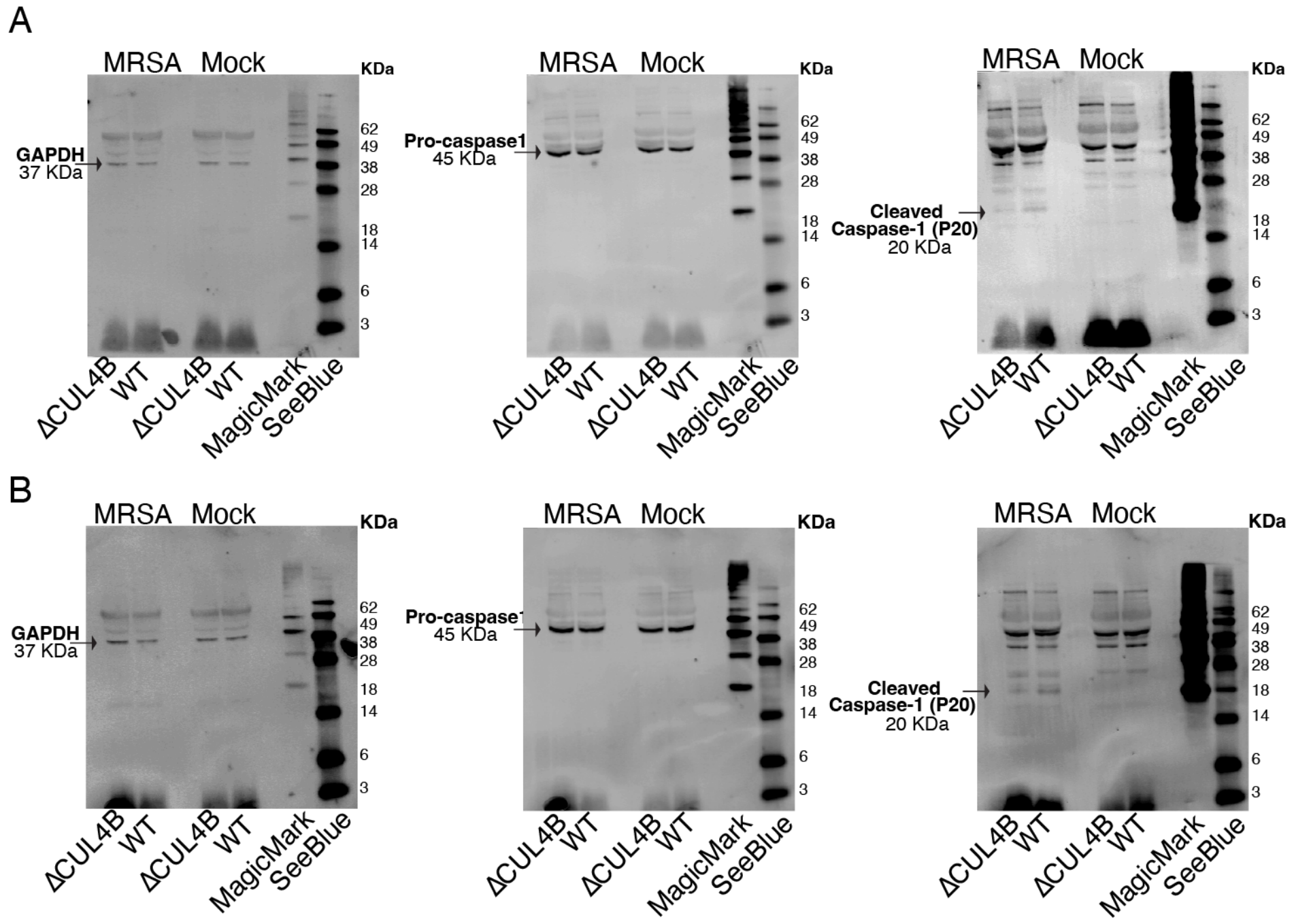

Figure S16. Uncropped immunoblots of caspase-1 processing in MRSA- or mock-infected WT and ΔCUL4B MΦ cells. Combined cell lysates and supernatants were collected at **(A)** 4 hours and **(B)** 6 hours post-infection (hpi). The samples were precipitated using the methanol/chloroform method and subjected to immunoblot analysis to detect cleaved caspase-1 (p20), procaspase-1, and GAPDH. MagicMark™ XP and SeeBlue™ protein standards were used as molecular weight markers. The cropped immunoblots are shown in fig. S11C-D.

Table S1. Primers used in this study

Primer	Sequence (5′–3′)	Origin
IL-1B$_{FW}$	TCTGTACCTGTCCTGCGTGT	(*172*)
IL-1B$_{RV}$	ACTGGGCAGACTCAAATTCC	(*172*)
TNF$_{FW}$	CAGCCTCTTCTCCTTCCTGAT	(*172*)
TNF$_{RV}$	GCCAGAGGGCTGATTAGAGA	(*172*)
CUL4B$_{FW}$	ACTCCTCCTTTACAACCCAGG	(*173*)
CUL4B$_{RV}$	TCTTCGCATCAAACCCTACAAAC	(*173*)
CTSL$_{FW}$	GAAAGGCTACGTGACTCCTGTG	OriGene
CTSL$_{RV}$	CCAGATTCTGCTCACTCAGTGAG	OriGene
CTSB$_{FW}$	GCTTCGATGCACGGGAACAATG	OriGene
CTSB$_{RV}$	CATTGGTGTGGATGCAGATCCG	OriGene
CTSD$_{FW}$	GCAAACTGCTGGACATCGCTTG	OriGene
CTSD$_{RV}$	GCCATAGTGGATGTCAAACGAGG	OriGene
NLRP3$_{FW}$	GATCTTCGCTGCGATCAACAG	(*174*)
NLRP3$_{RV}$	CGTGCATTATCTGAACCCCAC	(*174*)
GAPDH$_{FW}$	AGGTCGGTGTGAACGGATTTG	(*175*)
GAPDH$_{RV}$	TGTAGACCATGTAGTTGAGGTCA	(*175*)

Datasets legends

Dataset S1. A preliminary proteomic dataset from MΦ cells in response to MRSA at multiple time points (1, 2, 4, 7, and 24 hours) post-infection. The results were obtained from two/three biological replicates.

Dataset S2. A list of differentially regulated proteins and their corresponding *P*-values, identified by comparing MRSA-infected with mock-infected MΦ cells. The significance thresholds were set to a fold change (FC) ≥ 1.5 or FC ≤ −1.5 and an adjusted *P* ≤ 0.05 ($-\log_{10} = 1.3$). The results were obtained from two/three biological replicates.

Dataset S3. A list of differentially regulated proteins categorized as shared (common across all time points) or unique proteomes (specific to individual time points) in MRSA-infected compared with mock-infected MΦ cells. The list includes differentially regulated proteins from Dataset S2.

Dataset S4. A preliminary transcriptomic dataset from MΦ cells in response to MRSA at two time points (7 and 24 hours) post-infection. The results were obtained from two biological replicates.

Dataset S5. A list of differentially regulated genes and their corresponding *P*-values identified by comparing MRSA-infected with mock-infected MΦ transcriptomes. The significance thresholds were set to a fold change (FC) ≥ 2 or FC ≤ −2 and an adjusted *P* ≤ 0.05. The results were obtained from two biological replicates.

Dataset S6. A list of differentially regulated genes categorized as shared or unique transcriptomes (specific to individual time points) in MRSA-infected compared with mock-infected MΦ cells. For the shared differentially regulated genes, the corresponding upregulated and downregulated expression statistics are provided in separate worksheets for the 7 hpi and 24 hpi datasets, respectively. The list includes differentially regulated genes from Dataset S5.

Dataset S7. A list of predicted upstream regulators derived from IPA analysis based on the list of differentially regulated proteins in Dataset S2, is categorized as either shared (common across all time points) or unique (specific to individual time points) regulators in MRSA-infected MΦ cells. The significance thresholds were set to a z-score (activated) ≥ 2 or z-score ≤ −2 (inhibited) and a network bias-corrected *P*-values ≤ 0.05.

Dataset S8. A list of the average fold change values ($\log_2$) and *P*-values for significantly dysregulated genes (FC ≥ 2 or FC ≤ −2, adjusted *P* ≤ 0.05) and proteins (FC ≥ 1.5 or FC ≤ −1.5, adjusted all *P* ≤ 0.05) at one or more time points in MRSA-infected MΦ cells compared with mock-infected controls, linked to the shared regulators (Rock, Rac, ATM, CUL4B, Pkg, MYC, AREG). The list includes differentially and non-differentially regulated proteins/genes from Datasets S1–S2 and S4–S5.

Dataset S9. A list of the average fold change values ($\log_2$) and *P*-values for significantly dysregulated genes (FC ≥ 2or FC ≤ −2, adjusted *P* ≤ 0.05) and proteins (FC ≥ 1.5 or FC ≤ −1.5, adjusted all *P* ≤ 0.05) shared across all regulators from Dataset 11 at one or more time points in MRSA-infected MΦ cells compared with mock-infected controls. The list includes differentially and non-differentially regulated proteins/genes from Datasets S1–S2, S4–S5, and S8.

Dataset S10. A list of the average fold change values ($\log_2$) and *P*-values for significantly dysregulated (FC ≥ 1.5 or FC ≤ −1.5) genes (adjusted *P* ≤ 0.05) and proteins (adjusted all *P* ≤ 0.05) at one or more time points in MRSA-infected MΦ cells compared with Mock-infected controls, linked to CUL4B. The list includes differentially and non-differentially regulated proteins/genes from Datasets S1–2, S4–5, and S8.

Dataset S11. A list of the significantly altered IPA canonical pathways in the proteome of MRSA-infected MΦ cells. The significance thresholds were set to a z-score (activated) ≥ 2 or z-score ≤ −2 (inhibited) and *P* ≤ 0.05. Enrichment analysis was performed using the list of significantly dysregulated proteins (Dataset S2) in MRSA-infected MΦ cells compared with mock-infected controls (FC ≥ 1.5 or FC ≤ −1.5 and adjusted *P* ≤ 0.05).

Dataset S12. A preliminary secretome dataset from cell culture supernatants of ΔCUL4B and WT MΦ cells in response to mock infection or internalized MRSA (MOI = 10) at 4 hpi. The results were obtained from three biological replicates.

Dataset S13. A list of differentially regulated proteins in the secretome and their corresponding *P*-values, identified by comparing the ΔCUL4B with WT MΦ cells in response to mock infection or internalized MRSA at 4 hpi. The significance thresholds were set to FC ≥ 1.5 or FC ≤ −1.5 and *P* ≤ 0.05.

Dataset S14. A list of identified Cullin-RING E3 Ubiquitin Ligases in the proteome (cell lysate) and their corresponding normalized abundance and fold change values (FC), obtained by comparing ΔCUL4B with WT MΦ cells in response to mock infection or internalized MRSA (MOI = 10) at 4 hpi. The results were obtained from three biological replicates.

Dataset S15. A preliminary cohort of proteomic dataset (cell lysate proteome) from ΔCUL4B and WT MΦ cells in response to mock infection or internalized MRSA at 3 hpi. The results were obtained from three biological replicates and presented as Log_2 protein abundance.

Dataset S16. A list of differentially regulated proteins in the proteome (cell lysate) and their corresponding *P*-values, identified by comparing the ΔCUL4B with WT MΦ cells in response to mock infection or internalized MRSA at 3 hpi. The significance thresholds were set to FC ≥ 1.5 or FC ≤ −1.5 and adjusted *P* ≤ 0.05. The results were obtained from three biological replicates and presented as Log_2 protein abundance.

Dataset S17. NLRP3 abundance (Log_2) in the proteome (cell lysate) and its corresponding fold change values (FC), *P*-value, and adjusted *P*-value, identified by comparing the ΔCUL4B with WT MΦ cells in response to mock infection or internalized MRSA in the presence and absence of the inhibitors (CHX, MG132) at 3 hpi. The results were obtained from three biological replicates, and the significance thresholds were set to FC ≥ 1.5 or FC ≤ −1.5 and adjusted *P* ≤ 0.05.

Dataset S18. A preliminary serum proteomic dataset from ΔCUL4B and WT mice infected intratracheally with MRSA and analyzed at 24 hpi. The results were obtained from 5 mice per group.

Dataset S19. A list of differentially regulated proteins in the serum proteome and their corresponding *P*-values, identified by comparing infected ΔCUL4B with WT infected mice. The significance thresholds were set to FC ≥ 1.5 or FC ≤ −1.5 and *P* ≤ 0.05. The results were obtained from 5 mice per group.

Dataset S20. A preliminary lung proteomic dataset from ΔCUL4B and WT mice infected intratracheally with MRSA and analyzed at 24 hpi. The results were obtained from 5 mice per group.

Dataset S21. A list of differentially regulated proteins in the lung proteome and their corresponding *P*-values, identified by comparing infected ΔCUL4B with WT infected mice. The significance thresholds were set to FC ≥ 1.5 or FC ≤ −1.5 and P ≤ 0.05. The results were obtained from 5 mice per group.

REFERENCES

1. T. J. Foster, J. A. Geoghegan, V. K. Ganesh, M. Hook, Adhesion, invasion and evasion: The many functions of the surface proteins of *Staphylococcus aureus*. *Nat. Rev. Microbiol.* **12**, 49–62 (2014).

2. H. F. Chambers, F. R. Deleo, Waves of resistance: *Staphylococcus aureus* in the antibiotic era. *Nat. Rev. Microbiol.* **7**, 629–641 (2009).

3. A. N. Spaan, B. G. Surewaard, R. Nijland, J. A. van Strijp, Neutrophils versus *Staphylococcus aureus*: A biological tug of war. *Annu. Rev. Microbiol.* **67**, 629–650 (2013).

4. R. S. Flannagan, B. Heit, D. E. Heinrichs, Antimicrobial mechanisms of macrophages and the immune evasion strategies of *Staphylococcus aureus*. *Pathogens* **4**, 826–868 (2015).

5. B. P. Howden, S. G. Giulieri, T. W. F. Lung, S. L. Baines, L. K. Sharkey, J. Y. H. Lee, A. Hachani, I. R. Monk, T. P. Stinear, *Staphylococcus aureus* host interactions and adaptation. *Nat. Rev. Microbiol.* **21**, 380–395 (2023).

6. M. M. Dinges, P. M. Orwin, P. M. Schlievert, Exotoxins of *Staphylococcus aureus*. *Clin. Microbiol. Rev.* **13**, 16–34 (2000).

7. S. H. E. Kaufmann, A. Dorhoi, Molecular determinants in phagocyte-bacteria interactions. *Immunity* **44**, 476–491 (2016).

8. S. Arandjelovic, K. S. Ravichandran, Phagocytosis of apoptotic cells in homeostasis. *Nat. Immunol.* **16**, 907–917 (2015).

9. V. Thammavongsa, H. K. Kim, D. Missiakas, O. Schneewind, Staphylococcal manipulation of host immune responses. *Nat. Rev. Microbiol.* **13**, 529–543 (2015).

10. M. Kubica, K. Guzik, J. Koziel, M. Zarebski, W. Richter, B. Gajkowska, A. Golda, A. Maciag-Gudowska, K. Brix, L. Shaw, T. Foster, J. Potempa, A potential new pathway for *Staphylococcus aureus* Dissemination: The silent survival of *S. aureus* phagocytosed by human monocyte-derived macrophages. *PLOS ONE* **3**, e1409 (2008).

11. M. Grosz, J. Kolter, K. Paprotka, A. C. Winkler, D. Schafer, S. S. Chatterjee, T. Geiger, C. Wolz, K. Ohlsen, M. Otto, T. Rudel, B. Sinha, M. Fraunholz, Cytoplasmic replication of *Staphylococcus aureus* upon phagosomal escape triggered by phenol-soluble modulin α. *Cell. Microbiol.* **16**, 451–465 (2014).

12. L. Michailova, S. Stoitsova, N. Markova, V. Kussovski, M. Jordanova, I. Dimova, Interaction of alveolar macrophages with *Staphylococcus aureus* and induction of microbial L-forms during infection in rats. *Int. J. Med. Microbiol.* **290**, 259–267 (2000).

13. G. J. Jakab, G. M. Green, Defect in intracellular killing of *Staphylococcus aureus* within alveolar macrophages in Sendai virus-infected murine lungs. *J. Clin. Invest.* **57**, 1533–1539 (1976).

14. Z. R. Tranchemontagne, R. B. Camire, V. J. O'Donnell, J. Baugh, K. M. Burkholder, *Staphylococcus aureus* strain USA300 perturbs acquisition of lysosomal enzymes and requires phagosomal acidification for survival inside macrophages. *Infect. Immun.* **84**, 241–253 (2015).

15. R. S. Flannagan, B. Heit, D. E. Heinrichs, Intracellular replication of *Staphylococcus aureus* in mature phagolysosomes in macrophages precedes host cell death, and bacterial escape and dissemination. *Cell. Microbiol.* **18**, 514–535 (2016).

16. P. Sendi, R. A. Proctor, *Staphylococcus aureus* as an intracellular pathogen: The role of small colony variants. *Trends Microbiol.* **17**, 54–58 (2009).

17. C. A. Petti, V. G. Fowler Jr., *Staphylococcus aureus* bacteremia and endocarditis. *Cardiol. Clin.* **21**, 219–233 (2003).

18. G. R. Pidwill, J. F. Gibson, J. Cole, S. A. Renshaw, S. J. Foster, The role of macrophages in *Staphylococcus aureus* infection. *Front. Immunol.* **11**, 620339 (2020).

19. D. Missiakas, V. Winstel, Selective host cell death by *Staphylococcus aureus*: A strategy for bacterial persistence. *Front. Immunol.* **11**, 621733 (2020).

20. R. R. Craven, X. Gao, I. C. Allen, D. Gris, J. Bubeck Wardenburg, E. McElvania-Tekippe, J. P. Ting, J. A. Duncan, *Staphylococcus aureus* α-hemolysin activates the NLRP3-inflammasome in human and mouse monocytic cells. *PLOS ONE* **4**, e7446 (2009).

21. R. Muñoz-Planillo, L. Franchi, L. S. Miller, G. Núñez, A critical role for hemolysins and bacterial lipoproteins in *Staphylococcus aureus*-induced activation of the Nlrp3 inflammasome. *J. Immunol.* **183**, 3942–3948 (2009).

22. D. Holzinger, L. Gieldon, V. Mysore, N. Nippe, D. J. Taxman, J. A. Duncan, P. M. Broglie, K. Marketon, J. Austermann, T. Vogl, D. Foell, S. Niemann, G. Peters, J. Roth, B. Löffler, *Staphylococcus aureus* Panton-Valentine leukocidin induces an inflammatory response in human phagocytes via the NLRP3 inflammasome. *J. Leukoc. Biol.* **92**, 1069–1081 (2012).

23. R. Muñoz-Planillo, P. Kuffa, G. Martínez-Colón, B. L. Smith, T. M. Rajendiran, G. Núñez, K^+ efflux is the common trigger of NLRP3 inflammasome activation by bacterial toxins and particulate matter. *Immunity* **38**, 1142–1153 (2013).

24. X. Wang, W. J. Eagen, J. C. Lee, Orchestration of human macrophage NLRP3 inflammasome activation by *Staphylococcus aureus* extracellular vesicles. *Proc. Natl. Acad. Sci. U.S.A.* **117**, 3174–3184 (2020).

25. S. Accarias, G. Lugo-Villarino, G. Foucras, O. Neyrolles, S. Boullier, G. Tabouret, Pyroptosis of resident macrophages differentially orchestrates inflammatory responses to *Staphylococcus aureus* in resistant and susceptible mice. *Eur. J. Immunol.* **45**, 794–806 (2015).

26. R. Yao, Y. Chen, H. Hao, Z. Guo, X. Cheng, Y. Ma, Q. Ji, X. Yang, Y. Wang, X. Li, Z. Wang, Pathogenic effects of inhibition of mTORC1/STAT3 axis facilitates *Staphylococcus aureus*-induced pyroptosis in human macrophages. *Cell Commun. Signal* **18**, 187 (2020).

27. T. S. Cohen, M. L. Boland, B. B. Boland, V. Takahashi, A. Tovchigrechko, Y. Lee, A. D. Wilde, M. J. Mazaitis, O. Jones-Nelson, C. Tkaczyk, R. Raja, C. K. Stover, B. R. Sellman, *Staphylococcus aureus* evades macrophage killing through NLRP3-dependent effects on mitochondrial trafficking. *Cell Rep.* **22**, 2431–2441 (2018).

28. I. Pastar, A. P. Sawaya, J. Marjanovic, J. L. Burgess, N. Strbo, K. E. Rivas, T. C. Wikramanayake, C. R. Head, R. C. Stone, I. Jozic, O. Stojadinovic, E. Y. Kornfeld, R. S. Kirsner, H. Lev-Tov, M. Tomic-Canic, Intracellular *Staphylococcus aureus* triggers pyroptosis and contributes to inhibition of healing due to perforin-2 suppression. *J. Clin. Invest.* **131**, e133727 (2021).

29. S. B. Kovacs, E. A. Miao, Gasdermins: Effectors of pyroptosis. *Trends Cell Biol.* **27**, 673–684 (2017).

30. P. Orning, D. Weng, K. Starheim, D. Ratner, Z. Best, B. Lee, A. Brooks, S. Xia, H. Wu, M. A. Kelliher, S. B. Berger, P. J. Gough, J. Bertin, M. M. Proulx, J. D. Goguen, N. Kayagaki, K. A. Fitzgerald, E. Lien, Pathogen blockade of TAK1 triggers caspase-8-dependent cleavage of gasdermin D and cell death. *Science* **362**, 1064–1069 (2018).

31. S. M. Man, T.-D. Kanneganti, Converging roles of caspases in inflammasome activation, cell death and innate immunity. *Nat. Rev. Immunol.* **16**, 7–21 (2016).

32. S. M. Man, R. Karki, T.-D. Kanneganti, Molecular mechanisms and functions of pyroptosis, inflammatory caspases and inflammasomes in infectious diseases. *Immunol. Rev.* **277**, 61–75 (2017).

33. J. Shi, W. Gao, F. Shao, Pyroptosis: Gasdermin-mediated programmed necrotic cell death. *Trends Biochem. Sci.* **42**, 245–254 (2017).

34. C. Kebaier, R. R. Chamberland, I. C. Allen, X. Gao, P. M. Broglie, J. D. Hall, C. Jania, C. M. Doerschuk, S. L. Tilley, J. A. Duncan, *Staphylococcus aureus* α-hemolysin mediates virulence in a murine model of severe pneumonia through activation of the NLRP3 inflammasome. *J Infect Dis* **205**, 807–817 (2012).

35. B. G. J. Surewaard, J. F. Deniset, F. J. Zemp, M. Amrein, M. Otto, J. Conly, A. Omri, R. M. Yates, P. Kubes, Identification and treatment of the *Staphylococcus aureus* reservoir in vivo. *J. Exp. Med.* **213**, 1141–1151 (2016).

36. V. K. Yajjala, V. C. Thomas, C. Bauer, T. D. Scherr, K. J. Fischer, P. D. Fey, K. W. Bayles, T. Kielian, K. Sun, Resistance to acute macrophage killing promotes airway fitness of prevalent community-acquired *Staphylococcus aureus* strains. *J. Immunol.* **196**, 4196–4203 (2016).

37. F. J. Martin, D. Parker, B. S. Harfenist, G. Soong, A. Prince, Participation of $CD11c^+$ leukocytes in methicillin-resistant *Staphylococcus aureus* clearance from the lung. *Infect. Immun.* **79**, 1898–1904 (2011).

38. Y. Li, X. Wang, The role of cullin4B in human cancers. *Exp. Hematol. Oncol.* **6**, 17 (2017).

39. Y. Song, P. Li, L. Qin, Z. Xu, B. Jiang, C. Ma, C. Shao, Y. Gong, CUL4B negatively regulates Toll-like receptor-triggered proinflammatory responses by repressing Pten transcription. *Cell. Mol. Immunol.* **18**, 339–349 (2021).

40. B. Jiang, W. Zhao, J. Yuan, Y. Qian, W. Sun, Y. Zou, C. Guo, B. Chen, C. Shao, Y. Gong, Lack of Cul4b, an E3 ubiquitin ligase component, leads to embryonic lethality and abnormal placental development. *PLOS ONE* **7**, e37070 (2012).

41. L. Liu, Y. Yin, Y. Li, L. Prevedel, E. H. Lacy, L. Ma, P. Zhou, Essential role of the CUL4B ubiquitin ligase in extra-embryonic tissue development during mouse embryogenesis. *Cell Res.* **22**, 1258–1269 (2012).

42. P. Li, Y. Song, W. Zan, L. Qin, S. Han, B. Jiang, H. Dou, C. Shao, Y. Gong, Lack of CUL4B in adipocytes promotes PPARγ-mediated adipose tissue expansion and insulin sensitivity. *Diabetes* **66**, 300–313 (2017).

43. P. S. Tarpey, F. L. Raymond, S. O'Meara, S. Edkins, J. Teague, A. Butler, E. Dicks, C. Stevens, C. Tofts, T. Avis, S. Barthorpe, G. Buck, J. Cole, K. Gray, K. Halliday, R. Harrison, K. Hills, A. Jenkinson, D. Jones, A. Menzies, T. Mironenko, J. Perry, K. Raine, D. Richardson, R. Shepherd, A. Small, J. Varian, S. West, S. Widaa, U. Mallya, J. Moon, Y. Luo, S. Holder, S. F. Smithson, J. A. Hurst, J. Clayton-Smith, B. Kerr, J. Boyle, M. Shaw, L. Vandeleur, J. Rodriguez, R. Slaugh, D. F. Easton, R. Wooster, M. Bobrow, A. K. Srivastava, R. E. Stevenson, C. E. Schwartz, G. Turner, J. Gecz, P. A. Futreal, M. R. Stratton, M. Partington, Mutations in CUL4B, which encodes a ubiquitin E3 ligase subunit, cause an

X-linked mental retardation syndrome associated with aggressive outbursts, seizures, relative macrocephaly, central obesity, hypogonadism, pes cavus, and tremor. *Am. J. Hum. Genet.* **80**, 345–352 (2007).

44. Y. Zou, Q. Liu, B. Chen, X. Zhang, C. Guo, H. Zhou, J. Li, G. Gao, Y. Guo, C. Yan, J. Wei, C. Shao, Y. Gong, Mutation in CUL4B, which encodes a member of cullin-RING ubiquitin ligase complex, causes X-linked mental retardation. *Am. J. Hum. Genet.* **80**, 561–566 (2007).

45. C.-Y. Lin, C.-Y. Chen, C.-H. Yu, I.-S. Yu, S.-R. Lin, J.-T. Wu, Y.-H. Lin, P.-L. Kuo, J.-C. Wu, S.-W. Lin, Human X-linked intellectual disability factor CUL4B is required for post-meiotic sperm development and male fertility. *Sci. Rep.* **6**, 20227 (2016).

46. Y. Yin, L. Liu, C. Yang, C. Lin, G. M. Veith, C. Wang, P. Sutovsky, P. Zhou, L. Ma, Cell autonomous and nonautonomous function of CUL4B in mouse spermatogenesis. *J. Biol. Chem.* **291**, 6923–6935 (2016).

47. Y. Fan, X. Huo, B. Guo, X. Zhang, Y. Yang, J. Lian, X. Meng, Y. Shao, Y. Zou, H. Guo, H. Wang, G. Sun, H. Dou, J. Wang, C. Shao, Y. Gong, H. Hu, Cullin 4b-RING ubiquitin ligase targets IRGM1 to regulate Wnt signaling and intestinal homeostasis. *Cell Death Differ.* **29**, 1673–1688 (2022).

48. H. Hu, Y. Yang, Q. Ji, W. Zhao, B. Jiang, R. Liu, J. Yuan, Q. Liu, X. Li, Y. Zou, C. Shao, Y. Shang, Y. Wang, Y. Gong, CRL4B catalyzes H2AK119 monoubiquitination and coordinates with PRC2 to promote tumorigenesis. *Cancer Cell* **22**, 781–795 (2012).

49. Y. Qian, J. Yuan, H. Hu, Q. Yang, J. Li, S. Zhang, B. Jiang, C. Shao, Y. Gong, The CUL4B/AKT/β-catenin axis restricts the accumulation of myeloid-derived suppressor cells to prohibit the establishment of a tumor-permissive microenvironment. *Cancer Res.* **75**, 5070–5083 (2015).

50. Y. Yang, R. Liu, R. Qiu, Y. Zheng, W. Huang, H. Hu, Q. Ji, H. He, Y. Shang, Y. Gong, Y. Wang, CRL4B promotes tumorigenesis by coordinating with SUV39H1/HP1/DNMT3A in DNA methylation-based epigenetic silencing. *Oncogene* **34**, 104–118 (2015).

51. Y. Wang, M. Manzi, A. Feswick, L. Renshaw, P. M. Oliver, S. A. Tibbetts, E. K. Moser, B cell expression of E3 ubiquitin ligase Cul4b promotes chronic gammaherpesvirus infection in vivo. *J. Virol.* **97**, e0100823 (2023).

52. H. Shan, B. Wang, X. Zhang, H. Song, X. Li, Y. Zou, B. Jiang, H. Hu, H. Dou, C. Shao, L. Gao, C. Ma, X. Yang, X. Liang, Y. Gong, CUL4B facilitates HBV replication by promoting HBx stabilization. *Cancer Biol. Med.* **19**, 120–131 (2021).

53. M.-H. Hung, Y.-R. Jian, C.-C. Tsao, S.-W. Lin, Y.-H. Chuang, Enhanced LPS-induced peritonitis in mice deficiency of cullin 4B in macrophages. *Genes Immun.* **15**, 404–412 (2014).

54. Z. Xu, L. Li, Y. Qian, Y. Song, L. Qin, Y. Duan, M. Wang, P. Li, B. Jiang, C. Ma, C. Shao, Y. Gong, Upregulation of IL-6 in CUL4B-deficient myeloid-derived suppressive cells increases the aggressiveness of cancer cells. *Oncogene* **38**, 5860–5872 (2019).

55. S. Jin, Y. Song, L. Zhou, W. Jiang, L. Qin, Y. Wang, R. Yu, Y. Liu, Y. Diao, F. Zhang, K. Liu, P. Li, H. Hu, B. Jiang, W. Tang, F. Yi, Y. Gong, G. Liu, G. Sun, Depletion of CUL4B in macrophages ameliorates diabetic kidney disease via miR-194-5p/ITGA9 axis. *Cell Rep.* **42**, 112550 (2023).

56. J. R. Pfeiffer, S. A. Brooks, Cullin 4B is recruited to tristetraprolin-containing messenger ribonucleoproteins and regulates TNF-α mRNA polysome loading. *J. Immunol.* **188**, 1828–1839 (2012).

57. Y. Yin, L. Zhang, Y. Zeng, D. Chen, H. Guan, G. Ran, K. Du, Histone ubiquitination-related gene CUL4B promotes lung adenocarcinoma progression and cisplatin resistance. *Front. Genet.* **14**, 1242137 (2023).

58. K. Liu, X. Hao, Y. Gao, Z. Cao, M. Hou, L. Qin, Y. Song, M. Wang, B. Jiang, Q. Liu, Y. Zou, Y. Gong, G. Liu, G. Sun, CUL4B protects kidneys from acute injury by restraining p53/PAI-1 signaling. *Cell Death Dis.* **15**, 915 (2024).

59. H. Song, B. Liu, W. Huai, Z. Yu, W. Wang, J. Zhao, L. Han, G. Jiang, L. Zhang, C. Gao, W. Zhao, The E3 ubiquitin ligase TRIM31 attenuates NLRP3 inflammasome activation by promoting proteasomal degradation of NLRP3. *Nat. Commun.* **7**, 13727 (2016).

60. J. Tang, S. Tu, G. Lin, H. Guo, C. Yan, Q. Liu, L. Huang, N. Tang, Y. Xiao, R. M. Pope, M. V. S. Rajaram, A. O. Amer, B. M. Ahmer, J. S. Gunn, D. J. Wozniak, L. Tao, V. Coppola, L. Zhang, W. Y. Langdon, J. B. Torrelles, S. Lipkowitz, J. Zhang, Sequential ubiquitination of NLRP3 by RNF125 and Cbl-b limits inflammasome activation and endotoxemia. *J. Exp. Med.* **217**, e20182091 (2020).

61. Y. Yan, W. Jiang, L. Liu, X. Wang, C. Ding, Z. Tian, R. Zhou, Dopamine controls systemic inflammation through inhibition of NLRP3 inflammasome. *Cell* **160**, 62–73 (2015).

62. S. Han, T. B. Lear, J. A. Jerome, S. Rajbhandari, C. A. Snavely, D. L. Gulick, K. F. Gibson, C. Zou, B. B. Chen, R. K. Mallampalli, Lipo-polysaccharide primes the NALP3 inflammasome by inhibiting its ubiquitination and degradation mediated by the SCFFBXL2 E3 ligase. *J. Biol. Chem.* **290**, 18124–18133 (2015).

63. A. Kawashima, T. Karasawa, K. Tago, H. Kimura, R. Kamata, F. Usui-Kawanishi, S. Watanabe, S. Ohta, M. Funakoshi-Tago, K. Yanagisawa, T. Kasahara, K. Suzuki, M. Takahashi, ARIH2 ubiquitinates NLRP3 and negatively regulates NLRP3 inflammasome activation in macrophages. *J. Immunol.* **199**, 3614–3622 (2017).

64. D. Wang, Y. Zhang, X. Xu, J. Wu, Y. Peng, J. Li, R. Luo, L. Huang, L. Liu, S. Yu, N. Zhang, B. Lu, K. Zhao, YAP promotes the activation of NLRP3 inflammasome via blocking K27-linked polyubiquitination of NLRP3. *Nat. Commun.* **12**, 2674 (2021).

65. P. Wan, Q. Zhang, W. Liu, Y. Jia, S. Ai, T. Wang, W. Wang, P. Pan, G. Yang, Q. Xiang, S. Huang, Q. Yang, W. Zhang, F. Liu, Q. Tan, W. Zhang, K. Wu, Y. Liu, J. Wu, Cullin1 binds and promotes NLRP3 ubiquitination to repress systematic inflammasome activation. *FASEB J.* **33**, 5793–5807 (2019).

66. F. Humphries, R. Bergin, R. Jackson, N. Delagic, B. Wang, S. Yang, A. V. Dubois, R. J. Ingram, P. N. Moynagh, The E3 ubiquitin ligase Pellino2 mediates priming of the NLRP3 inflammasome. *Nat. Commun.* **9**, 1560 (2018).

67. E. G. Vozza, A. M. Kelly, C. M. Daly, S. A. O'Rourke, S. R. Carlile, B. Morris, A. Dunne, R. M. McLoughlin, Type 1 interferons promote *Staphylococcus aureus* nasal colonization by inducing phagocyte apoptosis. *Cell Death Discov.* **10**, 403 (2024).

68. F. J. Martin, M. I. Gomez, D. M. Wetzel, G. Memmi, M. O'Seaghdha, G. Soong, C. Schindler, A. Prince, *Staphylococcus aureus* activates type I IFN signaling in mice and humans through the Xr repeated sequences of protein A. *J. Clin. Invest.* **119**, 1931–1939 (2009).

69. D. Parker, P. J. Planet, G. Soong, A. Narechania, A. Prince, Induction of type I interferon signaling determines the relative pathogenicity of *Staphylococcus aureus* strains. *PLOS Pathog.* **10**, e1003951 (2014).

70. M. B. Reynolds, B. Klein, M. J. McFadden, N. K. Judge, H. E. Navarrete, B. C. Michmerhuizen, D. Awad, T. L. Schultz, P. W. Harms, L. Zhang, T. R. O'Meara, J. Z. Sexton, C. A. Lyssiotis, J. M. Kahlenberg, M. X. O'Riordan, Type I interferon governs immunometabolic checkpoints that coordinate inflammation during Staphylococcal infection. *Cell Rep.* **43**, 114607 (2024).

71. S. Jackson, Y. Xiong, CRL4s: The CUL4-RING E3 ubiquitin ligases. *Trends Biochem. Sci.* **34**, 562–570 (2009).

72. O. Goldmann, M. Rohde, G. S. Chhatwal, E. Medina, Role of macrophages in host resistance to group A streptococci. *Infect. Immun.* **72**, 2956–2963 (2004).

73. R. S. Doster, J. A. Sutton, L. M. Rogers, D. M. Aronoff, J. A. Gaddy, Streptococcus agalactiae induces placental macrophages to release extracellular traps loaded with tissue remodeling enzymes via an oxidative burst-dependent mechanism. *MBio* **9**, e02084-18 (2018).

74. S. Müller, A. Faulhaber, C. Sieber, D. Pfeifer, T. Hochberg, M. Gansz, S. D. Deshmukh, S. Dauth, K. Brix, P. Saftig, C. Peters, P. Henneke, T. Reinheckel, The endolysosomal cysteine cathepsins L and K are involved in macrophage-mediated clearance of *Staphylococcus aureus* and the concomitant cytokine induction. *FASEB J.* **28**, 162–175 (2014).

75. M. Deshmukh, S. Subhash, Z. Hu, M. Mohammad, A. Jarneborn, R. Pullerits, T. Jin, P. K. Kopparapu, Gene expression of *S100a8/a9* predicts *Staphylococcus aureus*-induced septic arthritis in mice. *Front. Microbiol.* **14**, 1146694 (2023).

76. W. Skronska-Wasek, S. Durlanik, H. Q. Le, V. Schroeder, K. Kitt, J. P. Garnett, S. Pflanz, The antimicrobial peptide S100A8/A9 produced by airway epithelium functions as a potent and direct regulator of macrophage phenotype and function. *Eur. Respir. J.* **59**, 2002732 (2022).

77. T. Kawai, S. Akira, Toll-like receptor downstream signaling. *Arthritis Res. Ther.* **7**, 12–19 (2005).

78. V. S. Gadepalli, H. Kim, Y. Liu, T. Han, L. Cheng, XDeathDB: A visualization platform for cell death molecular interactions. *Cell Death Dis.* **12**, 1156 (2021).

79. I. Diamant, D. J. B. Clarke, J. E. Evangelista, N. Lingam, A. Ma'ayan, Harmonizome 3.0: Integrated knowledge about genes and proteins from diverse multi-omics resources. *Nucleic Acids Res.* **53**, D1016–D1028 (2025).

80. A. D. Rouillard, G. W. Gundersen, N. F. Fernandez, Z. Wang, C. D. Monteiro, M. G. Mcdermott, A. Ma'ayan, *The Harmonizome: A Collection of Processed Datasets Gathered to Serve and Mine Knowledge about Genes and Proteins. Database* (Oxford, 2016).

81. K. Schroder, J. Tschopp, The inflammasomes. *Cell* **140**, 821–832 (2010).

82. N. Truongvan, S. Li, M. Misra, M. Kuhn, H. Schindelin, Structures of UBA6 explain its dual specificity for ubiquitin and FAT10. *Nat. Commun.* **13**, 4789 (2022).

83. A. Y. Kabakov, K. Roder, P. Bronk, N. N. Turan, S. Dhakal, M. Zhong, Y. Lu, Z. A. Zeltzer, Y. B. Najman-Licht, A. Karma, G. Koren, E3 ubiquitin ligase rififylin has yin and yang

effects on rabbit cardiac transient outward potassium currents (Ito) and corresponding channel proteins. *J. Biol. Chem.* **300**, 105759 (2024).

84. T. Okiyoneda, G. Veit, R. Sakai, M. Aki, T. Fujihara, M. Higashi, S. Susuki-Miyata, M. Miyata, N. Fukuda, A. Yoshida, H. Xu, P. M. Apaja, G. L. Lukacs, Chaperone-independent peripheral quality control of CFTR by RFFL E3 ligase. *Dev. Cell* **44**, 694–708.e7 (2018).

85. K. Keusekotten, P. R. Elliott, L. Glockner, B. K. Fiil, R. B. Damgaard, Y. Kulathu, T. Wauer, M. K. Hospenthal, M. Gyrd-Hansen, D. Krappmann, K. Hofmann, D. Komander, OTULIN antagonizes LUBAC signaling by specifically hydrolyzing Met1-linked polyubiquitin. *Cell* **153**, 1312–1326 (2013).

86. B. K. Fiil, R. B. Damgaard, S. A. Wagner, K. Keusekotten, M. Fritsch, S. Bekker-Jensen, N. Mailand, C. Choudhary, D. Komander, M. Gyrd-Hansen, OTULIN restricts Met1-linked ubiquitination to control innate immune signaling. *Mol. Cell* **50**, 818–830 (2013).

87. X. Gao, X. Yan, Q. Zhang, Y. Yin, J. Cao, CD5L contributes to the pathogenesis of methicillin-resistant *Staphylococcus aureus*-induced pneumonia. *Int. Immunopharmacol.* **72**, 40–47 (2019).

88. A. Ruiz-Sanmartín, V. Ribas, D. Suñol, L. Chiscano-Camón, C. Palmada, I. Bajaña, N. Larrosa, J. J. González, N. Canela, R. Ferrer, J. C. Ruiz-Rodríguez, Characterization of a proteomic profile associated with organ dysfunction and mortality of sepsis and septic shock. *PLOS ONE* **17**, e0278708 (2022).

89. J. H. Shin, N. Bozadjieva-Kramer, R. J. Seeley, Reg3γ: Current understanding and future therapeutic opportunities in metabolic disease. *Exp. Mol. Med.* **55**, 1672–1677 (2023).

90. M. Gironella, C. Calvo, A. Fernández, D. Closa, J. L. Iovanna, J. Rosello-Catafau, E. Folch-Puy, Reg3β deficiency impairs pancreatic tumor growth by skewing macrophage polarization. *Cancer Res.* **73**, 5682–5694 (2013).

91. R. Shindo, T. Katagiri, S. Komazawa-Sakon, M. Ohmuraya, W. Takeda, Y. Nakagawa, N. Nakagata, T. Sakuma, T. Yamamoto, C. Nishiyama, T. Nishina, S. Yamazaki, H. Kameda, H.

Nakano, Regenerating islet-derived protein (Reg)3β plays a crucial role in attenuation of ileitis and colitis in mice. *Biochem. Biophys. Rep.* **21**, 100738 (2020).

92. H. Burgmann, S. Winkler, G. J. Locker, E. Presterl, K. Laczika, T. Staudinger, S. Knapp, F. Thalhammer, C. Wenisch, K. Zedwitz-Liebenstein, M. Frass, W. Graninger, Increased serum concentration of soluble CD14 is a prognostic marker in gram-positive sepsis. *Clin. Immunol. Immunopathol.* **80**, 307–310 (1996).

93. M. Liu, K. Liu, D. Cheng, B. Zheng, S. Li, Z. Mo, The regulatory role of NLRX1 in innate immunity and human disease. *Cytokine* **160**, 156055 (2022).

94. D. Supino, L. Minute, A. Mariancini, F. Riva, E. Magrini, C. Garlanda, Negative regulation of the IL-1 system by IL-1R2 and IL-1R8: Relevance in pathophysiology and disease. *Front. Immunol.* **13**, 804641 (2022).

95. M. Molgora, D. Supino, A. Mantovani, C. Garlanda, Tuning inflammation and immunity by the negative regulators IL-1R2 and IL-1R8. *Immunol. Rev.* **281**, 233–247 (2018).

96. Q. Zhang, C. Wang, S. Li, Y. Li, M. Chen, Y. Hu, Screening of core genes prognostic for sepsis and construction of a ceRNA regulatory network. *BMC Med. Genomics* **16**, 37 (2023).

97. M. A. Kovach, K. A. Stringer, R. Bunting, X. Wu, L. San Mateo, M. W. Newstead, R. Paine, T. J. Standiford, Microarray analysis identifies IL-1 receptor type 2 as a novel candidate biomarker in patients with acute respiratory distress syndrome. *Respir. Res.* **16**, 29 (2015).

98. F. Meng, N. Sun, D. Liu, J. Jia, J. Xiao, H. Dai, BCL2L13: Physiological and pathological meanings. *Cell. Mol. Life Sci.* **78**, 2419–2428 (2021).

99. S. Banga, P. Gao, X. Shen, V. Fiscus, W.-X. Zong, L. Chen, Z.-Q. Luo, *Legionella pneumophila* inhibits macrophage apoptosis by targeting pro-death members of the Bcl2 protein family. *Proc. Natl. Acad. Sci. U.S.A.* **104**, 5121–5126 (2007).

100. M. Speir, A. Vogrin, A. Seidi, G. Abraham, S. Hunot, Q. Han, G. W. Dorn II, S. L. Masters, R. A. Flavell, J. E. Vince, T. Naderer, *Legionella pneumophila* strain 130b evades

macrophage cell death independent of the effector SidF in the absence of flagellin. *Front. Cell. Infect. Microbiol.* **7**, 35 (2017).

101. A. Khandagale, B. Lazzaretto, G. Carlsson, M. Sundin, S. Shafeeq, U. Römling, B. Fadeel, JAGN1 is required for fungal killing in neutrophil extracellular traps: Implications for severe congenital neutropenia. *J. Leukoc. Biol.* **104**, 1199–1213 (2018).

102. J. Li, P. Xu, L. Wang, M. Feng, D. Chen, X. Yu, Y. Lu, Molecular biology of BPIFB1 and its advances in disease. *Ann. Transl. Med.* **8**, 651 (2020).

103. K. Kitur, D. Parker, P. Nieto, D. S. Ahn, T. S. Cohen, S. Chung, S. Wachtel, S. Bueno, A. Prince, Toxin-induced necroptosis is a major mechanism of *Staphylococcus aureus* lung damage. *PLOS Pathog.* **11**, e1004820 (2015).

104. B. Voisin, V. Nadella, T. Doebel, S. Goel, K. Sakamoto, O. Ayush, J.-H. Jo, M. C. Kelly, T. Kobayashi, J. X. Jiang, Y. Hu, C. Yan, K. Nagao, Macrophage-mediated extracellular matrix remodeling controls host *Staphylococcus aureus* susceptibility in the skin. *Immunity* **56**, 1561–1577.e9 (2023).

105. X. Chen, Y. Wang, A. Cappuccio, W.-S. Cheng, F. R. Zamojski, V. D. Nair, C. M. Miller, A. B. Rubenstein, G. Nudelman, A. Tadych, C. L. Theesfeld, A. Vornholt, M.-C. George, F. Ruffin, M. Dagher, D. G. Chawla, A. Soares-Schanoski, R. R. Spurbeck, L. C. Ndhlovu, R. Sebra, S. H. Kleinstein, A. G. Letizia, I. Ramos, V. G. Fowler Jr., C. W. Woods, E. Zaslavsky, O. G. Troyanskaya, S. C. Sealfon, Mapping disease regulatory circuits at cell-type resolution from single-cell multiomics data. *Nat. Comput. Sci.* **3**, 644–657 (2023).

106. J. Alves, M. Vrieling, N. Ring, G. Yebra, A. Pickering, T. K. Prajsnar, S. A. Renshaw, J. R. Fitzgerald, Experimental evolution of *Staphylococcus aureus* in macrophages: Dissection of a conditional adaptive trait promoting intracellular survival. *MBio* **15**, e0034624 (2024).

107. Y. Li, H. Huang, B. Liu, Y. Zhang, X. Pan, X.-Y. Yu, Z. Shen, Y.-H. Song, Inflammasomes as therapeutic targets in human diseases. *Signal Transduct. Target. Ther.* **6**, 247 (2021).

108. H. Ashida, H. Mimuro, M. Ogawa, T. Kobayashi, T. Sanada, M. Kim, C. Sasakawa, Cell death and infection: A double-edged sword for host and pathogen survival. *J. Cell Biol.* **195**, 931–942 (2011).

109. Y. Li, R. Qiang, Z. Cao, Q. Wu, J. Wang, W. Lyu, NLRP3 inflammasomes: Dual function in infectious diseases. *J. Immunol.* **213**, 407–417 (2024).

110. S. Paudel, R. Kumar, K. A. Rogers, Y. Saini, S. Patial, R. Kulkarni, The NLRP3 inflammasome is dispensable in methicillin-resistant *Staphylococcus aureus* urinary tract infection. *Pathogens* **13**, 106 (2024).

111. E. Carballo, W. S. Lai, P. J. Blackshear, Evidence that tristetraprolin is a physiological regulator of granulocyte-macrophage colony-stimulating factor messenger RNA deadenylation and stability. *Blood* **95**, 1891–1899 (2000).

112. E. A. Van Tubergen, R. Banerjee, M. Liu, R. Vander Broek, E. Light, S. Kuo, S. E. Feinberg, A. L. Willis, G. Wolf, T. Carey, C. Bradford, M. Prince, F. P. Worden, K. L. Kirkwood, N. J. D'Silva, Inactivation or loss of TTP promotes invasion in head and neck cancer via transcript stabilization and secretion of MMP9, MMP2, and IL-6. *Clin. Cancer Res.* **19**, 1169–1179 (2013).

113. J.-G. Kang, M. J. Amar, A. T. Remaley, J. Kwon, P. J. Blackshear, P.-Y. Wang, P. M. Hwang, Zinc finger protein tristetraprolin interacts with CCL3 mRNA and regulates tissue inflammation. *J. Immunol.* **187**, 2696–2701 (2011).

114. M. Bros, N. Wiechmann, V. Besche, J. Art, A. Pautz, S. Grabbe, H. Kleinert, A. B. Reske-Kunz, The RNA binding protein tristetraprolin influences the activation state of murine dendritic cells. *Mol. Immunol.* **47**, 1161–1170 (2010).

115. G. I. Vladimer, R. Marty-Roix, S. Ghosh, D. Weng, E. Lien, Inflammasomes and host defenses against bacterial infections. *Curr. Opin. Microbiol.* **16**, 23–31 (2013).

116. K. Shimada, T. R. Crother, J. Karlin, J. Dagvadorj, N. Chiba, S. Chen, V. K. Ramanujan, A. J. Wolf, L. Vergnes, D. M. Ojcius, A. Rentsendorj, M. Vargas, C. Guerrero, Y. Wang, K. A.

Fitzgerald, D. M. Underhill, T. Town, M. Arditi, Oxidized mitochondrial DNA activates the NLRP3 inflammasome during apoptosis. *Immunity* **36**, 401–414 (2012).

117. R. I. Campden, Y. Zhang, The role of lysosomal cysteine cathepsins in NLRP3 inflammasome activation. *Arch. Biochem. Biophys.* **670**, 32–42 (2019).

118. S. Paik, J. K. Kim, P. Silwal, C. Sasakawa, E.-K. Jo, An update on the regulatory mechanisms of NLRP3 inflammasome activation. *Cell. Mol. Immunol.* **18**, 1141–1160 (2021).

119. L. Verboom, E. Hoste, G. van Loo, OTULIN in NF-κB signaling, cell death, and disease. *Trends Immunol.* **42**, 590–603 (2021).

120. K. Wu, K. Q. Huynh, I. Lu, M. Moustakim, H. Miao, C. Yu, M. J. Haeusgen, B. D. Hopkins, L. Huang, N. Zheng, R. Sanchez, R. J. DeVita, Z.-Q. Pan, Inhibitors of cullin-RING E3 ubiquitin ligase 4 with antitumor potential. *Proc. Natl. Acad. Sci. U.S.A.* **118**, e2007328118 (2021).

121. Y. Zhou, B. Zhou, L. Pache, M. Chang, A. H. Khodabakhshi, O. Tanaseichuk, C. Benner, S. K. Chanda, Metascape provides a biologist-oriented resource for the analysis of systems-level datasets. *Nat. Commun.* **10**, 1523 (2019).

122. E. W. Deutsch, N. Bandeira, V. Sharma, Y. Perez-Riverol, J. J. Carver, D. J. Kundu, D. García-Seisdedos, A. F. Jarnuczak, S. Hewapathirana, B. S. Pullman, J. Wertz, Z. Sun, S. Kawano, S. Okuda, Y. Watanabe, H. Hermjakob, B. MacLean, M. J. MacCoss, Y. Zhu, Y. Ishihama, J. A. Vizcaíno, The ProteomeXchange consortium in 2020: Enabling "big data" approaches in proteomics. *Nucleic Acids Res.* **48**, D1145–D1152 (2020).

123. J. Sun, S. Uchiyama, J. Olson, Y. Morodomi, I. Cornax, N. Ando, Y. Kohno, M. M. T. Kyaw, B. Aguilar, N. M. Haste, S. Kanaji, T. Kanaji, W. E. Rose, G. Sakoulas, J. D. Marth, V. Nizet, Repurposed drugs block toxin-driven platelet clearance by the hepatic Ashwell-Morell receptor to clear *Staphylococcus aureus* bacteremia. *Sci. Transl. Med.* **13**, eabd6737 (2021).

124. J. M. Voyich, K. R. Braughton, D. E. Sturdevant, A. R. Whitney, B. Saïd-Salim, S. F. Porcella, R. D. Long, D. W. Dorward, D. J. Gardner, B. N. Kreiswirth, J. M. Musser, F. R. DeLeo, Insights into mechanisms used by *Staphylococcus aureus* to avoid destruction by human neutrophils. *J. Immunol.* **175**, 3907–3919 (2005).

125. S. Chatellier, N. Ihendyane, R. G. Kansal, F. Khambaty, H. Basma, A. Norrby-Teglund, D. E. Low, A. McGeer, M. Kotb, Genetic relatedness and superantigen expression in group A streptococcus serotype M1 isolates from patients with severe and nonsevere invasive diseases. *Infect. Immun.* **68**, 3523–3534 (2000).

126. C. B. Wilson, W. M. Weaver, Comparative susceptibility of group B streptococci and *Staphylococcus aureus* to killing by oxygen metabolites. *J Infect Dis* **152**, 323–329 (1985).

127. I. Rodrigues Lopes, L. M. Alcantara, R. J. Silva, J. Josse, E. P. Vega, A. M. Cabrerizo, M. Bonhomme, D. Lopez, F. Laurent, F. Vandenesch, M. Mano, A. Eulalio, Microscopy-based phenotypic profiling of infection by *Staphylococcus aureus* clinical isolates reveals intracellular lifestyle as a prevalent feature. *Nat. Commun.* **13**, 7174 (2022).

128. L. Chen, Z. Wang, T. Xu, H. Ge, F. Zhou, X. Zhu, X. Li, D. Qu, C. Zheng, Y. Wu, K. Zhao, The role of *graRS* in regulating virulence and antimicrobial resistance in methicillin-resistant *Staphylococcus aureus*. *Front. Microbiol.* **12**, 727104 (2021).

129. J. Villén, S. P. Gygi, The SCX/IMAC enrichment approach for global phosphorylation analysis by mass spectrometry. *Nat. Protoc.* **3**, 1630–1638 (2008).

130. J. D. Lapek Jr., R. H. Mills, J. M. Wozniak, A. Campeau, R. H. Fang, X. Wei, K. van de Groep, A. Perez-Lopez, N. M. van Sorge, M. Raffatellu, R. Knight, L. Zhang, D. J. Gonzalez, Defining host responses during systemic bacterial infection through construction of a murine organ proteome atlas. *Cell Syst.* **6**, 579–592.e4 (2018).

131. W. Haas, B. K. Faherty, S. A. Gerber, J. E. Elias, S. A. Beausoleil, C. E. Bakalarski, J. L. Villén, S. P. Gygi, Haas optimization and use of peptide mass measurement accuracy in shotgun proteomics. *Mol. Cell. Proteomics* **5**, 1326–1337 (2006).

132. D. Wessel, U. I. Flügge, A method for the quantitative recovery of protein in dilute solution in the presence of detergents and lipids. *Anal. Biochem.* **138**, 141–143 (1984).

133. A. C. Tolonen, W. Haas, Quantitative proteomics using reductive dimethylation for stable isotope labeling. *J. Vis. Exp.* , 51416 (2014).

134. J. D. Lapek Jr., P. Greninger, R. Morris, A. Amzallag, I. Pruteanu-Malinici, C. H. Benes, W. Haas, Detection of dysregulated protein-association networks by high-throughput proteomics predicts cancer vulnerabilities. *Nat. Biotechnol.* **35**, 983–989 (2017).

135. A. Thompson, J. Schäfer, K. Kuhn, S. Kienle, J. Schwarz, G. Schmidt, T. Neumann, R. Johnstone, A. K. A. Mohammed, C. Hamon, Tandem mass tags: A novel quantification strategy for comparative analysis of complex protein mixtures by MS/MS. *Anal. Chem.* **75**, 1895–1904 (2003).

136. G. C. McAlister, E. L. Huttlin, W. Haas, L. Ting, M. P. Jedrychowski, J. C. Rogers, K. Kuhn, I. Pike, R. A. Grothe, J. D. Blethrow, S. P. Gygi, Increasing the multiplexing capacity of TMTs using reporter ion isotopologues with isobaric masses. *Anal. Chem.* **84**, 7469–7478 (2012).

137. G. C. McAlister, D. P. Nusinow, M. P. Jedrychowski, M. Wühr, E. L. Huttlin, B. K. Erickson, R. Rad, W. Haas, S. P. Gygi, MultiNotch MS3 enables accurate, sensitive, and multiplexed detection of differential expression across cancer cell line proteomes. *Anal. Chem.* **86**, 7150–7158 (2014).

138. J. K. Eng, A. L. McCormack, J. R. Yates, An approach to correlate tandem mass spectral data of peptides with amino acid sequences in a protein database. *J. Am. Soc. Mass Spectrom.* **5**, 976–989 (1994).

139. J. Peng, J. E. Elias, C. C. Thoreen, L. J. Licklider, S. P. Gygi, Evaluation of multidimensional chromatography coupled with tandem mass spectrometry (LC/LC-MS/MS) for large-scale protein analysis: The yeast proteome. *J. Proteome Res.* **2**, 43–50 (2003).

140. J. E. Elias, W. Haas, B. K. Faherty, S. P. Gygi, Comparative evaluation of mass spectrometry platforms used in large-scale proteomics investigations. *Nat. Methods* **2**, 667–675 (2005).

141. J. E. Elias, S. P. Gygi, Target-decoy search strategy for increased confidence in large-scale protein identifications by mass spectrometry. *Nat. Methods* **4**, 207–214 (2007).

142. E. L. Huttlin, M. P. Jedrychowski, J. E. Elias, T. Goswami, R. Rad, S. A. Beausoleil, J. Villén, W. Haas, M. E. Sowa, S. P. Gygi, A tissue-specific atlas of mouse protein phosphorylation and expression. *Cell* **143**, 1174–1189 (2010).

143. S. A. Beausoleil, J. Villén, S. A. Gerber, J. Rush, S. P. Gygi, A probability-based approach for high-throughput protein phosphorylation analysis and site localization. *Nat. Biotechnol.* **24**, 1285–1292 (2006).

144. L. Ting, R. Rad, S. P. Gygi, W. Haas, MS3 eliminates ratio distortion in isobaric multiplexed quantitative proteomics. *Nat. Methods* **8**, 937–940 (2011).

145. J. D. Lapek Jr., M. K. Lewinski, J. M. Wozniak, J. Guatelli, D. J. Gonzalez, Quantitative temporal viromics of an inducible HIV-1 model yields insight to global host targets and phospho-dynamics associated with protein vpr. *Mol. Cell. Proteomics* **16**, 1447–1461 (2017).

146. F. Supek, M. Bošnjak, N. Škunca, T. Šmuc, REVIGO summarizes and visualizes long lists of gene ontology terms. *PLOS ONE* **6**, e21800 (2011).

147. R. Vishnubalaji, V. Sasidharan Nair, K. Ouararhni, E. Elkord, N. M. Alajez, Integrated transcriptome and pathway analyses revealed multiple activated pathways in breast cancer. *Front. Oncol.* **9**, 910 (2019).

148. A. Krämer, J. Green, J. Pollard Jr., S. Tugendreich, Causal analysis approaches in ingenuity pathway analysis. *Bioinformatics* **30**, 523–530 (2014).

149. P. Shannon, A. Markiel, O. Ozier, N. S. Baliga, J. T. Wang, D. Ramage, N. Amin, B. Schwikowski, T. Ideker, Cytoscape: A software environment for integrated models of biomolecular interaction networks. *Genome Res.* **13**, 2498–2504 (2003).

150. A. Lex, N. Gehlenborg, H. Strobelt, R. Vuillemot, H. Pfister, UpSet: Visualization of intersecting sets. *IEEE Trans. Vis. Comput. Graph* **20**, 1983–1992 (2014).

151. M. Waskom, seaborn: Statistical data visualization. *J. Open Source Softw.* **6**, 3021 (2021).

152. D. Szklarczyk, A. L. Gable, K. C. Nastou, D. Lyon, R. Kirsch, S. Pyysalo, N. T. Doncheva, M. Legeay, T. Fang, P. Bork, L. J. Jensen, C. von Mering, Correction to "The STRING database in 2021: Customizable protein-protein networks, and functional characterization of user-uploaded gene/measurement sets". *Nucleic Acids Res.* **49**, 10800 (2021).

153. P. Ewels, M. Magnusson, S. Lundin, M. Käller, MultiQC: Summarize analysis results for multiple tools and samples in a single report. *Bioinformatics* **32**, 3047–3048 (2016).

154. A. M. Bolger, M. Lohse, B. Usadel, Trimmomatic: A flexible trimmer for Illumina sequence data. *Bioinformatics* **30**, 2114–2120 (2014).

155. A. Dobin, C. A. Davis, F. Schlesinger, J. Drenkow, C. Zaleski, S. Jha, P. Batut, M. Chaisson, T. R. Gingeras, STAR: Ultrafast universal RNA-seq aligner. *Bioinformatics* **29**, 15–21 (2013).

156. S. Anders, P. T. Pyl, W. Huber, HTSeq—A Python framework to work with high-throughput sequencing data. *Bioinformatics* **31**, 166–169 (2015).

157. H. Wickham, *Ggplot2: Elegant Graphics for Data Analysis* (Springer, 2009).

158. G. Yu, L.-G. Wang, Y. Han, Q.-Y. He, clusterProfiler: An R package for comparing biological themes among gene clusters. *OMICS* **16**, 284–287 (2012).

159. M. Johannessen, M. P. Delghandi, A. Rykx, M. Dragset, J. R. Vandenheede, J. Van Lint, U. Moens, Protein kinase D induces transcription through direct phosphorylation of the cAMP-response element-binding protein. *J. Biol. Chem.* **282**, 14777–14787 (2007).

160. E. K. McEachern, J. H. Hwang, K. M. Sladewski, S. Nicatia, C. Dewitz, D. P. Mathew, V. Nizet, L. E. Crotty Alexander, Analysis of the effects of cigarette smoke on staphylococcal virulence phenotypes. *Infect. Immun.* **83**, 2443–2452 (2015).

161. L. Käll, J. D. Canterbury, J. Weston, W. S. Noble, M. J. MacCoss, Semi-supervised learning for peptide identification from shotgun proteomics datasets. *Nat. Methods* **4**, 923–925 (2007).

162. M. Spivak, J. Weston, L. Bottou, L. Käll, W. S. Noble, Improvements to the percolator algorithm for peptide identification from shotgun proteomics data sets. *J. Proteome Res.* **8**, 3737–3745 (2009).

163. V. Demichev, C. B. Messner, S. I. Vernardis, K. S. Lilley, M. Ralser, DIA-NN: Neural networks and interference correction enable deep proteome coverage in high throughput. *Nat. Methods* **17**, 41–44 (2020).

164. Y. Hsiao, H. Zhang, G. X. Li, Y. Deng, F. Yu, H. Valipour Kahrood, J. R. Steele, R. B. Schittenhelm, A. I. Nesvizhskii, Analysis and visualization of quantitative proteomics data using FragPipe-Analyst. *J. Proteome Res.* **23**, 4303–4315 (2024).

165. M.-A. Gerault, L. Camoin, S. Granjeaud, DIAgui: A Shiny application to process the output from DIA-NN. *Bioinform. Adv.* **4**, vbae001 (2024).

166. G. K. Smyth, "Limma: Linear models for microarray data," in *Bioinformatics and Computational Biology Solutions using R and Bioconductor*, (Springer, 2005), pp. 397–420.

167. P. D. Thomas, D. Ebert, A. Muruganujan, T. Mushayahama, L.-P. Albou, H. Mi, PANTHER: Making genome-scale phylogenetics accessible to all. *Protein Sci.* **31**, 8–22 (2022).

168. H. Mi, A. Muruganujan, X. Huang, D. Ebert, C. Mills, X. Guo, P. D. Thomas, Protocol update for large-scale genome and gene function analysis with the PANTHER classification system (v.14.0). *Nat. Protoc.* **14**, 703–721 (2019).

169. J. Abramson, J. Adler, J. Dunger, R. Evans, T. Green, A. Pritzel, O. Ronneberger, L. Willmore, A. J. Ballard, J. Bambrick, S. W. Bodenstein, D. A. Evans, C.-C. Hung, M. O'Neill, D. Reiman, K. Tunyasuvunakool, Z. Wu, A. Žemgulytė, E. Arvaniti, C. Beattie, O. Bertolli, A. Bridgland, A. Cherepanov, M. Congreve, A. I. Cowen-Rivers, A. Cowie, M. Figurnov, F. B. Fuchs, H. Gladman, R. Jain, Y. A. Khan, C. M. R. Low, K. Perlin, A. Potapenko, P. Savy, S. Singh, A. Stecula, A. Thillaisundaram, C. Tong, S. Yakneen, E. D.

Zhong, M. Zielinski, A. Žídek, V. Bapst, P. Kohli, M. Jaderberg, D. Hassabis, J. M. Jumper, Accurate structure prediction of biomolecular interactions with AlphaFold 3. *Nature* **630**, 493–500 (2024).

170. F. Askarian, C.-M. Tsai, G. Cordara, R. H. Zurich, E. Bjånes, O. Golten, H. Vinther Sørensen, A. Kousha, A. Meier, E. Chikwati, J.-A. Bruun, J. A. Ludviksen, B. Choudhury, D. Trieu, S. Davis, P. K. T. Edvardsen, T. E. Mollnes, G. Y. Liu, U. Krengel, D. J. Conrad, G. Vaaje-Kolstad, V. Nizet, Immunization with lytic polysaccharide monooxygenase CbpD induces protective immunity against *Pseudomonas aeruginosa* pneumonia. *Proc. Natl. Acad. Sci. U.S.A.* **120**, e2301538120 (2023).

171. Y. Xiao, T.-H. Hsiao, U. Suresh, H.-I. H. Chen, X. Wu, S. E. Wolf, Y. Chen, A novel significance score for gene selection and ranking. *Bioinformatics* **30**, 801–807 (2014).

172. X. Wang, C. Song, D. Zhou, Y. Mei, W. Cai, R. Chen, J. Lv, H. Shi, Z. Liu, Exploring the therapeutic potential of puerarin on intervertebral disc degeneration by regulating apoptosis of nucleus pulposus cells. *JOR Spine* **7**, e70020 (2024).

173. L. Liu, R. Hui, T. Zeng, X. Yang, Q. Wu, T. Yang, CUL4B is a potential novel prognostic biomarker and is correlated with immune infiltrates in malignant pleural mesothelioma. *Int. J. Gen. Med.* **15**, 4613–4623 (2022).

174. S. Feng, M. C. Wierzbowski, K. Hrovat-Schaale, A. Dumortier, Y. Zhang, M. Zyulina, P. J. Baker, T. Reygaerts, A. Steiner, D. De Nardo, D. L. Narayanan, F. Milhavet, A. Pinzon-Charry, J. I. Arostegui, R. P. Khubchandani, M. Geyer, G. Boursier, S. L. Masters, Mechanisms of NLRP3 activation and inhibition elucidated by functional analysis of disease-associated variants. *Nat. Immunol.* **26**, 511–523 (2025).

175. W. Zhao, B. Jiang, H. Hu, S. Zhang, S. Lv, J. Yuan, Y. Qian, Y. Zou, X. Li, H. Jiang, F. Liu, C. Shao, Y. Gong, Lack of CUL4B leads to increased abundance of GFAP-positive cells that is mediated by PTGDS in mouse brain. *Hum. Mol. Genet.* **24**, 4686–4697 (2015).
